# Population size rescaling significantly biases outcomes of forward-in-time population genetic simulations

**DOI:** 10.1101/2024.04.07.588318

**Published:** 2024-04-18

**Authors:** Amjad Dabi, Daniel R. Schrider

**Affiliations:** Department of Genetics, University of North Carolina, Chapel Hill, North Carolina, USA

**Keywords:** forward simulation, rescaling, approximation error

## Abstract

Simulations are an essential tool in all areas of population genetic research, used in tasks such as the validation of theoretical analysis and the study of complex evolutionary models. Forward-in-time simulations are especially flexible, allowing for various types of natural selection, complex genetic architectures, and non-Wright-Fisher dynamics. However, their intense computational requirements can be prohibitive to simulating large populations and genomes. A popular method to alleviate this burden is to scale down the population size by some scaling factor while scaling up the mutation rate, selection coefficients, and recombination rate by the same factor. However, this rescaling approach may in some cases bias simulation results. To investigate the manner and degree to which rescaling impacts simulation outcomes, we carried out simulations with different demographic histories and distributions of fitness effects using several values of the rescaling factor, Q, and compared the deviation of key outcomes (fixation times, fixation probabilities, allele frequencies, and linkage disequilibrium) between the scaled and unscaled simulations. Our results indicate that scaling introduces substantial biases to each of these measured outcomes, even at small values of Q. Moreover, the nature of these effects depends on the evolutionary model and scaling factor being examined. While increasing the scaling factor tends to increase the observed biases, this relationship is not always straightforward, thus it may be difficult to know the impact of scaling on simulation outcomes *a priori*. However, it appears that for most models, only a small number of replicates was needed to accurately quantify the bias produced by rescaling for a given Q. In summary, while rescaling forward-in-time simulations may be necessary in many cases, researchers should be aware of the rescaling effect’s impact on simulation outcomes and consider investigating its magnitude in smaller scale simulations of the desired model(s) before selecting an appropriate value of Q.

## ARTICLE SUMMARY

Computer simulations are an indispensable tool for researchers studying the dynamics of population genetic models, but often require large computing resources and long run times. A widely used approximation alleviates these computational requirements by simulating a smaller population and increasing the values of other model parameters to compensate. However, this results in significant biases to important simulation outcomes. Fortunately, the direction and magnitude of these biases can be measured beforehand by carrying out a relatively small number of simulations.

## INTRODUCTION

Stochastic simulations are widely used in population genetics (Hoban et al. 2012; Peng et al. 2015; Adrion et al. 2020). Their uses include the study of the behavior of a wide variety of population genetic models, either when used on their own in simulation studies (Hudson 1983a; Charlesworth et al. 1993; Hermisson and Pennings 2005; Orr and Unckless 2008; Peischl et al. 2013; Zeng 2013) or when used to validate theoretical predictions (Morgan and Strobeck 1979; Braverman et al. 1995; Thornton 2019; Battey et al. 2020; Schrider 2020; Torres et al. 2020), the comparison of simulations to data to infer model parameters or choose between competing models (Tavare et al. 1997; Pritchard et al. 1999; Beaumont et al. 2002; Beaumont 2010; Csilléry et al. 2010), assessing the accuracy of inference methods (Pavlidis et al. 2013; Alachiotis and Pavlidis 2018; Galimberti et al. 2020), and the generation of training datasets for machine learning methods (Pavlidis et al. 2010; Ronen et al. 2013; Gao et al. 2016; Sheehan and Song 2016; Chan et al. 2018; Schrider and Kern 2018; Flagel et al. 2019; Mughal and DeGiorgio 2019; Caravagna et al. 2020; Sanchez et al. 2021; Smith et al. 2023). Given their wide range of research applications, considerable effort has been made to improve the accessibility, computational speed, and flexibility of population genetic simulations (Hudson 2002; Ewing and Hermisson 2010; Messer 2013; Kern and Schrider 2016; Kelleher et al. 2018; Haller and Messer 2023).

Two main modes of population genetic simulation are available to researchers: coalescent simulations, which trace the genealogy of a sample of individuals back in time until all segments of the chromosome have reached the sample’s MRCA, and forward-in-time simulations, which explicitly simulate an entire population of chromosomes evolving under the desired model. Coalescent simulations have been a mainstay of population genetics for the last few decades because of their computational efficiency (Liang et al. 2007; Carvajal-Rodriguez 2008; Kelleher et al. 2016), as they are often used to simulate only a relatively small sample of genomes rather than the entire population (Hudson 1983b), and jump backward from one event (e.g. coalescence and recombination) rather than simulating each generation of the evolutionary scenario.

While computationally efficient, coalescent simulations are relatively limited in the evolutionary scenarios that they can model, especially with respect to natural selection, and are therefore more commonly used for modeling demographic histories (see Ewing and Hermisson (2010); Kern and Schrider (2016); Baumdicker et al. (2022); Teshima and Innan (2009) for notable exceptions). Forward-in-time simulations, on the other hand, provide much greater flexibility (Peng and Kimmel 2005; Hernandez 2008; Neuenschwander et al. 2008; Thornton 2014; Haller and Messer 2017), giving users the ability to simulate more complex models involving not only demographic changes, but several types of natural selection, and scenarios that fall outside the bounds of the Wright-Fisher model (Haller and Messer 2019). Unfortunately, forward-in-time simulations can require a large amount of computational power and memory because the entire population is simulated for each generation of the desired scenario, rendering simulations of large populations and/or very long histories infeasible. One popular approach to alleviate this computational burden is to rescale forward-in-time simulations such that a smaller population is used to capture the desired evolutionary dynamics that would be expected for the larger population (Uricchio and Hernandez 2014). Typically, this is done by scaling down the population by some factor (Q) while scaling up the mutation and recombination rates by the same factor. Theoretically, this scaling approximation would preserve important population-level measures and allow easy recovery of the times at which events occurred during the simulation. For instance, the population-scaled mutation rate, θ, for a population of size N and with a mutation rate of μ, is equal to 4Nμ. After rescaling, our new population size is N/Q and our new mutation rate is μ×Q, and thus θ is unchanged:

$$\theta_{scaled}=\frac{4N}{Q}\mu Q=4N\mu$$

Meanwhile, under the standard neutral model, the expected time to the most recent common ancestor (TMRCA) between any two individuals in the unscaled simulation is 2N generations. Because the population size is decreased by a factor of Q in the scaled simulations, the expected TMRCA becomes 2N/Q. Consequently, to produce genealogies of the desired shape and size, the evolutionary model being simulated must be modified such that the time intervals between events specified in the simulated model (e.g. population splits, migration events, etc.) are divided by Q. Following the simulation, if one wishes to investigate the timescale of any stochastic events occurring during the simulation such as fixations, coalescent events between particular pairs of individuals, etc., the times of these events can simply be multiplied by Q to produce results that approximate those expected under the original unscaled model.

This population-size “rescaling trick” has been used in forward simulations for decades, with its first use dating all the way back to Hill and Robertson (1966). However, the consequences of rescaling have rarely been evaluated, and with the exception of Hill and Robertson’s initial comparison of the effects of changing N from 16 to 8, those few studies that have examined the impact of rescaling have noticed a significant effect. For example, although the approximations underlying rescaling appear to be sound at least for simulations lacking natural selection, Adrion et al. (2020) found that in some cases, rescaling by modest values of Q can bias the outcomes of purely neutral simulations, resulting in downwardly biased estimates of nucleotide diversity (π (Nei and Li 1979), Tajima’s D (Tajima 1989), and linkage disequilibrium (LD) as measured by $r^{2}$). Moreover, when natural selection is included, rescaling may introduce further biases. The selection coefficient is often rescaled by multiplying it by Q, such that Ns, a commonly used measure of the efficacy of selection, is unchanged (Uricchio and Hernandez 2014; Johri et al. 2020; Schrider 2020). However, the time to fixation of a selected mutation does not scale linearly with N. Indeed, Uricchio and Hernandez (2014) showed that, under a recurrent hitchhiking model, rescaling blunts the impact of selection on diversity levels and the site frequency spectrum. Furthermore, Comeron and Kreitman (2002) showed that rescaling alters the dynamics of interference selection for weakly selected mutations, affecting important measures such as Tajima’s D. Although these studies have examined a very limited set of scenarios, their findings suggest that undesirable effects of rescaling could be commonplace. This possibility, combined with the increasing importance and accessibility of forward simulations, underscores the need to investigate the impact of rescaling across a larger array of simulated models. We still know little about the manner in which simulations are affected (i.e. which simulation outcomes are upwardly or downwardly biased by rescaling), how widespread these effects are across simulated models, and how the severity of these effects varies with Q.

In this study, we carry out both scaled and unscaled simulations under a variety of demographic and selective scenarios and investigate the impact of rescaling on important simulation outcomes such as fixation times, site frequency spectra, and linkage disequilibrium. Our simulations explore various selection models containing different combinations of neutral, deleterious, and beneficial mutations, including simulations with a distribution of fitness effects estimated from human population genomic data. We also examine scaling effects in constant, expanding, and contracting populations. To quantify the effect of scaling, we train classification models that use a set of simulation outcomes as features to determine whether the results of scaled and unscaled simulations are distinguishable across increasing values of Q. Furthermore, for each feature we characterize the direction and magnitude of the deviation between scaled and unscaled simulations to provide a detailed picture of the effects of scaling across our scenarios. Our results show that rescaling simulations can result in a substantial deviation of numerous simulation outcomes, and that the magnitude of these effects usually increases with the value of Q. We also find that the choice of demographic model can have an impact on the severity of the rescaling effect. Overall, the results indicate the need to exercise caution in making inferences from rescaled simulations, especially when large values of Q are used.

## METHODS

### Simulation and rescaling strategy

We used SLiM 4.0 (Haller and Messer 2023: 4) and stdpopsim 0.2.0 (Adrion et al. 2020; Lauterbur et al. 2023) to carry out our scaled and unscaled simulations, all of which conform to Wright-Fisher assumptions: generations are non-overlapping, mating is random, and all individuals are monoecious, and the population size is solely determined by the specified demographic model and is not affected by the population’s fitness. For our main simulations, the mutation and recombination rates are both $Q\times 1.2\times 10^{-8}$, the genome size is 25 Mb, and the scaled population size was set to N/Q, with N=10,000. For mutations with a constant population size, a burn-in was performed for a duration of 10×N×Q generations, after which statistics were recorded during and after a simulation run of 4×N×Q generations. We also performed simulations of the full model in the presence of a single-event population expansion or contraction. In the expanding simulation, the unscaled population size expands from $N_{anc}=10,000/Q$ individuals to $N_{0}=20,000/Q$ individuals after the burn-in, while in the contraction simulations the size decreases from $N_{anc}=10,000/Q$ individuals to $N_{0}=5,000/Q$ individuals after the burn-in. In both demographic scenarios, the burn-in runs for $10\times N_{anc}$ generations, followed by $N_{0}$ generations after the population size change. For example, an unscaled simulation under the expansion scenario would run for 100,000 generations of burn-in followed by 20,000 generations, during and after which statistics summarizing the simulation results are recorded. For each simulated scenario, we generated 1000 replicates for each scaling factor (Q) except for the simulations of larger populations described below, where the computational demand necessitated a smaller sample of 100 replicates.

The fitness effects of mutations in our simulations were as specified in Table 1, and used the gamma distribution of deleterious fitness effects estimated from African American genomes by Boyko et al. 2008. Using this general structure, we performed scaled and unscaled simulations of several different scenarios each with different mixes of neutral, deleterious, and beneficial mutations (Table 2). Note that the parameter values listed in the second column of Table 2 specify the probability that any new mutation will be of a given mutation type as follows: $f_{n}$ is the probability that a new mutation will be neutral, $f_{b}$ is the probability it will be beneficial, and $f_{d}$ is the probability it will be deleterious.

We also tested the effect of scaling in several additional models as well. To test the effect of starting with a larger unscaled population size, for example, we simulated the no-beneficials model with a constant unscaled population size N=20,000. The no-beneficials model was selected since it exhibited the most dramatic effects due to scaling. We also tested a single sweep model with a population size of 100,000/Q, where a single beneficial mutation (s=0.05×Q) was introduced in the middle of the genome and then swept to fixation, whereupon the simulation was terminated. If the sweeping mutation was lost, the simulation was reset and run again until fixation occurred. Finally, we tested two *Drosophila melanogaster* constant population size models based on the stdpopsim catalog parameters for the species (Adrion et al. 2020) which specifies a population size of 1,720,600/Q (Li and Stephan 2006), a mutation rate of $5.49\times 10^{-9}\times Q$ (Schrider et al. 2013), and a recombination rate of $2.40463\times 10^{-8}\times Q$ (based on the estimate from Comeron et al. (2012)). For these simulations, we chose a 10 kb genome size and only conducted simulations at Q values of 20, 50, 100, 150, 200 due to computational limitations. For the first model, we used the distribution of fitness effects from Ragsdale et al. (2016). Since this DFE is expressed in units of $2N_{e}$ stdpopsim converts the selection coefficients to units of 𝑠 using a population size estimate of 2.8 × 10^6^ (Huber et al. 2017). This yielded a lognormal distribution with a mean of $9.679\times 10^{-7}+log(Q)$ and a standard deviation of 3.36 for the selection coefficient of deleterious mutations, and a fixed value of $7.125\times 10^{-6}\times Q$ for the selection coefficient of beneficial mutations. The second *Drosophila* model was identical to the first except that the selection coefficient for beneficial mutations was increased to 0.01×Q. All code for performing the simulations and downstream analyses in this study are available at https://github.com/SchriderLab/simscale-snakemake.

### Statistics summarizing simulation outcomes

For each simulation replicate, we recorded the following statistics summarizing the outcome:

- The site frequency spectrum (SFS) for each mutation type, each estimated from the same sample of 100 randomly selected individuals taken at the end of the simulation.
- Linkage disequilibrium (LD), as measured by the $r^{2}$ statistic calculated from the same sample of 100 individuals for all pairs of mutation or a set of 5000 randomly chosen mutations (whichever was smaller). These $r^{2}$ values were then binned by distance to produce 50 equal distance bins across the genome and the average $r^{2}$ value in each bin was recorded.
- The fixation probability for each mutation type, recorded only for mutations occurring after the burn-in. These probabilities were estimated by taking the fraction of mutations of a given type that reached fixation and rescaled by dividing this fraction by Q.
- The distribution of fixation times, recorded separately for each mutation type and only for mutations occurring after the burn-in. These times were all rescaled by multiplying by Q and binned to produce around 20–30 bins for each simulation model.

### Assessing the deviation between scaled and unscaled simulations

To assess whether scaled and unscaled simulations could be distinguished from one another for a given simulation model and a given value of Q, we trained classifiers to label a given simulation as unscaled or scaled, with the input features being the SFS, the binned data for fixation times and LD, and the fixation probabilities as described above. 80% of replicates for a given model and value of Q were used to train the classifier, and the remaining 20% of the replicates were set aside for testing. For each dataset, we trained both a logistic regression model and a random forest model. Model parameters were the default values of the Python package scikit-learn, version 1.1.3 (Pedregosa et al. 2011). The input features were grouped into the following four separate feature sets to separately test their ability to distinguish between scaled and unscaled simulations: 1) the SFS across all mutation types (199 features per mutation type) represented as a 1-dimensional vector per replicate, 2) the average $r^{2}$ values at different (binned) inter-mutation distances (50 features, all mutation types), 3) the fixation probabilities (one feature per mutation type), and 4) the histograms of fixation times (20–30 features per mutation type) concatenated along the time axis to produce a 1-dimensinoal vector per replicate. The varying number of bins was due to our goal of keeping the width of the fixation time bins fixed across all replicates, such that they map correctly to each other. Therefore, a random unscaled replicate was chosen to decide the width of the fixation time bin (in generations) to produce 20 bins, and then propagated to all replicates across all scaling factors. For instance, if the maximum fixation time for the randomly chosen unscaled replicated was 10000 generations, then the bin width would correspond to 500 generations. Since some replicates will have a different maximum fixation time, the number of bins would vary across mutation types and simulation scenarios, but each bin would correspond to the same fixation time range in all replicates and for all scaling factors. After training, classification accuracy (fraction of correct predictions on the test data) was recorded.

To further characterize the differences between scaled and unscaled simulations, the Kullback-Leibler (KL) Divergence between the scaled and unscaled fixation times and site frequency spectra were calculated. For these calculations, the distributions for each outcome were first averaged for each bin across all replicates for each scaling factor such that:

$$y_{bin}=\frac{1}{n_{replicates}}\Sigma_{n=i}^{n_{replicates}}y_{{bin}_{i}}$$

KL Divergence of the scaled distribution for each outcome was then calculated against the corresponding unscaled distribution. For LD, we opted to use the RMSE instead of KL Divergence since we summarized LD by the average $r^{2}$ values within genomic distance bins rather than probability distributions of $r^{2}$ values. This was obtained by averaging the squared error values for each bin in the LD distribution for each scaling factor such that:

$$RMSE_{LD}=\sqrt{\frac{\sum_{bin\text{=1}\;}^{50}\;\;{\left(r_{{bin}_{scaled}}^{2}\;-\;r_{{bin}_{unscaled}}^{2}\right)}^{2}}{50}}$$

Additionally, we calculated the mean percent error (MPE) in between the scaled and unscaled fixation times, mutation frequency, LD $r^{\wedge }2$ values, and fixation probabilities by first obtaining the mean of each outcome for each replicate, averaging the means across all replicates, and calculating the MPE as:

$$\text{MPE}=100\times \frac{\overset{-}{\mu_{unscaled}}\;-\;\overset{-}{\mu_{scaled}}}{\overset{-}{u_{scaled}}}$$

Where μ is the mean of the outcome for each replicate.

### Assessing the impact of sample size on estimates of deviation between scaled and unscaled simulations

To investigate the impact of the number of replicates on the estimation of deviation due to scaling, we recalculated our MPEs, KLD, and LD average RMSE values for our simulation outcomes between scaled and unscaled simulations using smaller sample sizes of 100 and 10 for the full model, the no-beneficials model, and the *Drosophila* model with stronger selection on beneficials. For each of those deviation measures, we sampled the appropriate number of replicates randomly for each scaling factor and recalculated our estimates as described above.

## RESULTS

### Rescaling alters the SFS, LD, and fixation times/probabilities in a simulated population with a gene-dense genome

To characterize the impact of population size rescaling on simulations, we simulated a variety of scenarios and examined how rescaling alters allele frequencies, LD, and the probability of and time to fixation (Methods). We began by examining simulations of a constant-sized population with a genome densely packed with selected mutations (both deleterious and beneficial, with 5% of selected mutations belonging to the latter category); because this is our richest model in terms the amount of natural selection, we refer to it as the “full model” (Methods).

**Figure 1.**
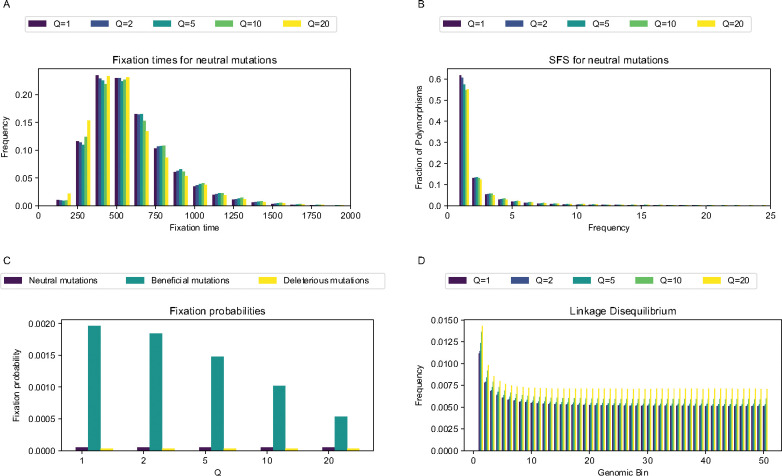
Select simulation results for the full model across scaling factors. **(A)** Fixation times for neutral mutations. **(B)** The site frequency spectra for neutral mutations. **(C)** Linkage disequilibrium across 50 genomic bins for all mutations. **(D)** Fixation probabilities for all mutations. As higher values of Q are used, all statistics show larger deviations from values observed in unscaled simulations. Fixation probabilities for beneficial mutations are impacted markedly more than for other mutation types.

When examining the distribution of mutation fixation times for neutral mutations under the full model (Figure 1A), the differences between the unscaled and scaled distributions are subtle and non-monotonic. For instance, fixation times at Q of 2 are slightly shifted towards longer sojourns, while those at Q of 20 are shifted in the opposite direction. The distributions of fixation times for beneficial and deleterious mutations (Figure S1) also show this non-monotonic trend, with longer sojourns at Q of 2, 5 and 10 and shorter sojourns at Q of 20. The site frequency spectrum for neutral mutations (Figure 1B), again shows a non-monotonic trend across values of Q. The fraction of mutations that are singletons, for which differences are most pronounced, decreases with increasing values of Q and slightly increases at Q of 20 compared to Q of 10. The SFS for deleterious mutations follows a very similar pattern to neutral mutations, while for beneficial mutations the fraction of singletons increases more dramatically at Q=20 (Figure S1). The amount of LD across the genome markedly changes with Q (Figure 1C) as it appears that rescaling increases LD between mutations regardless of the distance between them. Furthermore, the fixation probabilities for all mutation types also change with Q (Figure 1D), with the largest shift occurring for beneficial mutations; we note that this comparison was done after rescaling all fixation probability estimates themselves to account for the impact of rescaling on the neutral fixation probability (Methods). For example, for beneficial mutations, the fixation probability was around 0.20% for unscaled simulations and gradually decreased to around 0.05% for Q=20. On the other hand, the fixation probabilities for neutral and deleterious mutations remain steady at ~ 0.005% and 0.003%, respectively, for all values of Q.

**Figure 2.**
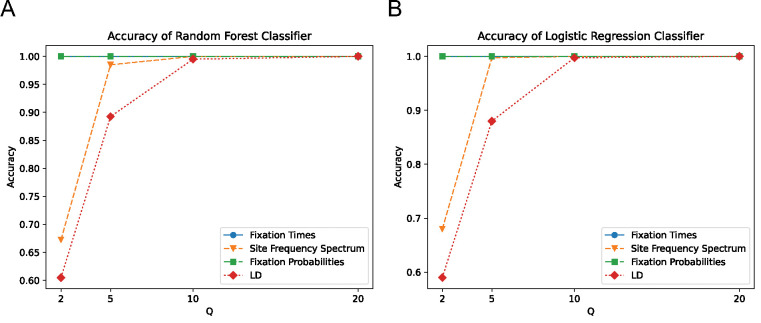
Classifier accuracy for models trained to distinguish between scaled and unscaled replicates of the full model. **(A)** Accuracy of the Random Forest classifier trained to classify a given simulation replicate as scaled or unscaled. **(B)** Accuracy of the Logistic Regression classifier trained to classify a given simulation replicate as scaled or unscaled. A separate classifier was trained for each combination of Q value and set of features (those summarizing the SFS, fixation probabilities, LD, and the distribution of fixation times). Accuracy represents fraction of simulation replicates in the test dataset correctly identified as scaled or unscaled.

Next, we trained classifiers to determine whether scaled and unscaled simulations were distinguishable from one another based on these simulation outcomes. To assess this, we trained a set of binary classifiers, each using a different set of input features (fixation times, the SFS, fixation probabilities, and LD) to discriminate between unscaled simulations at a given value of Q. As Q increases, classification accuracy increases for the SFS- and LD-based classifiers, starting at 67% for the SFS at Q=2 and increasing to 100% at Q=10 for the logistic regression classifier. For LD, the accuracy of logistic regression increases from 61% at Q=2 to 99.5% at Q=10. For fixation times and fixation probabilities, accuracy is already at 100% when Q=2 (Figure 2A), consistent with a larger deviation between scaled and unscaled simulation outcomes at higher values of Q. Similar results were obtained by the random forest classifiers (Figure 2B). Most importantly, we found that all feature sets could be used to distinguish between scaled and unscaled simulation replicates, even at relatively modest values of Q. For example, the minimum accuracy observed at Q=5 was 89% for the random forest classifier and 88% for the logistic regression classifier (both examining LD). The high accuracy obtained by classifiers discriminating between unscaled and rescaled simulations demonstrates that the rescaling effects summarized in (Figure 1) are significant and systematic.

**Figure 3.**
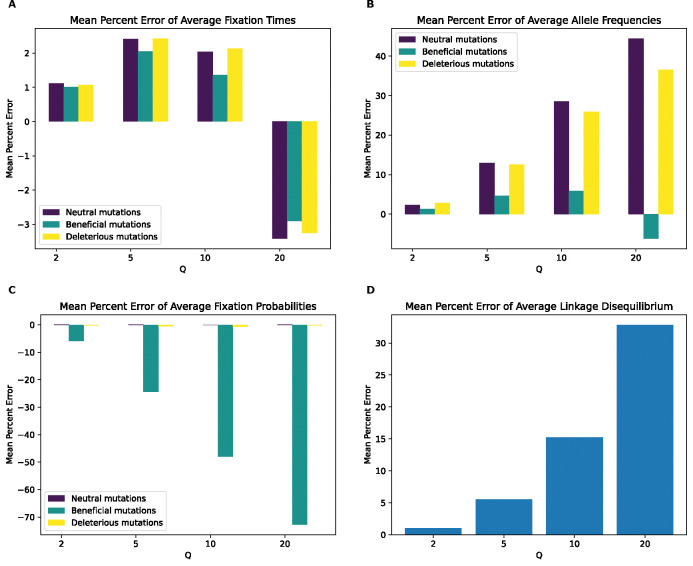
Mean percentage error of various statistics for the full model by values of Q. **(A)** Mean percentage error for average mutation fixation times. **(B)** Mean percentage error for average allele frequencies. **(C)** Mean percentage error for average fixation probabilities. **(D)** Mean percentage error for average linkage disequilibrium as measured by the values of $r^{2}$. For each statistic, the mean value was calculated for each replicate, followed by averaging the means across all replicates in each value of Q. The mean percentage error was then calculated between the resulting value from each Q and the corresponding value at Q of 1.

Having established that rescaling significantly influences simulation outcomes, we sought to quantify the magnitude of this effect. We first examined the mean percent deviation between scaled and unscaled simulations (hereafter referred to as mean percent error, or MPE) for each feature examined above (Figure 3). MPE for fixation time and allele frequency shows a non-monotonic rescaling effect across values of Q. For fixation times, MPE for all mutation types increases between Q values of 2 and 5, but decreases with Q=10, and then flips sign at Q=20 where it exhibits the largest magnitude across scaling factors. The decrease in error between Q=5 and Q=10 occurs due to an initial increase in the mean fixation time as Q increases. For example, in the case of neutral mutations the mean fixation time increases from 622 generations at Q=1 to 629 and 637 generations at Q of 2 and 5, respectively. This is followed by a decrease in mean fixation time to 634 and 600 generations at Q of 10 and 20, respectively. This causes the mean fixation time to be closer to that of the unscaled simulation at Q=10 compared to Q=5, before it decreases below the unscaled simulation mean at Q=20. When comparing the different mutation types within each Q, fixation time MPEs appear to be the lowest for beneficial mutations across all values of Q while neutral and deleterious mutations both exhibit slightly larger MPEs that are similar to one another. For allele frequencies, we observed a considerably higher MPE, which increases monotonically in magnitude from Q=2 to Q=20 for all mutation types. Here, neutral mutations have the highest MPE across Q values, reaching ~44% for Q=20 while beneficial mutations have the lowest error at −6.2% for Q=20 and are the only type of mutation to experience a sign change in MPE (occurring at Q=20). MPE was also large for LD and for the fixation probability of beneficial mutations, and again increased in magnitude with Q. However, deleterious and neutral mutations exhibit miniscule MPEs for their fixation probabilities.

As an alternative way of quantifying the degree of deviation between scaled and unscaled simulations, we examined KL divergence for fixation times and SFS distributions and RMSE for LD (Figure S2). For the distribution of fixation times, all mutation types show similar KL divergence within each scaling factor, with KL divergence increasing steadily for each mutation type as Q increases. This trend changes at Q=20, where beneficial mutations display substantially higher KL divergence than other mutation types. Similarly, when examining the SFS, KL divergence for each mutation type increases with Q, although in this case, while beneficial mutations again have the highest KL divergence at Q=20, neutral mutations have markedly higher KL divergence at Q=10. For LD decay, the root mean squared error (RMSE) between scaled and unscaled simulations a similar trend as MPE, increasing with Q from around 0.00006 at Q=2 to around 0.02 at Q=20.

### Rescaling effects persist in simulations with deleterious mutations only

Our full model serves as a useful stress test of the robustness of forward simulations to rescaling but contains a larger number of beneficial mutations than will be realistic in most cases (5% of non-neutral mutations). To see how rescaled simulations would behave without such a pervasive impact of positive selection, we altered this model by removing all beneficial mutations (the “no-beneficials” model). Under this model, we again observe a significant scaling effect, as our classifiers were able to discriminate between scaled and unscaled simulations with higher accuracy as obtained for the full model (Figure S4). One notable exception to this was the random forest using fixation probabilities at Q of 2, where accuracy was ~50%, no better than random guessing.

**Figure 4.**
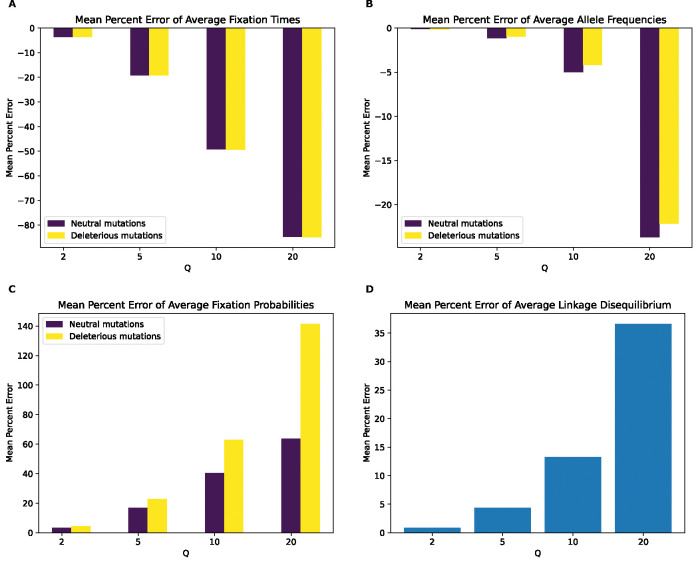
Mean percentage error of various statistics for the model with deleterious mutations only by values of Q. **(A)** Mean percentage error for average mutation fixation times. **(B)** Mean percentage error for average allele frequencies. **(C)** Mean percentage error for fixation probabilities. **(D)** Mean percentage error for linkage disequilibrium as measured by the values of $r^{2}$.

We next examined the magnitude of the rescaling effect for the no-beneficials model as measured by MPE. We observed much larger MPEs for fixation times for the no-beneficials model (Figure 4) than for the full model for fixation times, LD, and fixation probabilities. However, we observed smaller MPEs for allele frequencies than under the full model (MPE is approximately 5% at Q=10 compared to 28% for neutral mutations in the full model and increase to over 20% at Q=20 compared to 44% for neutral mutations in the full model), with both mutation types having similar errors across values of Q. Of note here is that unlike our full model, the magnitude of error increases monotonically and in the same direction for all measured statistics, with fixation times and allele frequencies exhibiting negative MPE. This indicates that rescaling in this model lowers average fixation times and allele frequencies, rather than increasing them as was the case for most values of Q under the full model. LD and fixation probabilities, on the other hand, exhibit positive MPE, with the MPE for LD increasing with Q in a similar manner to the full model, with slightly smaller values at all values of Q except 20, where the error is around 37% compared to the full model’s 33%. The MPE for fixation probabilities was quite high, especially for deleterious mutations, with values peaking at around 142% for deleterious mutations and 64% for neutral mutations at Q=20. Interestingly, under the full model, the corresponding MPEs for these mutation types were negligible, as that model instead showed large MPEs for beneficial mutations, suggesting a substantial change in the dynamics of rescaling effects depending on the presence or absence of large numbers of beneficial mutations.

KL divergence for the fixation time distribution in the no-beneficials simulations (Figure S4) again increases monotonically with Q, with similar values for both mutation types, but shows a much greater magnitude than observed for the full model across Q values. For the SFS, KL divergence is lower than that of the full model for lower values of Q, but suddenly increases to higher values than observed under the full model at Q=20. Consistent with the full model, however, neutral mutations show larger KL divergence than deleterious ones. For LD, KL divergence increases monotonically with Q and exhibits higher values at all values of Q compared to the full model. Overall, the comparison between the full model and no-beneficials model suggests that the presence of large amounts of positive selection may, at least in some cases, actually diminish the impact of rescaling on simulation outcomes.

**Figure 5.**
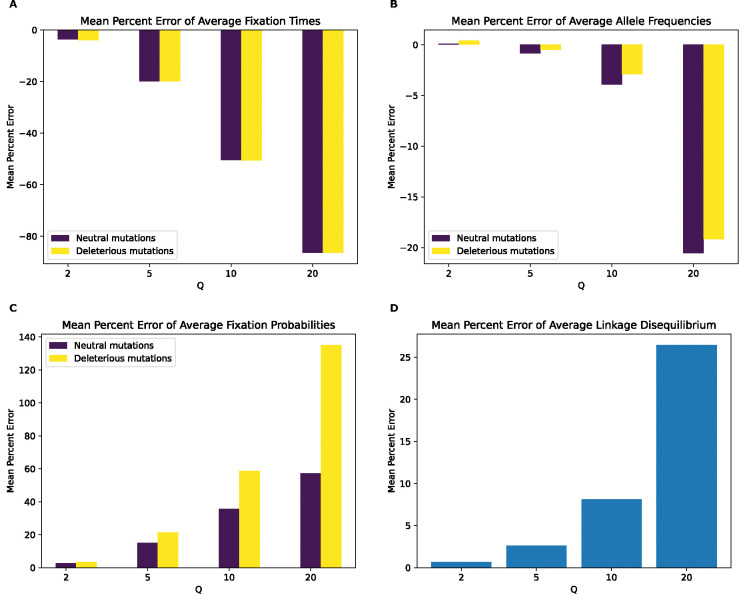
Mean percentage error of various statistics for the model with deleterious mutations only but a larger starting population by values of Q. **(A)** Mean percentage error for average mutation fixation times. **(B)** Mean percentage error for average allele frequencies. **(C)** Mean percentage error for average fixation probabilities. **(D)** Mean percentage error for linkage disequilibrium as measured by the values of $r^{2}$.

Given the large scaling effects present in this model, we sought to test the impact of increasing the population size on these effects. To this end, we carried out simulations of this same model containing only deleterious and neutral mutations but with double the starting population size (N=20000). Results indicate that MPE for fixation times (Figure 5A) and fixation probabilities (Figure 5C) are nearly identical to their counterparts in a smaller population (compare to Figure 4). MPE for LD and the SFS are slightly diminished (Figure 5D and B), on the other hand, compared to their counterparts in the smaller starting population model. We note that this change is not uniform across all values of Q. For instance, increasing the unscaled population size decreases the MPE for allele frequency by around ~3% at Q=20 for both neutral and deleterious mutations, but only by ~1% at Q=10. For LD, increasing the population size reduces the MPE by ~10% at Q=20 and by ~5% for Q=10.

### Sparse genome model

Next, we examined a model with a much smaller fraction of deleterious mutations (5% of all mutations), as might be expected in a large genome where functional elements are sparse. As in the previous section, we did not include beneficial mutations. Under this model, our classifiers were not as effective at discriminating between unscaled and rescaled simulations at lower values of Q (Figure S8), especially for fixation times and fixation probabilities where the random forest model accuracy is approximately 53% for both features at Q=2 and 76% and 69% respectively at Q=5. However, we note that at Q=20 our classifiers were still quite accurate, reaching 100% on all feature sets for both classifiers. Thus, while simulation outcomes are not as impacted at lower Q values compared to the full model or a denser genome lacking beneficial mutations, they still appear to be significantly affected by rescaling under our sparse genome model, particularly at higher Q values.

Concordant with our decreased classifier accuracy, the MPE of fixation times for this model is lower across all statistics than that of the dense model without beneficial mutations (Figure 5). Again, the MPE for allele frequencies, fixation times and probabilities, and LD increases monotonically with Q. However, one notable difference is that the direction of the bias caused by rescaling, as indicated by the sign of the MPE, is reversed for allele frequencies compared to the dense model with deleterious mutations only—here we typically observe a slight increase in the average frequencies of neutral and deleterious mutations, as was observed under the full model including beneficial mutations rather than the dense genome model with deleterious mutations only. However, we again stress that the magnitude of this bias is much smaller under this sparse genome model than either of the two dense genome models (MPE <1% for allele frequencies at Q=20). The MPE is still appreciable for the other simulation outcomes at Q=20, however (~8% for fixation times, ~17.5% for LD, and ~35% for fixation probabilities).

KL Divergence for fixation times and the SFS (Figure S8) reveals that for simulations under the sparse model, the distributions of outcomes for deleterious mutations are generally more impacted by rescaling than are neutral mutations, except for fixation times at Q=20 where the divergence is similar for both mutation types. When compared with the dense-deleterious model and, consistent with the smaller MPE values observed in this sparse model, KL Divergence for fixation times and the SFS are also smaller under the sparse model, with KL divergence for fixation times declining from ~14 for the dense-deleterious model for both mutation times at Q of 20 to ~0.04 at this Q for the sparse model. SFS KL divergence for neutral and deleterious mutations is around 0.0003 and 0.0005 respectively at Q of 20, compared to 0.046 and 0.034 for the corresponding values in the dense-deleterious model. Also consistent with the MPE trends for the sparse model is the observation that KL divergence for fixation times is similar for both mutation types and is higher for deleterious mutations in the case of the SFS. RMSE for LD is also lower compared to the dense-deleterious model, measured at 0.001 at Q of 20 compared to the corresponding dense-deleterious value of 0.002.

### Strictly neutral model

**Figure 6.**
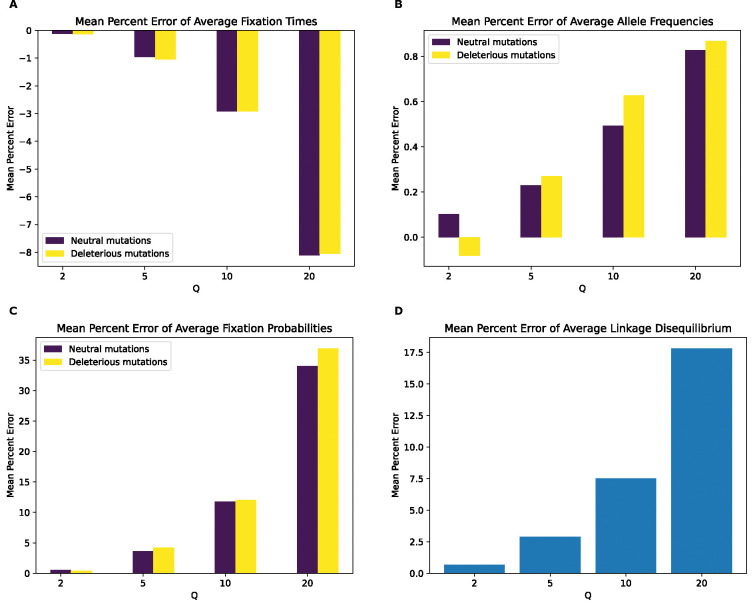
Mean percentage error of various statistics for the sparse genome model by values of Q. **(A)** Mean percentage error for average mutation fixation times. **(B)** Mean percentage error for average allele frequencies. **(C)** Mean percentage error for average fixation probabilities. **(D)** Mean percentage error for average linkage disequilibrium as measured by the values of $r^{2}$.

Next, to investigate the degree to which natural selection (whether linked or direct) is responsible for the rescaling effects described above, we performed simulations identical to the previous models but with neutral mutations only. In this case, classification of simulations as scaled or unscaled becomes more challenging for both models (Figure S10) and fixation times and probabilities are uninformative features for all values of Q. However, some impacts of scaling on the SFS and LD appear to persist, as the random forest model achieves an accuracy of approximately 90% and 100% on those features, respectively, at Q=20. This indicates that rescaling can still impact allele frequencies and LD even in the absence of selection, as previously shown by Adrion et al. (2020). These results are corroborated by the MPE values for these statistics (Figure 6) which show very small values for fixation times and probabilities but larger values for allele frequencies and LD. For LD, the MPE at Q=20 is around 16% under the strictly neutral model, which is comparable to that of the full model at Q=10, while the allele frequency MPE at Q=20 is around 1.1% under the strictly neutral model which is smaller than the MPE for neutral allele frequencies under the full model (the MPE for the neutral model at Q=20 is comparable to that of the full model at Q=2). Fixation times and SFS KL divergence and LD RMSE values for this model (Figure S10) are also smaller than for the full model, but the LD RMSE is the closest with an RMSE of around 0.001 at Q=20 under the strictly neutral model, compared to 0.002 for the corresponding value in the full model.

### The rescaling effect is visible in both expanding and contracting populations

Researchers are often interested in observing evolutionary dynamics in populations experiencing demographic changes. We therefore next sought to examine the effect of population size change on the rescaling effect. First, we simulated a variant of our full model where the population experienced a recent two-fold expansion (Methods). Our classifiers for distinguishing between rescaled and unscaled simulations performed similarly to those for the full model with a constant-size population (Figure S12), although accuracy was generally slightly lower under the expansion model for LD features across values of Q below 20. MPEs for fixation times (Figure 8A) for all mutations are higher across all values of Q except for 20, where the MPE becomes smaller in magnitude compared to the constant sized model. For instance, the MPE for neutral mutations at Q values of 2, 5, and 10 were around 1.3%, 3%, and 3.8% respectively for this model compared to around 1.1%, 2.4%, and 2% respectively for the constant-size full model. At Q of 20 however, the error here decreases to 1.6% compared to the constant-size model’s −3.4% (note the sign change, which does not occur under the expansion model). The MPEs for all other statistics (Figure 8B–D) are very similar to those under the constant-size model but are smaller for LD across all Q values. KL divergence values (Figure S12) for fixation times are lower in this model compared to the constant-size model, with beneficial mutations having the lowest KL divergence at Q=10 and Q=20 in contrast to the constant-size model (where beneficial mutations exhibited the largest KL divergence at these Q values). For the SFS KL divergence here is very similar to the constant-size model, while the LD RMSE values are lower across all values of Q. Overall, simulations of a population experience a recent expansion seem to produce a rescaling effect that is fairly similar to that of a simulated constant-size population, with the biggest observed difference being in the magnitude of divergence for LD and the magnitude and direction of divergence for fixation times at Q of 20.

Our next exploration of the effect of population size change was the simulation of a two-fold *decrease* in population size. Again, our classifiers for this model perform similarly to those for the constant-size model, although the classifiers examining LD were slightly more accurate (Figure S14). Comparing MPE between the contraction and constant-size models, we observe fairly similar results for allele frequencies, fixation probabilities, and LD (Figure 9). However, for fixation times, we observe smaller MPE values for Q=2 through Q=10, and a sign change from positive to negative MPE occurs at Q=10 whereas this is not observed until Q=20 under the constant-size model. More notably, the magnitude of MPE for fixation times suddenly increases to values much larger than those observed under the constant-size model (~8–10%, depending on the mutation type, for the contraction model vs. ~3% for the constant-size model). Concordantly, fixation times show similar KL divergence under the contraction model (Figure S14) compared to the constant-size model except for Q=20 where KL divergence increases markedly compared to the constant-size model, with values of 0.057, 0.041, and 0.057 for neutral, beneficial, and deleterious mutations at Q=20 compared to the constant-size model’s values of 0.015, 0.022, and 0.016 respectively. Beneficial mutations are also no longer showing the highest KL divergence for fixation time across any Q. The KL divergence for the SFS is very similar, with slightly higher KL divergence for beneficial mutations especially at Q=20, where the KL divergence is around 0.35 compared to the constant-size model’s 0.03. For LD, RMSE values are all higher than the constant-size model, with a value of 0.0027 at Q=20 compared to the constant-size model’s 0.002.

### Scaling effects are greatly diminished in a larger simulated population modeled after *Drosophila* populations

Next, to investigate the impact of rescaling in a larger population, we performed simulations of a *Drosophila melanogaster* population (Methods). When applying the classifiers to this model, we observe the worst performance across all values of Q than seen for any of the other simulation models examined above (Figure S16). While accuracy hovers around 50% (i.e. random guessing) for all features across almost all Q values, the random forest classifier using the SFS can achieve 63% accuracy at Q=20, suggesting that the SFS is the simulation outcome most affected by rescaling in this model. Note that the lower accuracy here may be in part due to the smaller chromosomes we simulated for this model because of its greater computational burden (Methods). Thus, there are fewer mutations within each replicate, providing less information to the classifiers. However, this would not affect KL divergence and MPE as much since these measures aggregate information across replicates (although those metrics could still be inflated by noise in sparse datasets; see below).

When examining MPE (Figure S16) and KL divergence (Figure S17) we observe substantial bias in allele frequencies and fixation times and probabilities for deleterious mutations, but comparatively subtle effects observed for neutral and beneficial mutations. For instance, the average fixation time MPE is ~70% at Q=200 for deleterious mutations, but only around −0.7% and −0.1% for neutral and beneficial mutations, respectively. The MPE for the average fixation probability is also very high for deleterious mutations at Q=200 (~200%) and low for beneficial and neutral mutations (~−0.6% and ~1.5% respectively). While the MPE for fixation probability is higher at Q=200 for deleterious mutations, it is not monotonic for neutral and beneficial mutations, since the highest fixation time MPE in magnitude is at Q=150 for neutral mutations (~−0.84%) and at Q=50 for beneficial mutations (~0.36%) and the highest fixation probability MPE is at Q=50 for beneficial mutations (~2.5%). While the fixation probability MPE is at its highest at Q=200 for neutral mutations, it only increases in magnitude monotonically, but abruptly flips sign at Q=100. For allele frequency MPE, the trend is non-monotonic for all three mutation types and is higher at Q=150 for deleterious mutations (~17%), at Q=100 for beneficial mutations (~−2%) and at Q=50 for neutral mutations (~−0.8%). However, it is important to note here that the probability of the fixation of deleterious mutations is extremely small, ~1.5 × 10^−12^ for Q=20 and no such fixations were observed for Q values of 50, 100, and 150 (yielding a fixation probability estimate of zero). This also means that the distribution of fixation times for deleterious mutations was based on very sparse information and in some cases could not be estimated at all. This was not a concern for neutral and beneficial mutations, however, and we observed that KL divergence for fixation times of these mutations (Figure S16) shows a weak and non-monotonic trend with increasing Q. KL divergence for the SFS and RMSE for the LD were also small and non-monotonic. Overall, the degree of the rescaling effects under this model was fairly small for beneficial and neutral mutations. Although our error measures were much higher for deleterious mutations, these probably reflect the sparsity of our data rather than a genuinely strong rescaling effect.

### Scaling effects are substantial in a population modeled after *Drosophila* but with stronger selection on beneficial mutations

In the previous section we examined the behavior of a *D. melanogaster* population model with weak selection acting on beneficial mutations ($s=7.125\times 10^{-6}$ (Ragsdale et al. 2016; Lauterbur et al. 2023)). We therefore sought to examine whether the scaling effects remain diminished in our *Drosophila* modeled population when a higher selection coefficient for beneficial mutations is used (s=0.01); we refer to this model as the “strong beneficial” model. Classifier results (Figure S19) indicate that scaling effects are much more drastic when strongly beneficial mutations are present. Accuracy for the random forest model for fixation times and probabilities are 100% for all values of Q. Accuracy for LD and SFS features are close to 50% for Q of 50 but increase to around 63% and 66% respectively for Q of 100 and increase to around 69% and 75% respectively at Q of 200.

MPE of fixation times (Figure 10A) shows a monotonic increase in error values for all mutation types with increasing Q. While deleterious mutations have errors that are smaller and in the opposite direction than the model with weaker beneficial mutations across all values of Q, errors for beneficial and neutral mutations are much larger. For instance, neutral mutations exhibit an MPE for average fixation time of ~12% at Q=50 which increases to 70% for Q=200. In contrast the fixation time MPEs for neutral mutations under the weak beneficial model at those same values of Q are −0.4% and −0.6%. MPE for beneficial mutations’ average fixation time in the strong beneficial model is ~13% at Q=50 and increasing to around 70% at Q=200, compared to the corresponding values of 0.4% and −0.09% in the weak beneficial model. Deleterious mutations exhibit errors of ~10% at Q=1 and 54% at Q=20. We note that unlike under the weak beneficial model, deleterious mutations do occasionally fix at all values of Q under the strong beneficial model, likely due to hitchhiking with beneficial mutations.

The trend for mutation frequency MPE (Figure 10B) is more complex and non-monotonic. For neutral mutations, MPE alternates its sign at each successive Q, increasing in magnitude from ~−0.2% at Q of 50 to ~13% for Q of 200. Deleterious mutations exhibit the highest error at Q of 50 (around 48%) which then declines at Q of 100, increases again at Q of 150, and then decreases to its lowest value of ~14% at Q of 200. In contrast, beneficial mutation frequency MPE increases monotonically from ~4% at Q of 50 to ~14% at Q of 200. Of note here is that while all mutation types have drastically different values of MPE for allele frequency at Q=50,100,150, they eventually exhibit similar errors at Q of 200. Furthermore, all error values for this model are higher compared to their corresponding mutations type in the weak beneficial model, even for deleterious mutations which have a maximum error of around 17% for the weak beneficial model. For fixation probabilities (Figure 10C), MPE is lowest for neutral mutations, where the highest magnitude MPE is at Q of 50 (around −0.5%) which flips sign at Q of 100 to 0.11% and increases to around 0.4% at Qs of 150 and 200. Beneficial and deleterious mutations have similar errors, which are negative and increase in magnitude monotonically with Q to −0.30% for both mutation types. This starkly different than the corresponding values for the lower selection *Drosophila* model, where neutral and beneficial mutations have maximum errors of 1.2% and 2.5%, respectively and again where deleterious mutations fixed very rarely, preventing the reliable estimation of MPEs. MPE values for LD (Figure 12C) are also non-monotonic with Q as was the case for the weak beneficial model, but are much higher in magnitude, with the highest value being around 4.7% at Q of 100, compared to the highest value of around 0.34% at Q of 200 for the lower selection model.

KL divergence for fixation times (Figure S19) increases monotonically with Q from values of around 0.05, 0.16, and 0.08 for neutral, deleterious, and beneficial mutations respectively at Q of 50 to around 1.09, 1.7, and 1.51, respectively, at Q of 200. On the other hand, KL divergence values for the SFS exhibit different trends for each mutation type; for beneficial mutations, KL divergence increase monotonically from around 0.02 at Q=50 to around 0.06 at Q=200. For neutral mutations, the trend is actually a monotonic decrease from a value of around 0.3 at Q=50 to around 0.21 at Q of 200. KL divergence for deleterious mutations is non monotonic, initially increasing from a value of about 0.53 at Q=50 to around 0.6 at Q=100, decreasing 0.52 and 0.54 and the two subsequent Q values, respectively. RMSE for LD also does not show a clear trend, with all values hovering around 0.005.

### Scaling effects are present in a single selective sweep model

Finally, we sought to exam the effects of scaling in a simple hitchhiking model with only neutral mutations and a single beneficial mutation sweeping through the population. We focus here on the distribution of fixation times for the beneficial mutation across all replicates, and on the SFS and LD for neutral mutations. Classifier accuracy results (Figure S21) show that scaling also affects these simulations with the intensity of the effects increasing with Q. While classifier accuracy is low at Q=2, with the best accuracy being 56% for LD, accuracy increases with Q, especially for LD where accuracy is at 85% for Q=5 and reaches 100% for Q=20. The highest classification accuracy for fixation times and the SFS are also observed at Q=20 (80% and 91%, respectively). MPE for fixation times of the beneficial mutation (Figure 11A) are substantial and increase monotonically with Q from 1.8% at Q=2 to 35% at Q=20. MPEs for the neutral mutation allele frequency also increase with Q but are smaller, reaching only as high as 1.2% at Q=20. For LD, we also observe a monotonic increase in MPE from 0.5% at Q=2 to 16% at Q=20. KL divergence for the distribution of fixation times (Figure S21) of the beneficial mutation decreases from 0.05 at Q=2 to 0.03 at Q=5 and then increases monotonically to 0.31 at Q=20. For neutral mutations, the SFS KL divergence and LD RMSE for both increase monotonically with Q, with the highest KL divergence and RMSE values being 0.0005 and 0.001 respectively at Q=20. These results demonstrate that even with a scenario involving a single sweep, scaling can bias simulation outcomes, especially fixation times for the sweeping mutation and LD between pairs of linked neutral mutations.

### Scaling effects can be often recapitulated from a small number of replicates

Although some general conclusions can be drawn from our experiments above (e.g. rescaling effects are common and generally increase with Q), behavior differs substantially across models and thus it may be difficult for a researcher to know what the impact of rescaling may be on their simulations without conducting similar experiments themselves on the model of interest. Since it is often unfeasible to acquire many replicates of minimally-scaled (ideally unscaled) simulations under some models due to computational limitations, we sought to investigate whether statistical measures of the impact of scaling such as MPE, KL divergence, and RMSE can be reasonably approximated with a smaller number of simulation replicates for a subset of our simulations. We therefore randomly sampled smaller sets of replicates for the full model, the no-beneficials model, and the *Drosophila* model with strong beneficials for each value of Q. Our results indicate that when information about the outcome of interest is sufficiently dense in each replicate, measures of deviations gleaned from a smaller number of replicates are generally close to those obtained using all 1000 replicates. For example, in a sample of 100 replicates simulated under the full model (Figure S22), all measures of deviation are extremely similar to those obtained using 1000 replicates, with the exception of KL divergence for the SFS of beneficial mutations, which is lower for all values of Q compared to the results from 1000 replicates. When using only 10 replicates (Figure S23), deviation outcomes for the full model are still informative but less well preserved. For instance, the magnitude of the MPE of average fixation times is slightly higher for neutral and beneficial mutations at Q=5 and slightly lower at Q=20. KL divergence for fixation times and the SFS is more severely affected, especially for neutral and deleterious mutations, whereas the average RMSE for LD is still well-preserved.

For the no-beneficials model, using 100 replicates (Figure S24) preserves all measures of deviation rather well, with the exception of a lower KL divergence for fixation times of neutral mutations at Q=20 compared to that estimated from 1000 replicates. Using 10 replicates only was sufficient to recover similar estimates of MPE for average fixation times, probabilities, and LD, but induced a more marked change in the average allele frequency MPE (Figure S25). Changes in KL divergence for fixation times and the SFS are also more pronounced for this small number of replicates, with lower estimated KL divergence for both mutation types for both outcomes. The average RMSE for LD, however, remains well-preserved.

Unfortunately, down-sampling the number of replicates for the *Drosophila* model does not recapitulate the measured errors for some outcomes as well. While down-sampling to 100 replicates (Figure S26) does conserve some error measurements, such as the average fixation time MPEs for neutral and beneficial mutations, the average fixation probability MPE for beneficial mutations, and the average allele frequency for beneficial mutations, we observed substantial shifts for all other measurements, including KL divergence for fixation times and the SFS for all mutation types and the average LD RMSE. Further down-sampling to 10 replicates (Figure S27) causes further loss of ability to capture all measures of error. This result is not unexpected, as we simulated much smaller regions in the *Drosophila* genome to examine smaller scaling factors (Methods), and this resulted in a much smaller number of mutations available for analysis (e.g. the estimated SFS was based on fewer than 20 mutations present in a sample for the *Drosophila* higher selection model on average).

In summary, using a smaller number of replicates can be informative for characterizing deviation between scaled and unscaled simulation when replicates are sufficiently information dense (e.g. there are enough polymorphisms in the sample taken at the end of the simulation to estimate the SFS, or there were enough fixations during the simulation to accurately estimate the fixation probability). Using a much smaller number of replicates can impair these estimates, especially for KL divergence, and most severely for simulation models whose replicates contain sparse information such as a low number of segregating or fixed polymorphisms (e.g. our simulations of 10 kb regions in the *Drosophila* genome).

## DISCUSSION

Population genetic simulation software is increasingly powerful and flexible (Kelleher et al. 2016; Baumdicker et al. 2022). This is especially so for forward-in-time simulators that allow for complex scenarios involving different types of natural selection (Thornton 2014; Haller and Messer 2017) departures from Wright-Fisher assumptions (Haller and Messer 2019), the modeling of multispecies dynamics (Haller and Messer 2023), and more. Unfortunately, because forward simulations require the entire population to be modeled, they can be too computationally costly when the population size is large. Rescaling the simulation parameters to simulate a smaller population to model a larger one is therefore necessary in many cases. Unfortunately, it has been shown that this rescaling trick can bias simulation outcomes (Uricchio and Hernandez 2014; Adrion et al. 2020). However, the rescaling effect has only been examined for a small number of simulation scenarios, and thus we still do not know the manner and magnitude with which it will affect simulation results.

Here, we sought to investigate the rescaling effect on a wider array of simulation models, including those of varying genome densities, population sizes, and strengths of positive and negative selection. We used stdpopsim and SLiM to generate these simulations under varying values of the rescaling factor Q, and record a set of simulation outcomes summarizing the SFS, LD, and the probability of and time to fixation. Ideally, the rescaling strategy would produce results that are difficult to distinguish from unscaled simulations. However, this was not the case—for most of the models that we examined, binary classifiers were able to discriminate between rescaled and unscaled simulations quite easily. For example, for our full model, classifiers examining either the estimated fixation probability and the distribution of fixation times could label simulation replicates as unscaled or rescaled by a factor of only Q=2 with 100% accuracy (Figure 2). Classifiers using the SFS or LD also performed well under this model with only a modest degree of rescaling (e.g. nearly 100% accuracy at Q=5). Our classifiers generally performed well for the other models examined here, but there were two notable exceptions: 1) for the neutral model, only the LD-based classifier could discriminate between unscaled and scaled simulations with 100% accuracy at Q=20 (the highest rescaling factor examined for this model; see Figure S10); 2) for the *Drosophila* model with weaker selection on beneficial mutations, none of the classifiers performed well at any value of Q examined (although this was not the case for the *Drosophila* model with strong beneficials).

Even if rescaling alters simulations sufficiently to be detected by classifiers, the size of this rescaling effect may still in some cases be small enough to be tolerable by researchers. We therefore sought to more directly quantify the magnitude by measuring the mean percent error between scaled and unscaled simulations for each of our simulation outcomes. We found that MPE between scaled and unscaled outcomes was often substantial. For example, the fixation probability of deleterious mutations under our no-beneficials model is overestimated by ~140% when Q=20 (Figure 4C). However, MPE did vary considerably across simulation outcomes and models. Consider again the MPE of fixation probability under the no-beneficials model. When we add beneficial mutations into this model (yielding the “full model”; Methods), the MPE for deleterious mutations is only 0.2% at Q=20 (Figure 3C). We note that classification accuracy did not always correspond with the observed trends in MPE. For instance, in the no-beneficials model, the random forest classifier achieves higher accuracy on the SFS features than fixation times, despite allele frequencies having an MPE that is an order of magnitude lower than that of fixation times. This is probably a result of higher variance on our fixation time estimates, resulting in more overlap between scaled and unscaled simulations despite a comparatively large shift in the mean. Thus, one should bear in mind that our classification results are indicative of the degree to which the scaled and unscaled simulations are separable—which could be the case for two distributions that have a slight difference in mean value but low variance—while our estimates of MPE and KL divergence/RMSE more directly speak to the magnitude of the rescaling effect.

Our analysis of the variety of simulation models considered here does yield some conclusions that are likely to generalize to other models. For one, we might expect the rescaling effect to be more severe in smaller populations than in larger ones. For example, the magnitude of the rescaling effect observed in our no-beneficials model (Figure 4) was reduced somewhat when we re-ran these simulations with a population that was twice as large (Figure 5). We also observed a weaker rescaling effect in the simulated *Drosophila* population, although this was in part a result of the sparsity of mutation data in these simulations which modeled smaller chromosomes. However, the rescaling effect may still be appreciable in larger populations when positive selection plays a more prominent role and when *Q* is large: the rescaling effect in the *Drosophila* model with stronger beneficial mutations is far greater than that under the model with weaker beneficials (compare Figure 10 to Figure S16).

Intuitively, it is not unexpected that the rescaling effect would be greater in magnitude in the presence of selection. For example, in the presence of positive selection, the rationale for rescaling the selection coefficient by Q is not supported by theory, as neither the time to fixation nor the fixation probability are expected to be a linear function of the population-scaled selection coefficient 2Ns (Kimura 1962; Charlesworth 2020)—our results from simple simulations of a single selective sweep underscore this (Figure 11). Similarly, Uricchio and Hernandez (2014) showed that the theoretical prediction that the impact of a sweep on a linked variation is determined by 2Ns (Wiehe and Stephan 1993) is not valid when *s* is large, which will be the case when s is multiple by Q during rescaling. It has also been shown that the degree to which neutral genetic diversity at a given site is reduced by both selection against linked (Nordborg et al. 1996) and genome-wide (Hudson and Kaplan 1995) deleterious mutations is dependent on 𝑠 and not Ns, meaning that rescaling will produce inaccurate results for such models (as pointed out by Matheson and Masel (2024)).

**Figure 7.**
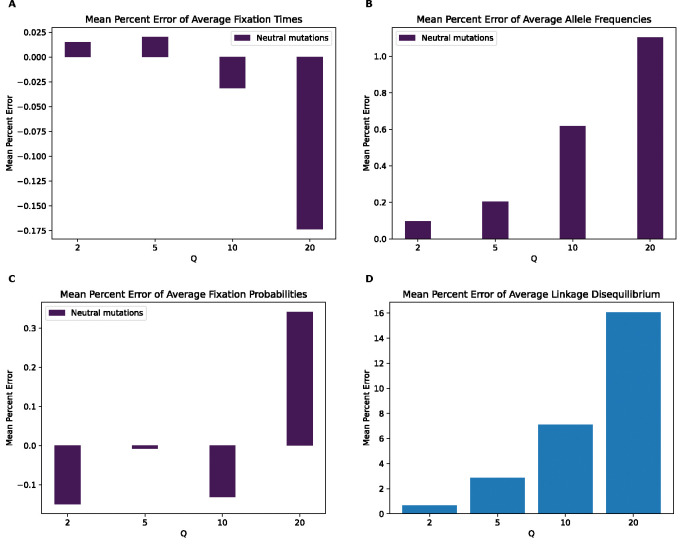
Mean percentage error of various statistics for the strictly neutral model by values of Q. **(A)** Mean percentage error for average mutation fixation times. **(B)** Mean percentage error for average allele frequencies. **(C)** Mean percentage error for average fixation probabilities. **(D)** Mean percentage error for average linkage disequilibrium as measured by the values of $r^{2}$.

**Figure 8.**
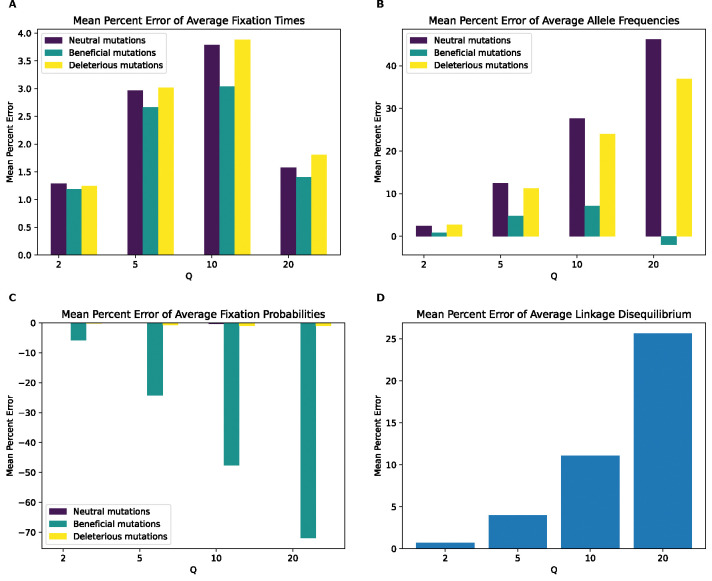
Mean percentage error of various statistics for the population expansion model by values of Q. **(A)** Mean percentage error for average mutation fixation times. **(B)** Mean percentage error for average allele frequencies. **(C)** Mean percentage error for average fixation probabilities. **(D)** Mean percentage error for average linkage disequilibrium as measured by the values of $r^{2}$.

**Figure 9.**
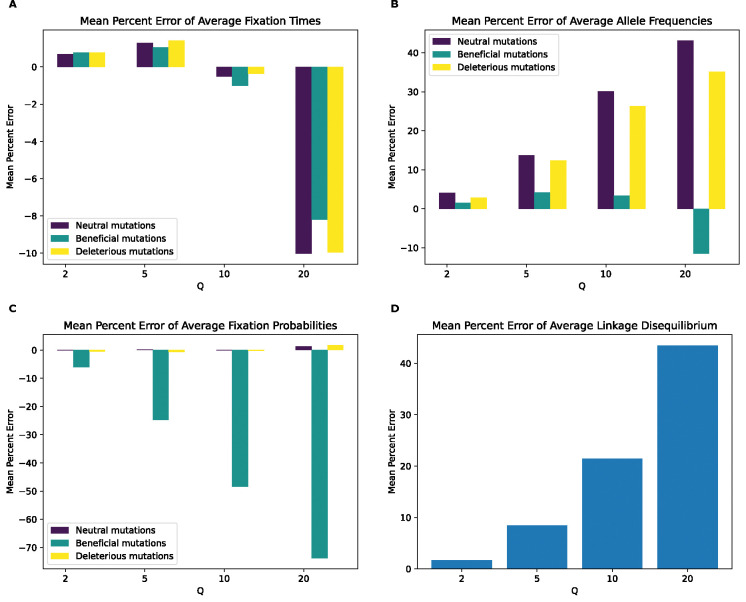
Mean percentage error of various statistics for the population contraction model by values of Q. **(A)** Mean percentage error for average mutation fixation times. **(B)** Mean percentage error for average allele frequencies. **(C)** Mean percentage error for average fixation probabilities. **(D)** Mean percentage error for average linkage disequilibrium as measured by the values of $r^{2}$.

**Figure 10.**
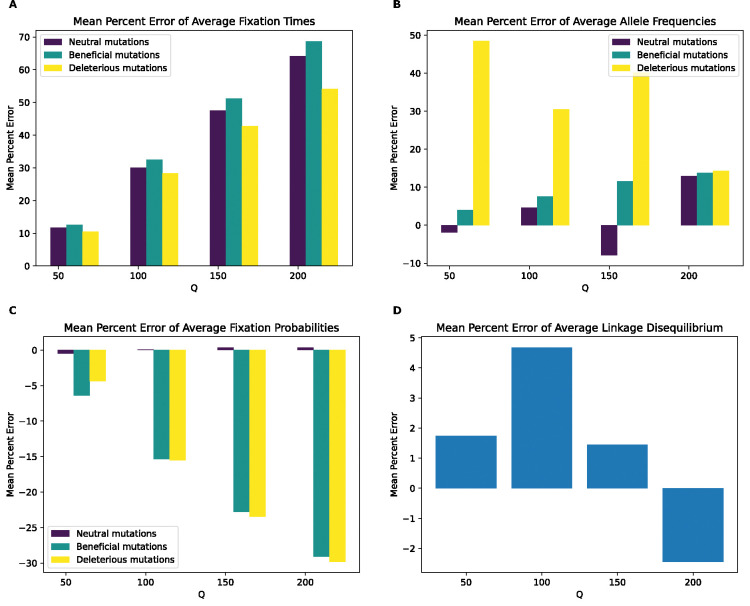
Mean percentage error of various statistics for the higher selection Drosophila model by values of Q. **(A)** Mean percentage error for average mutation fixation times. **(B)** Mean percentage error for average allele frequencies. **(C)** Mean percentage error for average fixation probabilities. **(D)** Mean percentage error for average linkage disequilibrium as measured by the values of $r^{2}$.

**Figure 11.**
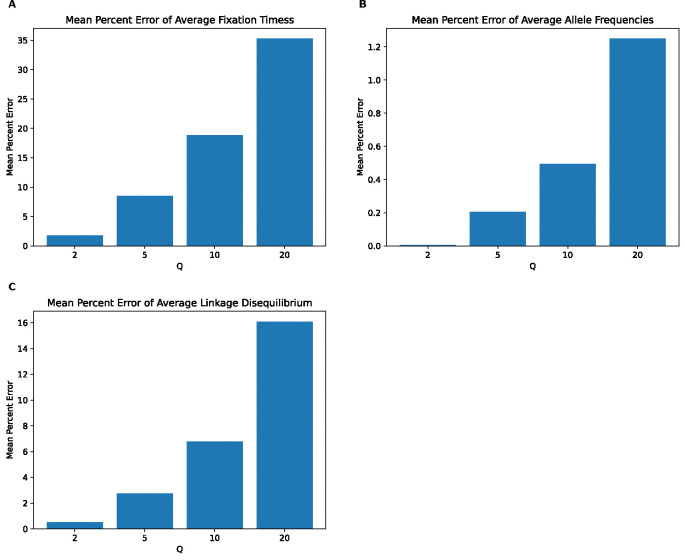
Mean percentage error of various statistics for the single sweep model by values of Q. **(A)** Mean percentage error for average beneficial mutation fixation times. **(B)** Mean percentage error for average allele frequencies for neutral mutations. **(C)** Mean percentage error for average linkage disequilibrium for neutral mutations as measured by the values of $r^{2}$.

Moreover, we would expect there to be a stronger rescaling effect in the presence of large amounts of natural selection because rescaling will cause each simulated genome to receive a larger number of selected mutations each generation, which may alter short-term dynamics before the increased recombination rate can break up this extra linkage. Finally, rescaling divides the total number of generations by Q, thereby reducing the temporal resolution of the simulation when it comes to events that occur on short timescales. For example, all estimates of selective sweep sojourn times will necessarily be rounded up to the nearest multiple of Q, resulting in underestimates. A corollary of all these lines of reasoning is that the rescaling effect should be less severe under neutral simulations, perhaps especially those of large populations. However, although the effect is more subtle, we do observe one here (Figure 7). We note that this finding is consistent with results from Adrion et al. (2020) who observed biased estimates of several summary statistics when performing purely neutral rescaled forward simulations of the demographic model from Kamm et al. (2020).

Although we find that the consequences of population-size rescaling for forward simulations are somewhat predictable, when examining the totality of our results one cannot help but be struck by numerous exceptions to the general trends described above. Indeed, as noted in numerous places throughout the Results we did not always find that the magnitude of MPE increased monotonically with Q. Thus, it is not as of yet possible to predict a priori precisely how, for a population genetic model of interest, which simulation outcomes will be most affected by rescaling, and whether these outcomes will be upwardly or downwardly biased for a given value of Q. Thankfully, we have found that even a relatively small number of simulation replicates (e.g. 10, for the cases shown in (Figure S25, Figure S26, and Figure S27) can be enough to accurately describe the impact of rescaling both qualitatively and quantitatively (via MPE) for a given Q. Thus, a small-scale test of varying Q values may be sufficient for a researcher wishing to determine which value of Q will provide the best balance between computational efficiency and accuracy of estimated outcomes. For researchers wishing to simulate very large numbers of simulation replicates each of reasonably small chromosome lengths (e.g. for ABC inference of models involving natural selection (Johri et al. 2020)), this will mean just simulating a small number of replicates for each of a range of rescaling factors. For researchers instead hoping to generate chromosome-scale simulation replicates, it may be possible to first do a small-scale test on shorter chromosomal regions to select a value of Q before conducting the full-scale study. For simpler scenarios, more theoretically motivated algorithmic approaches to selecting an optimal Q value may be possible (Uricchio and Hernandez 2014). Thus, although population rescaling may in many cases be unavoidable for the time being, researchers may be able to mitigate the undesirable effects of rescaling by carefully considering and investigating them before performing simulations at scale. Still, we suggest that continued study of the manner in which population rescaling effects simulation results and the models affected will be necessary to ensure that the continuing advances in simulation power and flexibility are being used to produce accurate results.

## Supplementary Material

Supplement 1

## Figures and Tables

**Table 1:** Mutation parameters for simulations.

Mutation Type	Fitness Effect (s)
Neutral	0
Beneficial	0.01 × *Q*
Deleterious	~*Gamma*(−0.0294 × *Q*, 0.184)

**Table 2:** Proportions of neutral, deleterious, and beneficial mutations in each simulation model.

Simulation Model Name	Model parameter values
Full model	*f_n_* = 0.25, *f_b_* = 0.75 × 0.05, *f_d_* = 0.75 × 0.95
No-beneficials model	*f_n_* = 0.25, *f_d_* = 0.75
Sparse model	*f_n_* = 0.95, *f_d_* = 0.05
Neutral-only model	*f_n_* = 1

## Data Availability

All code for performing simulations and analyzing/visualizing their results can be found at https://github.com/SchriderLab/simscale-snakemake.
