# Supplementary material for: Population size rescaling significantly biases outcomes of forward-in-time population genetic simulations
Source: bioRxiv. 2024 Sep 3:2024.04.07.588318. Preprint. [Version 3] doi: 10.1101/2024.04.07.588318 (PMC11030438; doi:10.1101/2024.04.07.588318)
Supplement: Supplement 1 [file media-1.pdf]

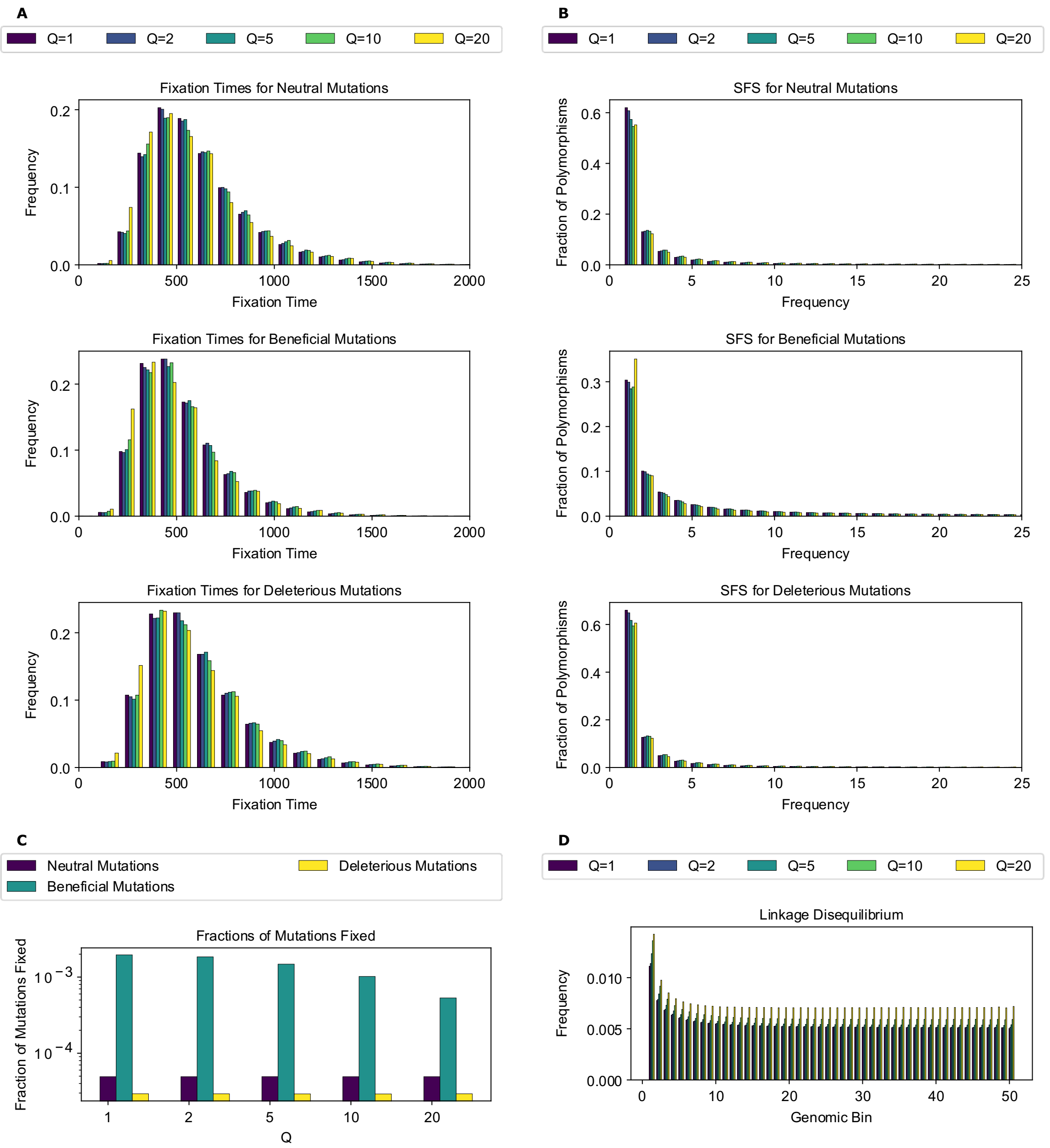

Figure S1. Simulation outcomes for the full model.

(A) Fixation time distributions for all mutation types. **(B)** Site frequency spectra for all mutation types. **(C)** Fraction of mutations fixed for all mutation types. **(D)** Linkage disequilibrium across the chromosome for 50 genomic bins, as measured by r^2. Each outcome was averaged across all replicates. For fixation times, SFS, and LD, this was achieved by averaging each distribution bin across the replicates.

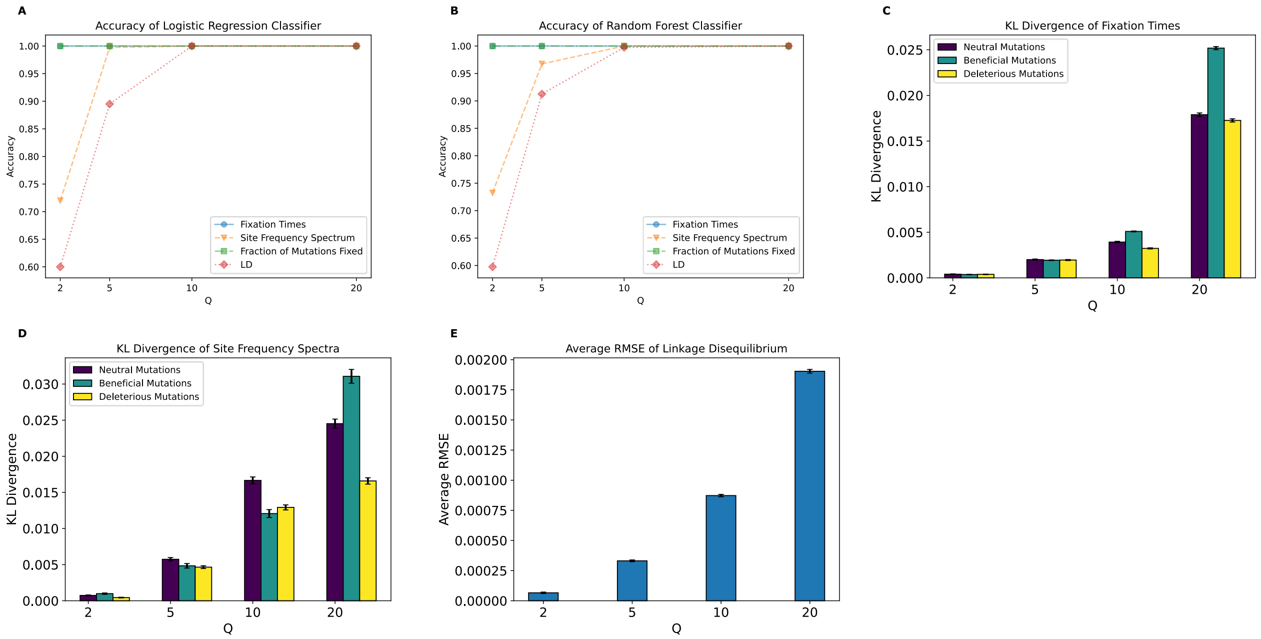

Figure S2. Metrics of the effect of scaling for the full model.
(A) Accuracy of the logistic regression classification using features subsets for each scaling factor. **(B)** Accuracy of random forest classification using feature subsets for each scaling factor. **(C)** KL divergence of fixation time distributions for all mutation types. **(D)** KL divergence for the SFS for all mutation types. (**E)** Average RMSE of LD. Classifier accuracy represents the proportion of replicates correctly identified by the model as scaled and unscaled using the feature subsets for each Q. Values and errors bars for panels **(C)**, **(D)**, and **(E)**, represent mean and standard deviation of 1000 bootstrapped samples.

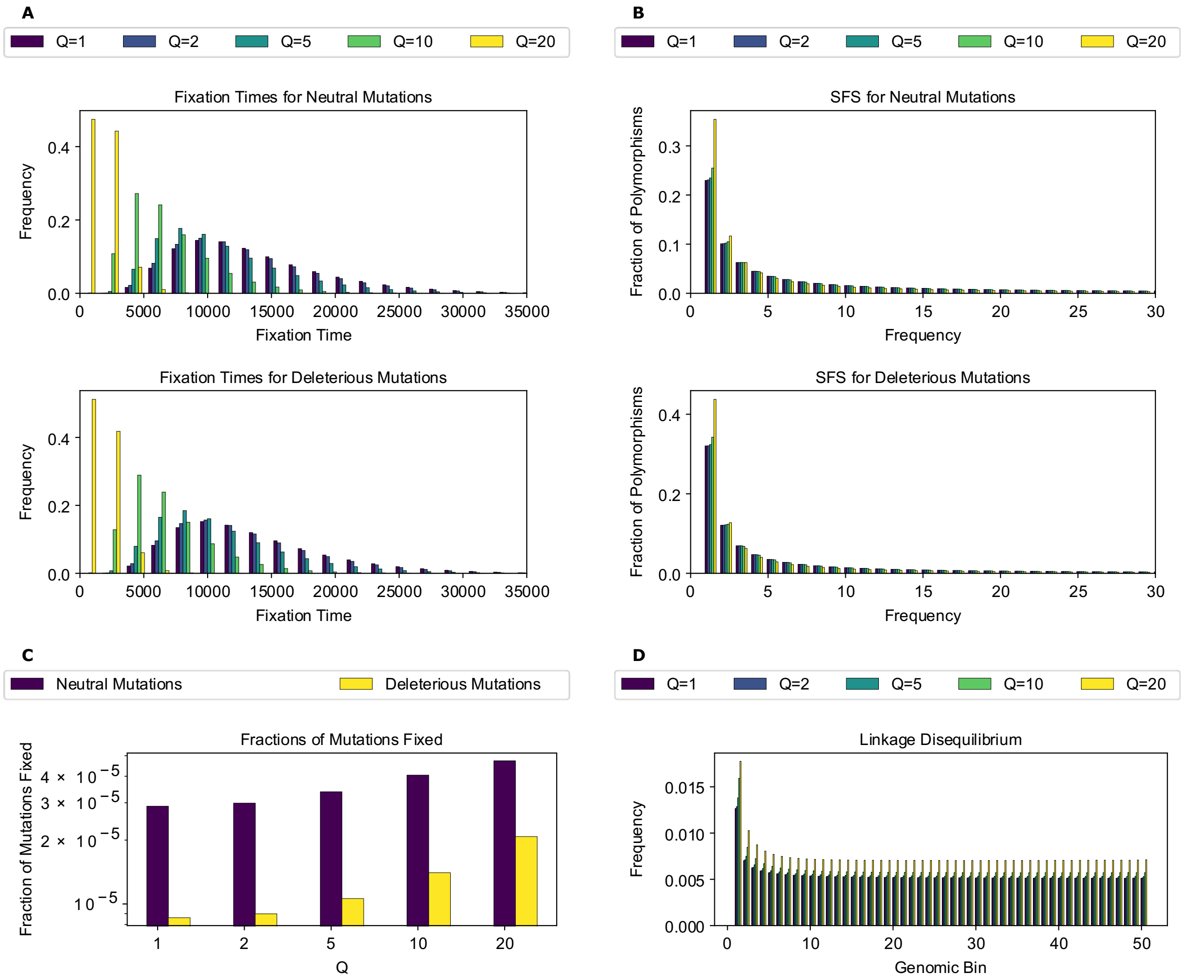

Figure S3. **Simulation outcomes for the no-beneficials model.**
(A) Fixation time distributions for all mutation types. **(B)** Site frequency spectra for all mutation types. **(C)** Fractions of mutations fixed for all mutation types. **(D)** Linkage disequilibrium across the chromosome for 50 genomic bins, as measured by r^2.

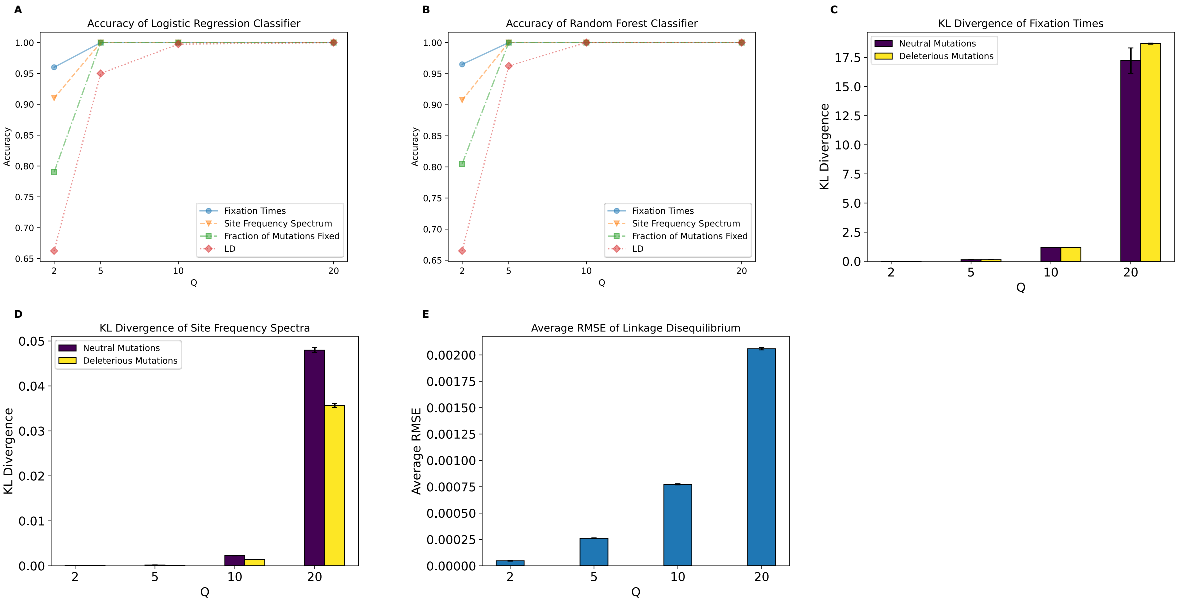

Figure S4. Metrics of the effect of scaling for the no-beneficials model.
(A) Accuracy of the logistic regression classification using features subsets for each scaling factor. **(B)** Accuracy of the random forest classification using feature subsets for each scaling factor. **(C)** KL divergence of fixation time distributions for all mutation types. **(D)** KL divergence for the SFS for all mutation types. **(E)** Average RMSE of LD. Values and errors bars for panels **(C)**, **(D)**, and **(E)**, represent mean and standard deviation of 1000 bootstrapped samples.

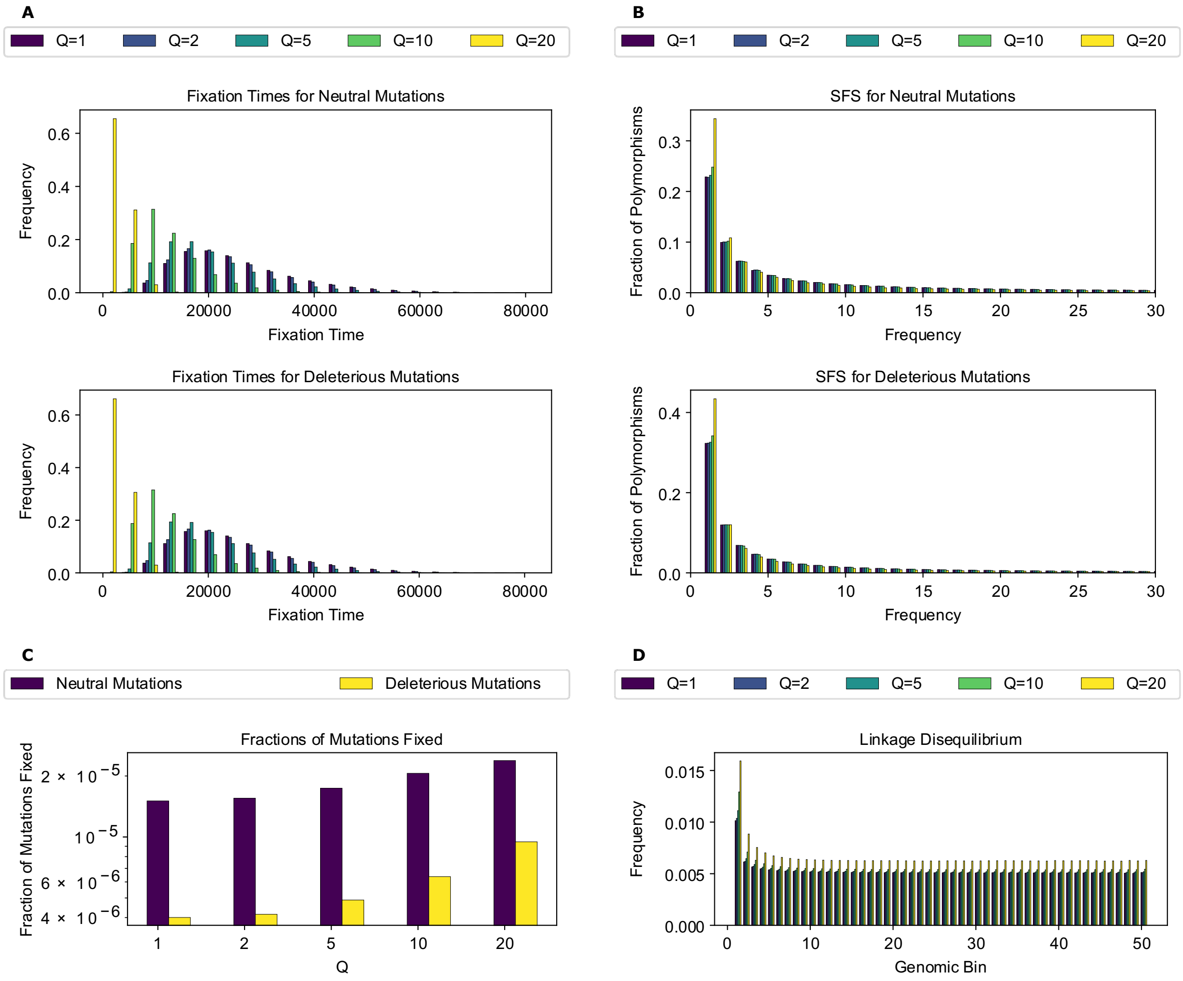

Figure S5. Simulation outcomes for the no-beneficials larger population model.
(A) Fixation time distributions for all mutation types. **(B)** Site frequency spectra for all mutation types. **(C)** Fractions of mutations fixed for all mutation types. **(D)** Linkage disequilibrium across the chromosome for 50 genomic bins, as measured by r^2.

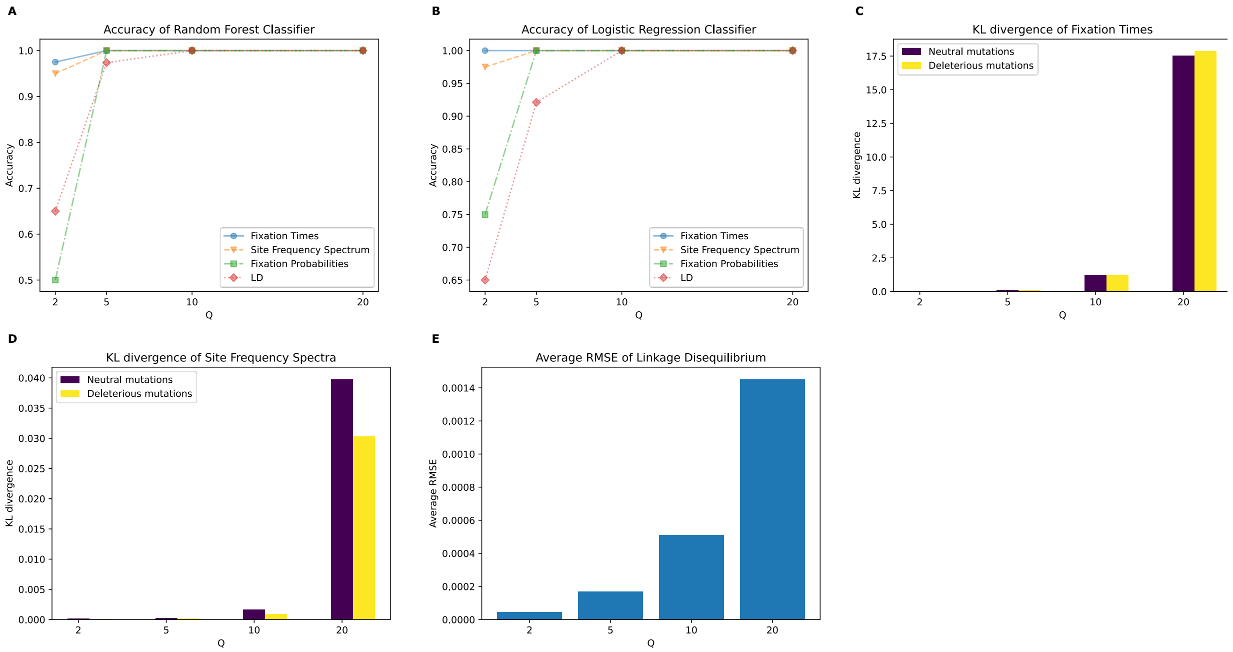

Figure S6. Metrics of the effect of scaling for the no-beneficials larger population model.
(A) Accuracy of the logistic regression classification using features subsets for each scaling factor. **(B)** Accuracy of the random forest classification using feature subsets for each scaling factor. **(C)** KL divergence of fixation time distributions for all mutation types. **(D)** KL divergence for the SFS for all mutation types. **(E)** Average RMSE of LD. Values and errors bars for panels **(C)**, **(D)**, and **(E)**, represent mean and standard deviation of 1000 bootstrapped samples.

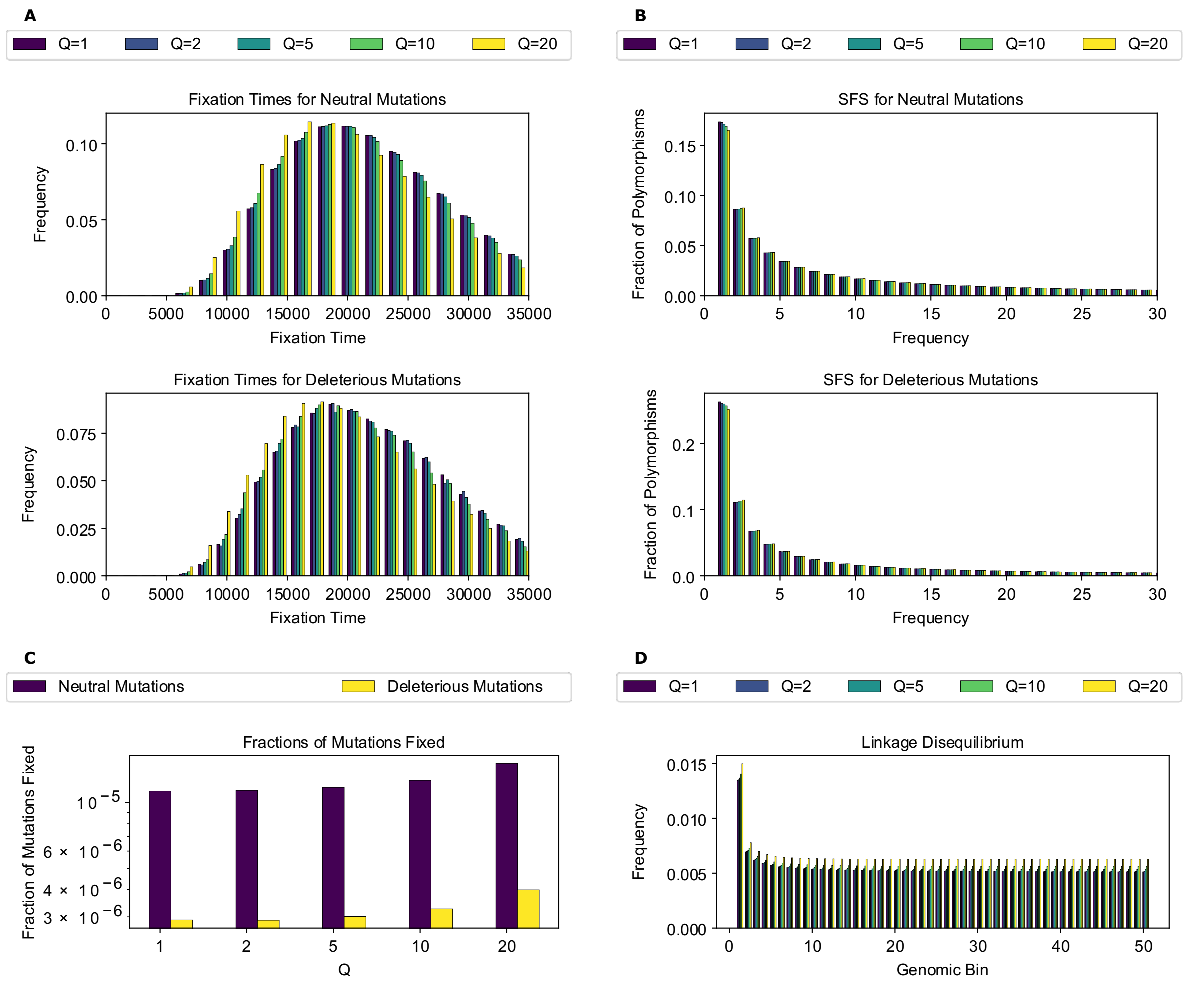

Figure S7. Simulation outcomes for the no-beneficials sparse model.
(A) Fixation time distributions for all mutation types. **(B)** Site frequency spectra for all mutation types. **(C)** Fractinos of mutations fixed for all mutation types. **(D)** Linkage disequilibrium across the chromosome for 50 genomic bins, as measured by r^2.

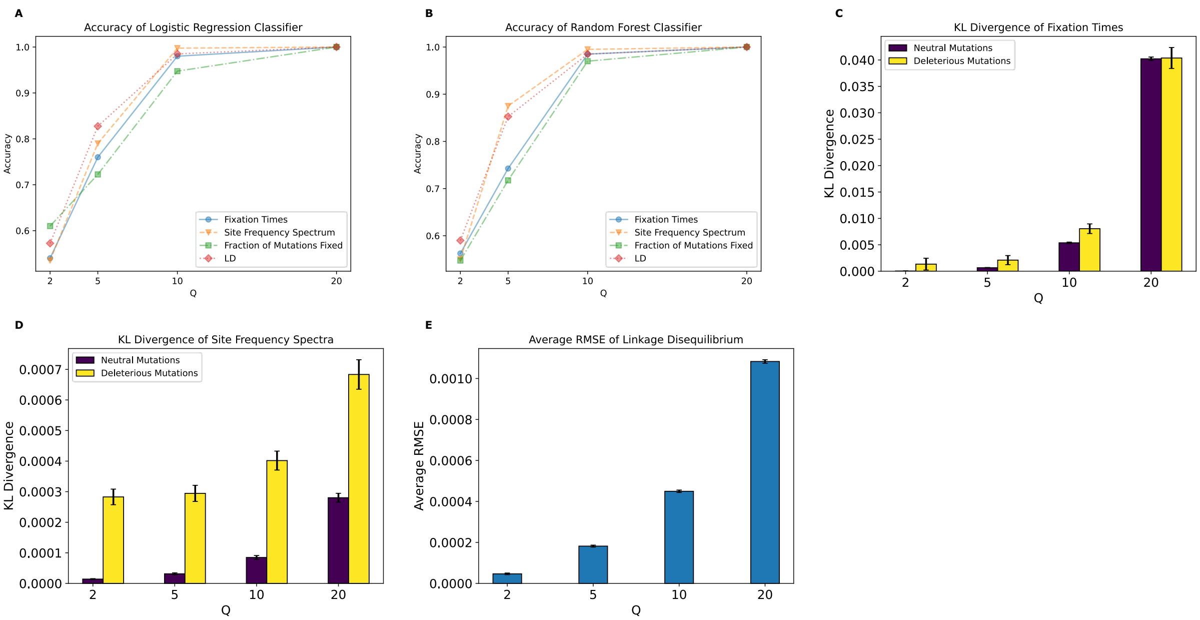

Figure S8. Metrics of the effect of scaling for the no-beneficials sparse model.
(A) Accuracy of the logistic regression classification using features subsets for each scaling factor. **(B)** Accuracy of the random forest classification using feature subsets for each scaling factor. **(C)** KL divergence of fixation time distributions for all mutation types. **(D)** KL divergence for the SFS for all mutation types. **(E)** Average RMSE of LD. Values and errors bars for panels **(C)**, **(D)**, and **(E)**, represent mean and standard deviation of 1000 bootstrapped samples.

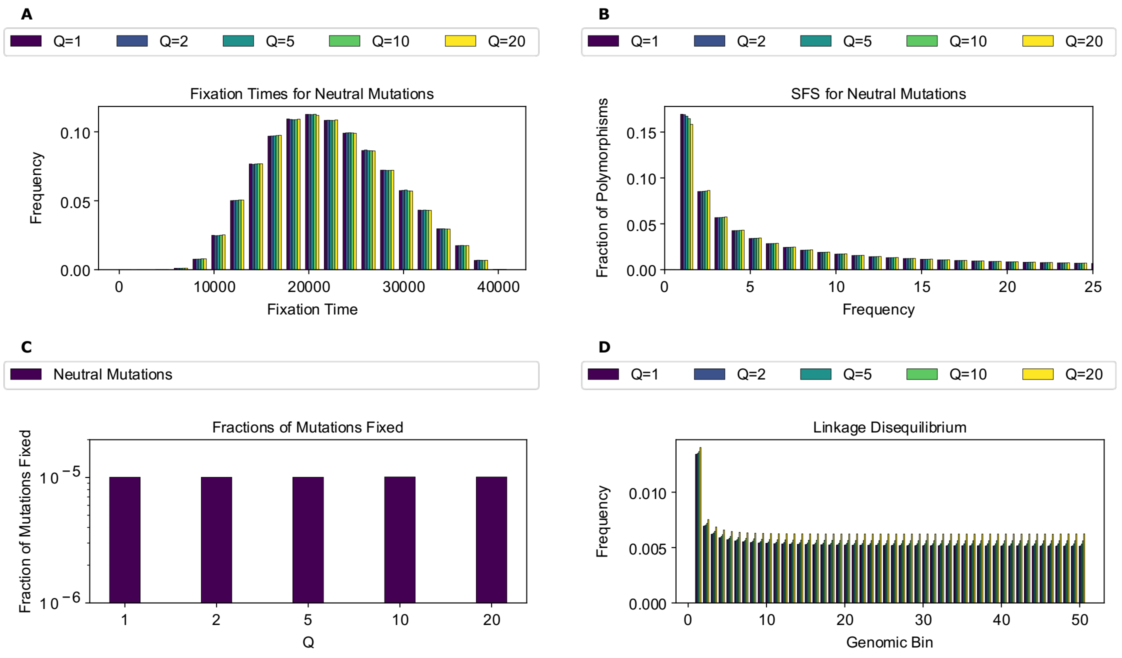

Figure S9. Simulation outcomes for the strictly neutral model.
(A) Fixation time distributions for neutral mutations. **(B)** Site frequency spectra for neutral mutations. **(C)** Fraction of neutral mutations fixed. **(D)** Linkage disequilibrium across the chromosome for 50 genomic bins, as measured by r^2.

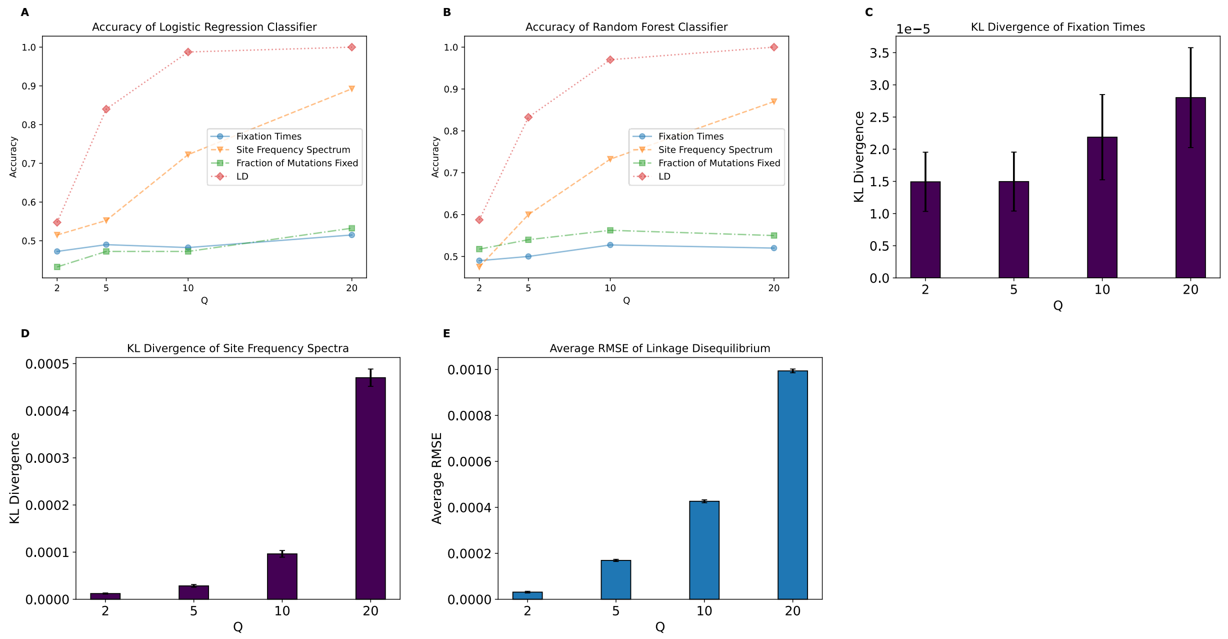

Figure S10. Metrics of the effect of scaling for the strictly neutral model.
(A) Accuracy of the logistic regression classification using features subsets for each scaling factor. **(B)** Accuracy of the random forest classification using feature subsets for each scaling factor. **(C)** KL divergence of fixation time distributions for neutral mutations. **(D)** KL divergence for the SFS for neutral mutations. **(E)** Average RMSE of LD for neutral mutations. Values and errors bars for panels **(C)**, **(D)**, and **(E)**, represent mean and standard deviation of 1000 bootstrapped samples.

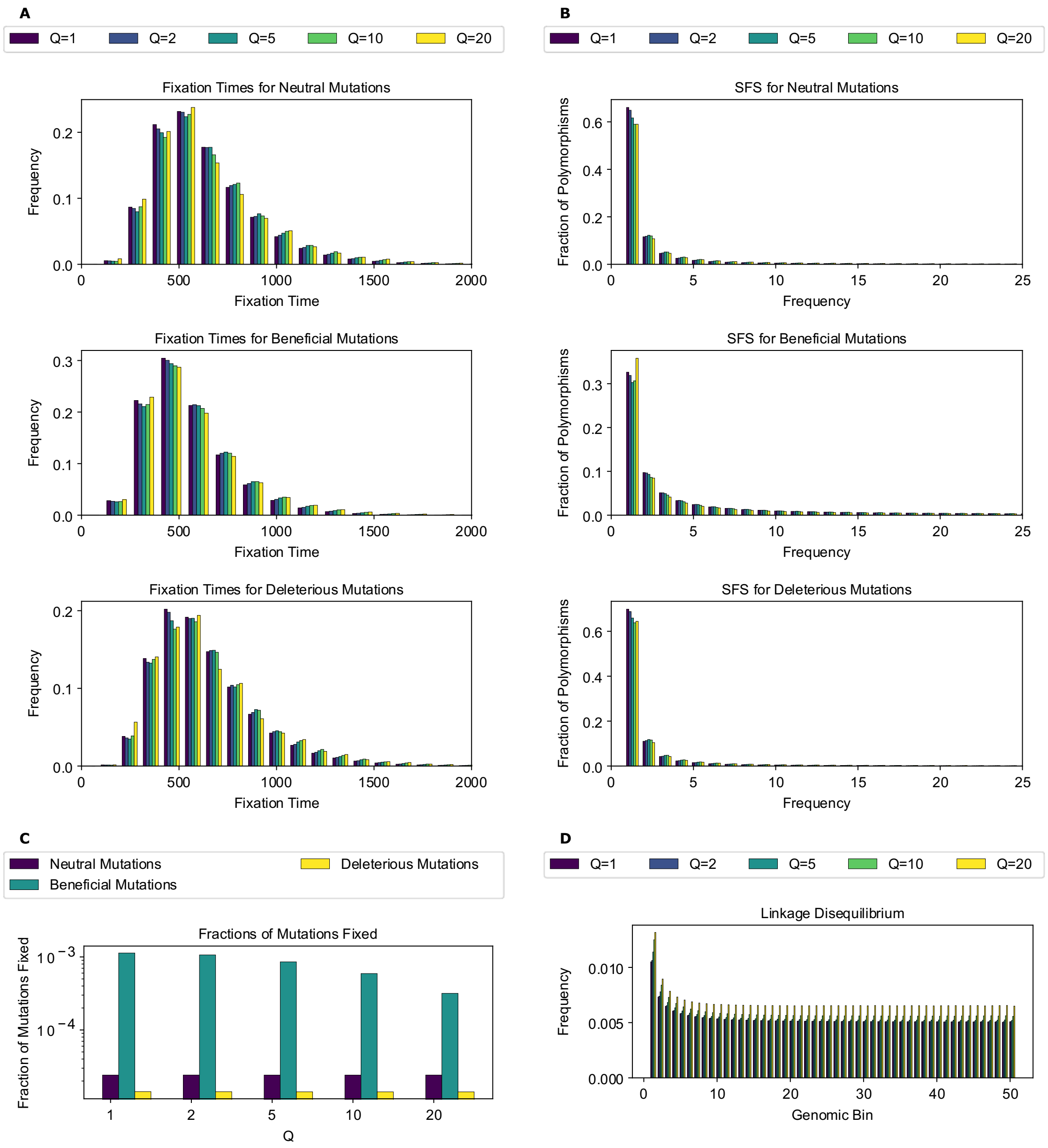

Figure S11. Simulation outcomes for the population expansion model.
(A) Fixation time distributions for all mutation types. **(B)** Site frequency spectra for all mutation types. **(C)** Fractions of mutations fixed for all mutation types. **(D)** Linkage disequilibrium across the chromosome for 50 genomic bins, as measured by r^2.

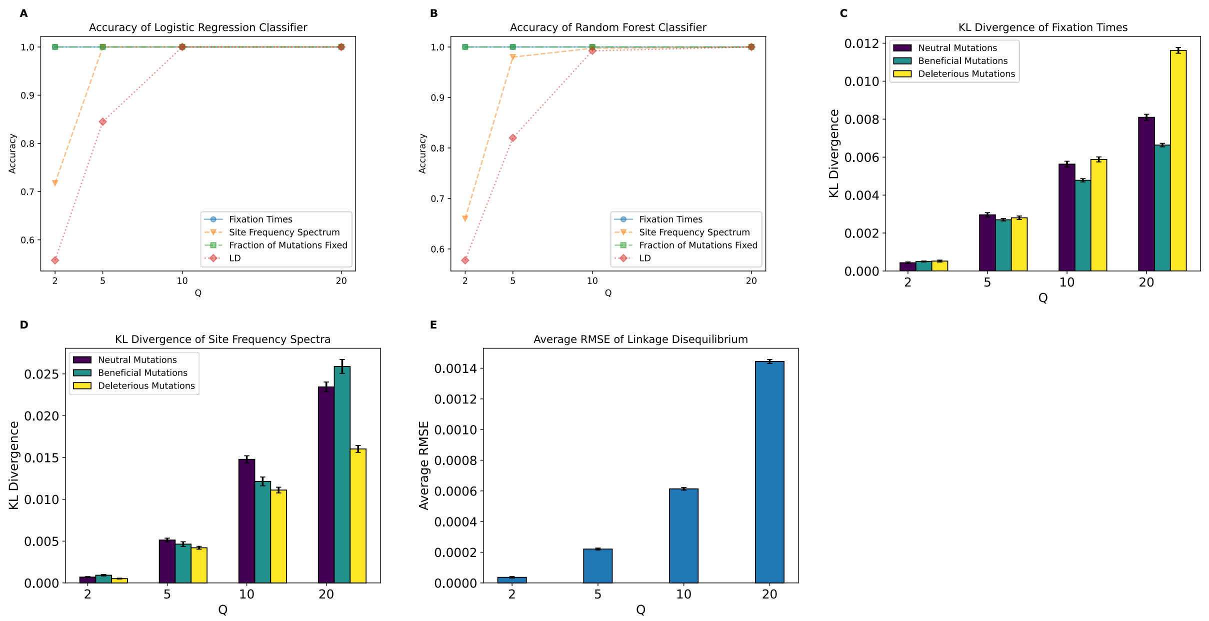

Figure S12. Metrics of the effect of scaling for the population expansion model.
(A) Accuracy of the logistic regression classification using features subsets for each scaling factor. **(B)** Accuracy of the random forest classification using feature subsets for each scaling factor. **(C)** KL divergence of fixation time distributions for all mutation types. **(D)** KL divergence for the SFS for all mutation types. **(E)** Average RMSE of LD. Values and errors bars for panels **(C)**, **(D)**, and **(E)**, represent mean and standard deviation of 1000 bootstrapped samples.

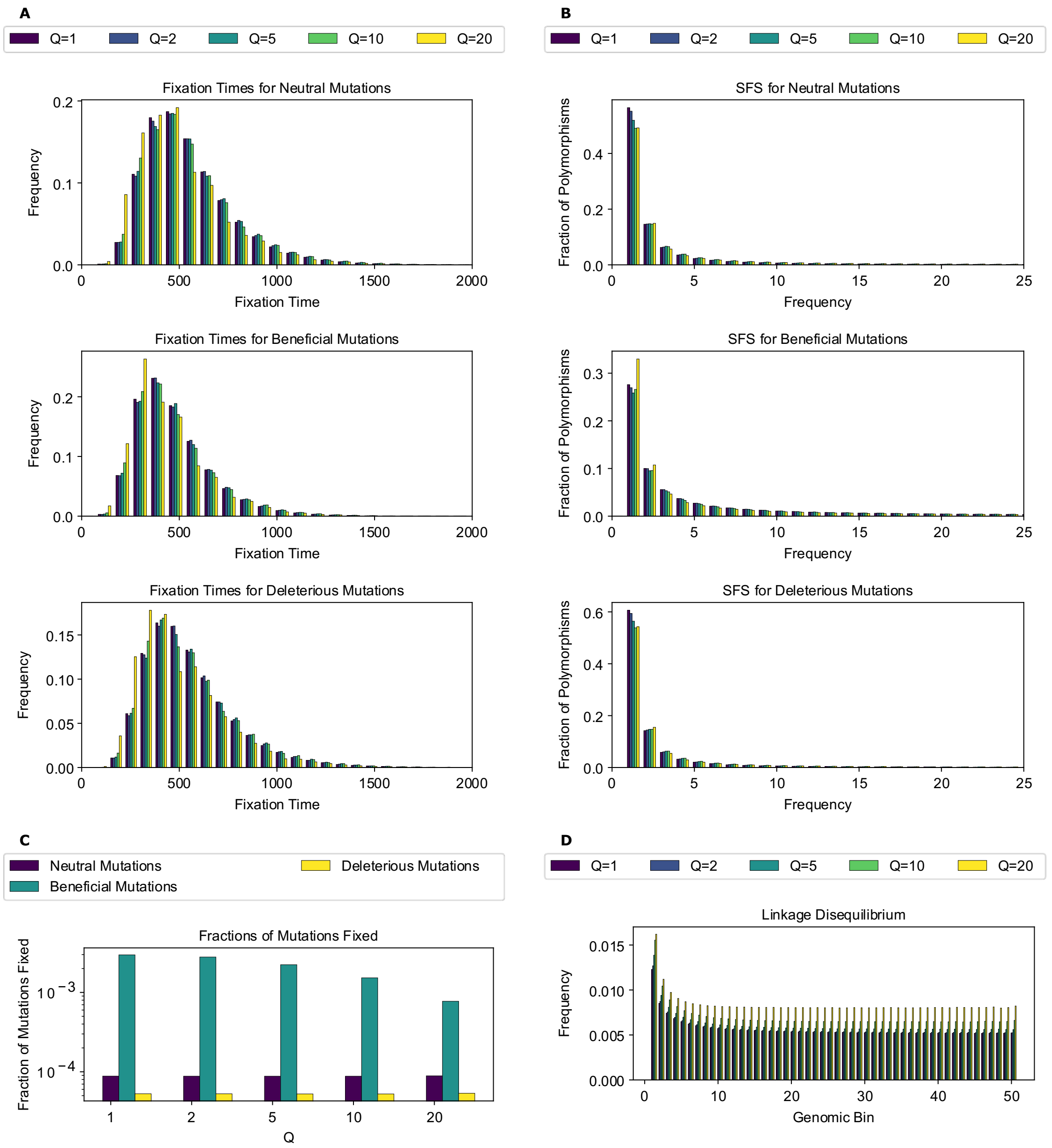

Figure S13. Simulation outcomes for the population contraction model.
(A) Fixation time distributions for all mutation types. **(B)** Site frequency spectra for all mutation types. **(C)** Fractions of mutations fixed for all mutation types. **(D)** Linkage disequilibrium across the chromosome for 50 genomic bins, as measured by r^2.

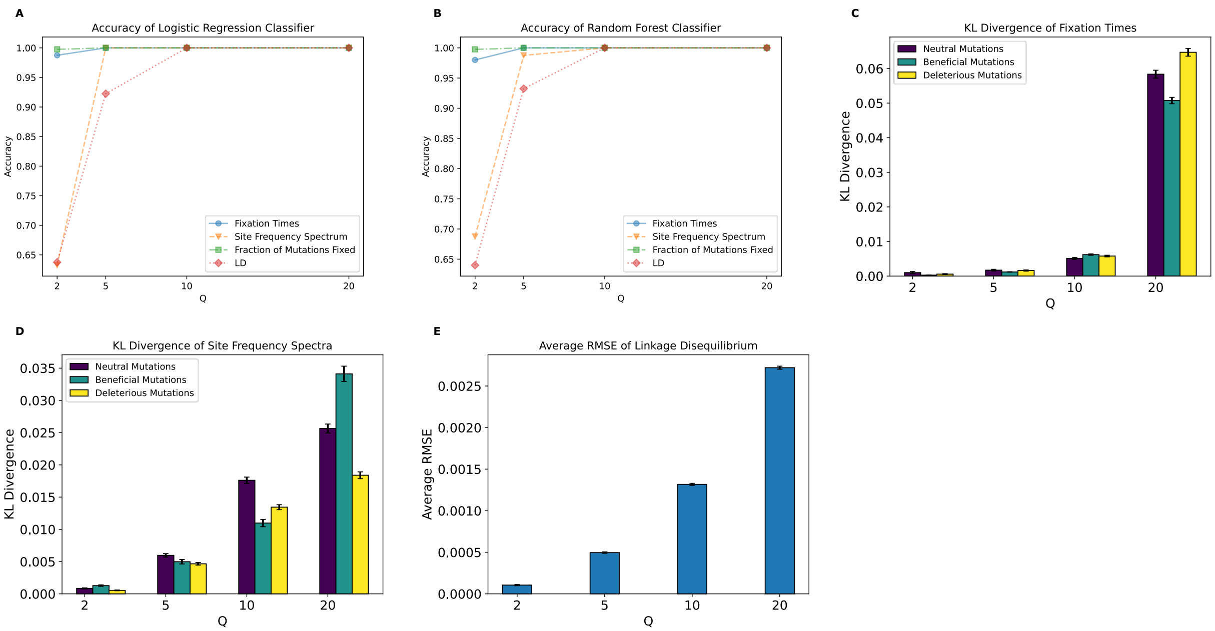

Figure S14. Metrics of the effect of scaling for the population contraction model.
(A) Accuracy of the logistic regression classification using features subsets for each scaling factor. **(B)** Accuracy of the random forest classification using feature subsets for each scaling factor. **(C)** KL divergence of fixation time distributions for all mutation types. **(D)** KL divergence for the SFS for all mutation types. **(E)** Average RMSE of LD. Values and errors bars for panels **(C)**, **(D)**, and **(E)**, represent mean and standard deviation of 1000 bootstrapped samples.

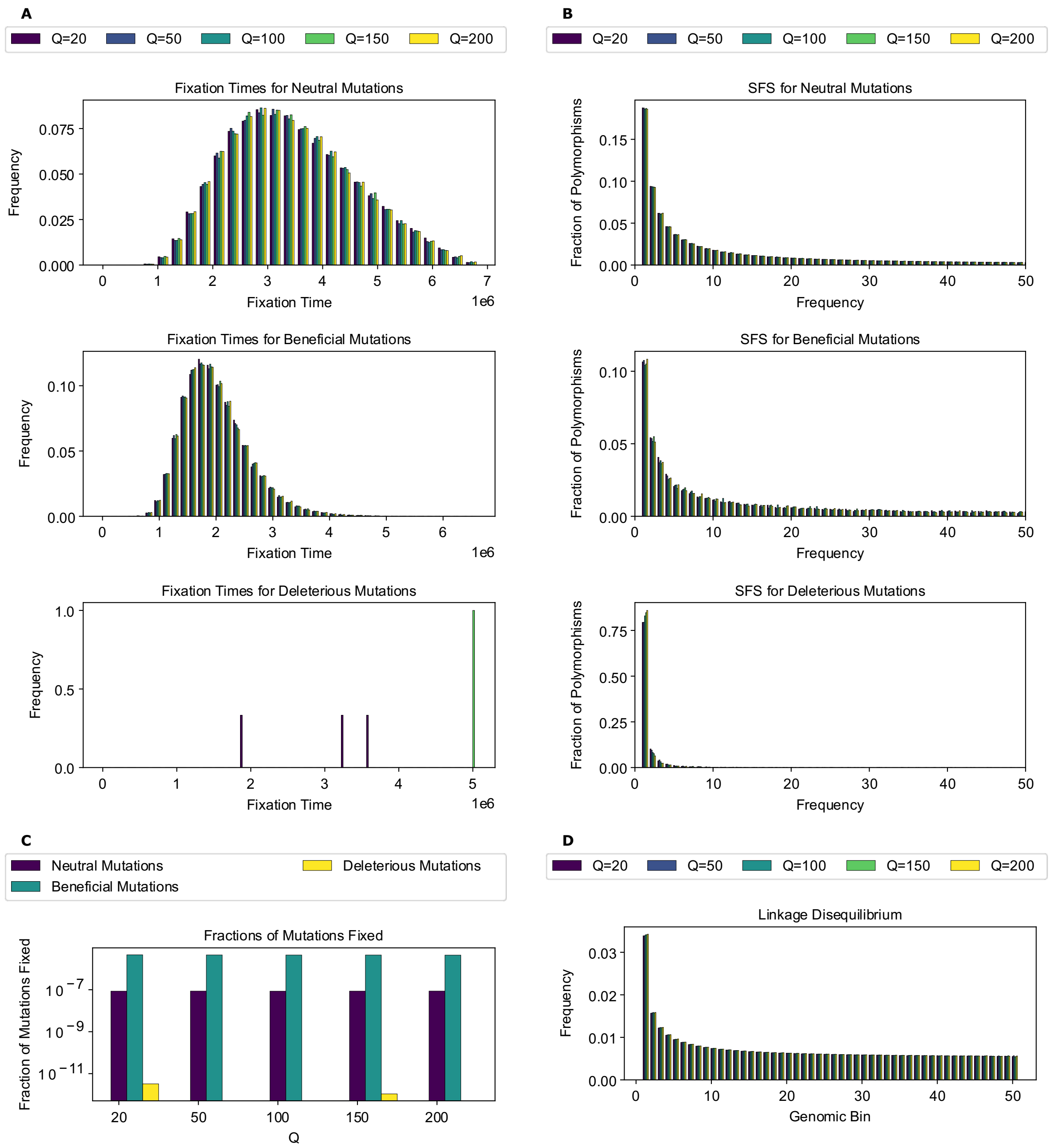

Figure S15. Simulation outcomes for the *Drosophila* model.
(A) Fixation time distributions for all mutation types. **(B)** Site frequency spectra for all mutation types. **(C)** Fractions of mutations fixed. for all mutation types. **(D)** Linkage disequilibrium across the chromosome for 50 genomic bins, as measured by r^2.

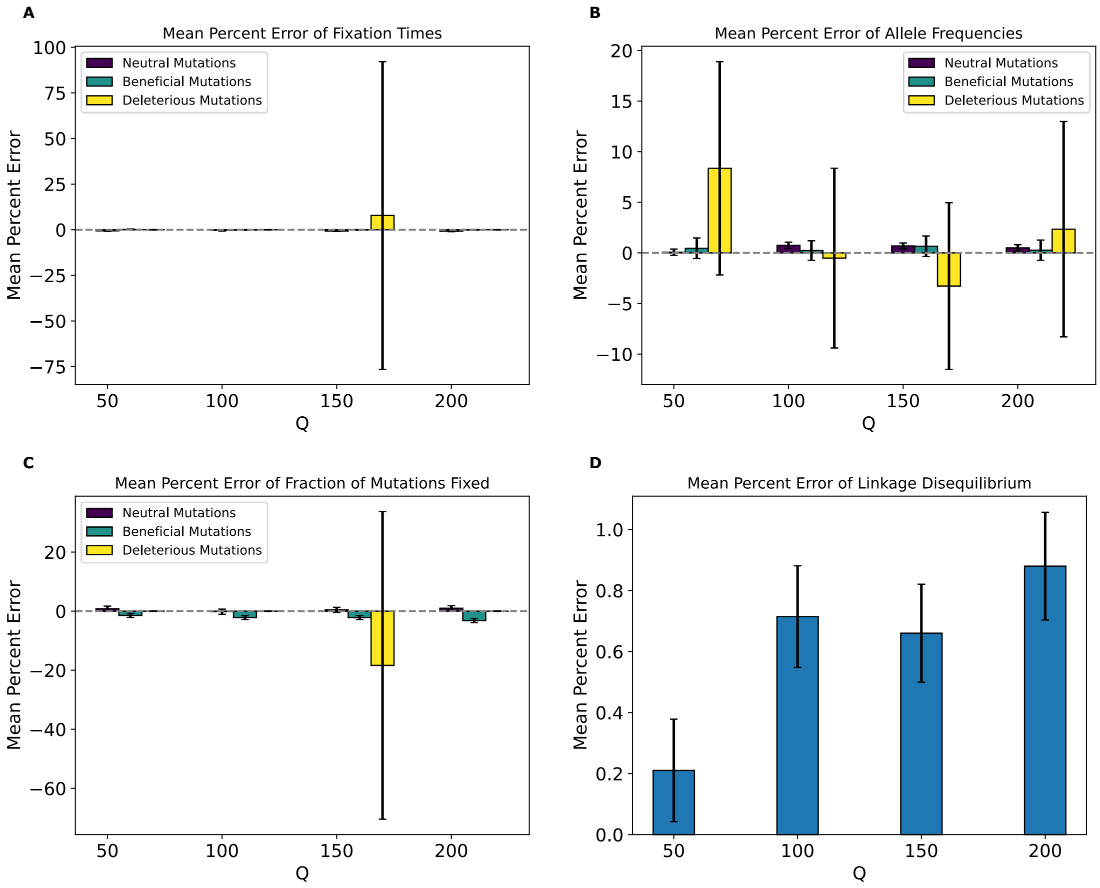

Figure S16. Mean percent error of various statistics for the *Drosophila* model by values of *Q*.
(A) Mean percent error for average mutation fixation times. **(B)** Mean percent error for average allele frequencies. **(C)** Mean percent error for average fractions of fixed mutations. **(D)** Mean percent error for average linkage disequilibrium as measured by the values of r^2. Values and errors bars represent mean and standard deviation of 1000 bootstrapped samples.

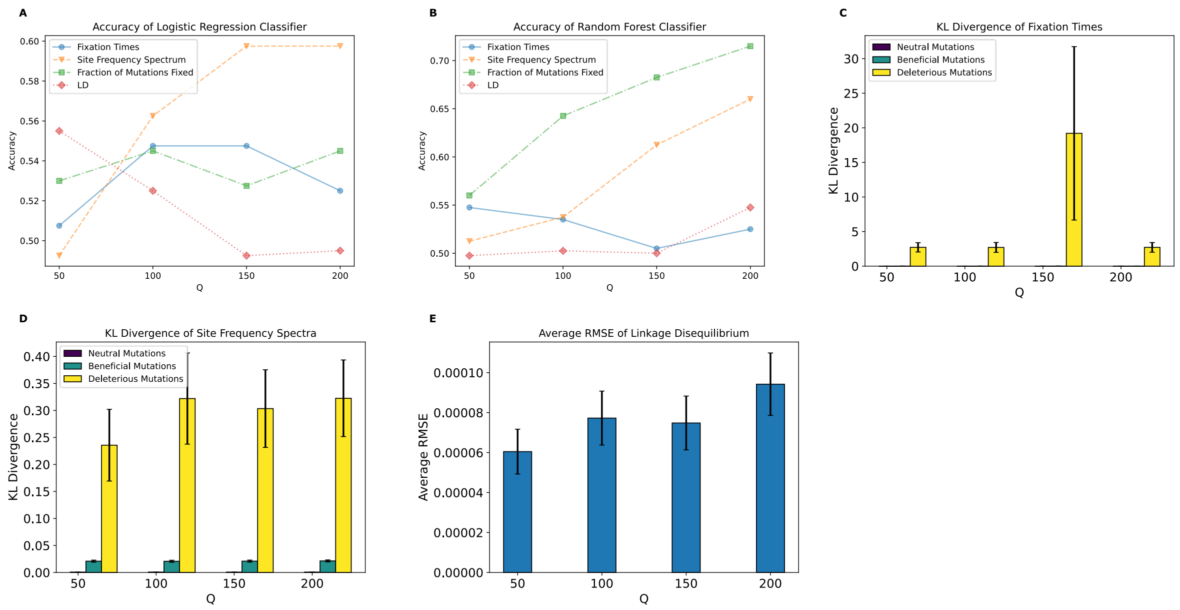

Figure S17. Metrics of the effect of scaling for the *Drosophila* model.
(A) Accuracy of the logistic regression classification using features subsets for each scaling factor. **(B)** Accuracy of the random forest classification using feature subsets for each scaling factor. **(C)** KL divergence of fixation time distributions for all mutation types. **(D)** KL divergence for the SFS for all mutation types. **(E)** Average RMSE of LD. Values and errors bars for panels **(C)**, **(D)**, and **(E)**, represent mean and standard deviation of 1000 bootstrapped samples.

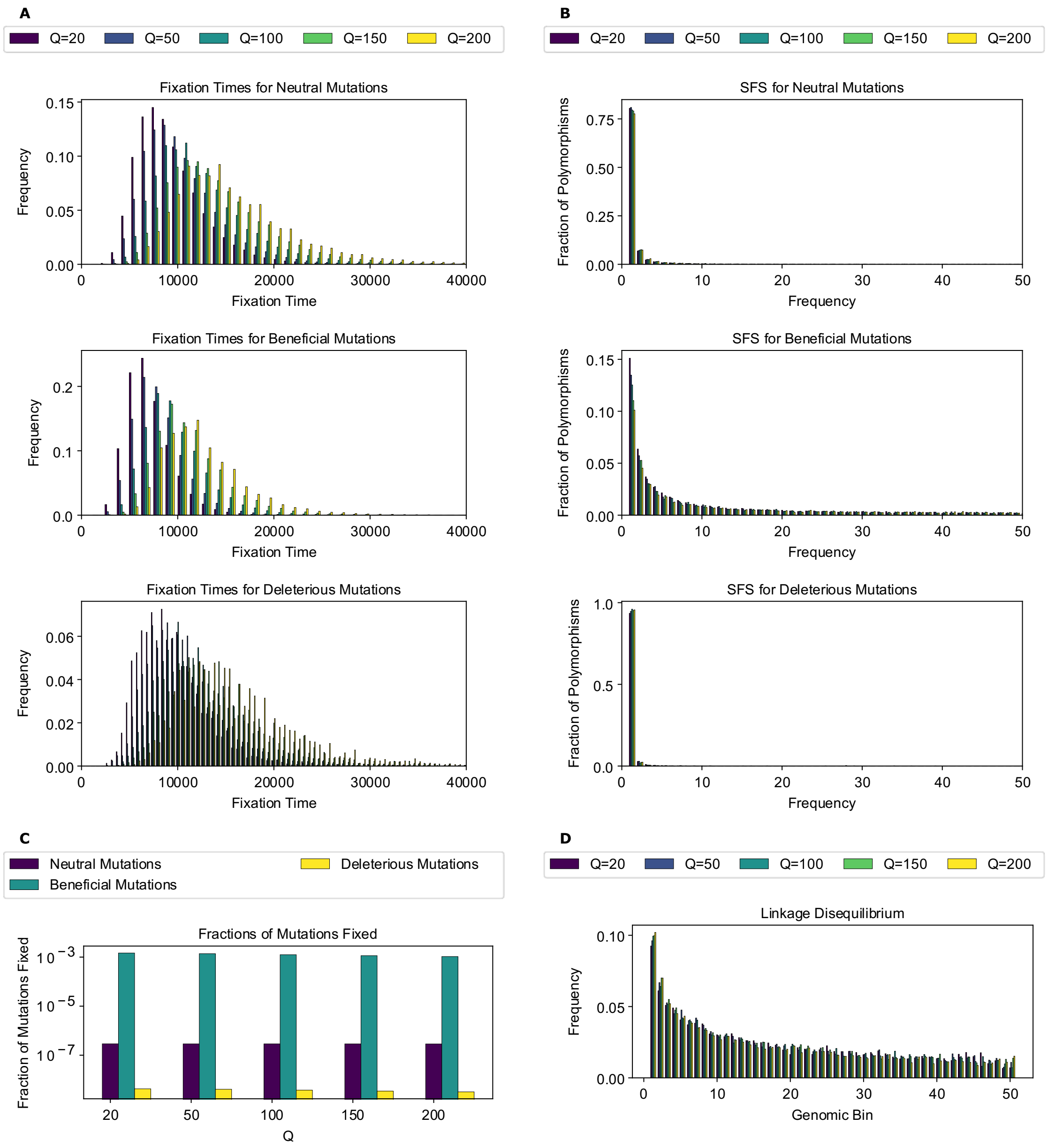

Figure S18. Simulation outcomes for the *Drosophila* stronger selection model.
(A) Fixation time distributions for all mutation types. **(B)** Site frequency spectra for all mutation types. **(C)** Fractions of mutations fixed for all mutation types. **(D)** Linkage disequilibrium across the chromosome for 50 genomic bins, as measured by r^2.

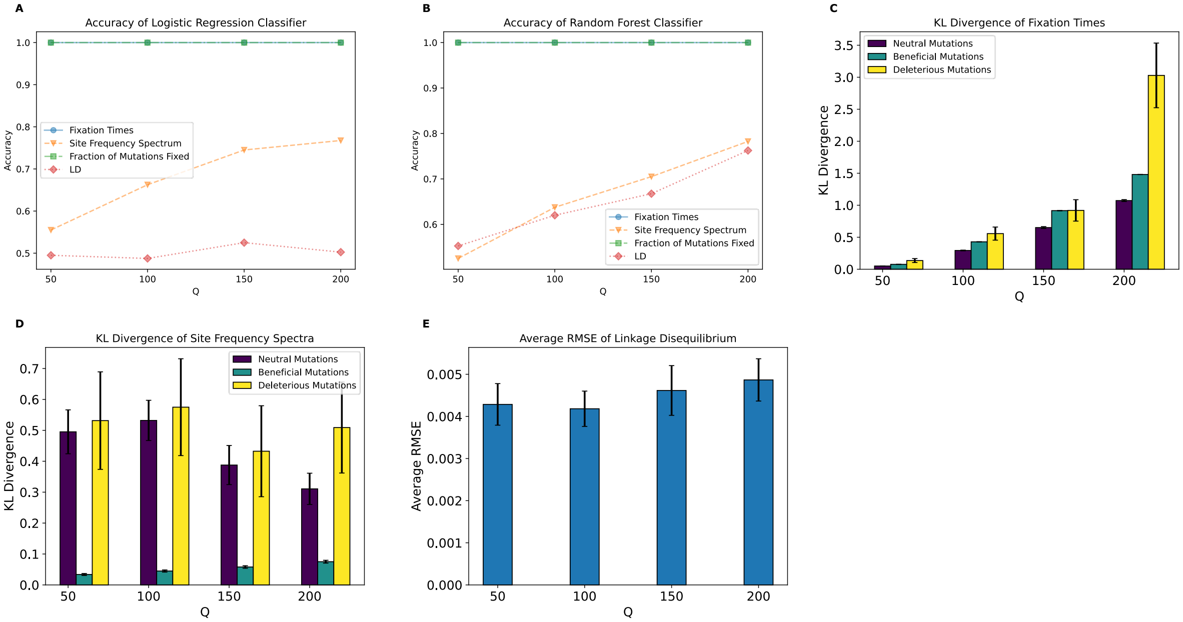

Figure S19. Metrics of the effect of scaling for the *Drosophila* stronger selection model.
(A) Accuracy of the logistic regression classification using features subsets for each scaling factor. **(B)** Accuracy of the random forest classification using feature subsets for each scaling factor. **(C)** KL divergence of fixation time distributions for all mutation types. **(D)** KL divergence for the SFS for all mutation types. **(E)** Average RMSE of LD. Values and errors bars for panels **(C)**, **(D)**, and **(E)**, represent mean and standard deviation of 1000 bootstrapped samples.

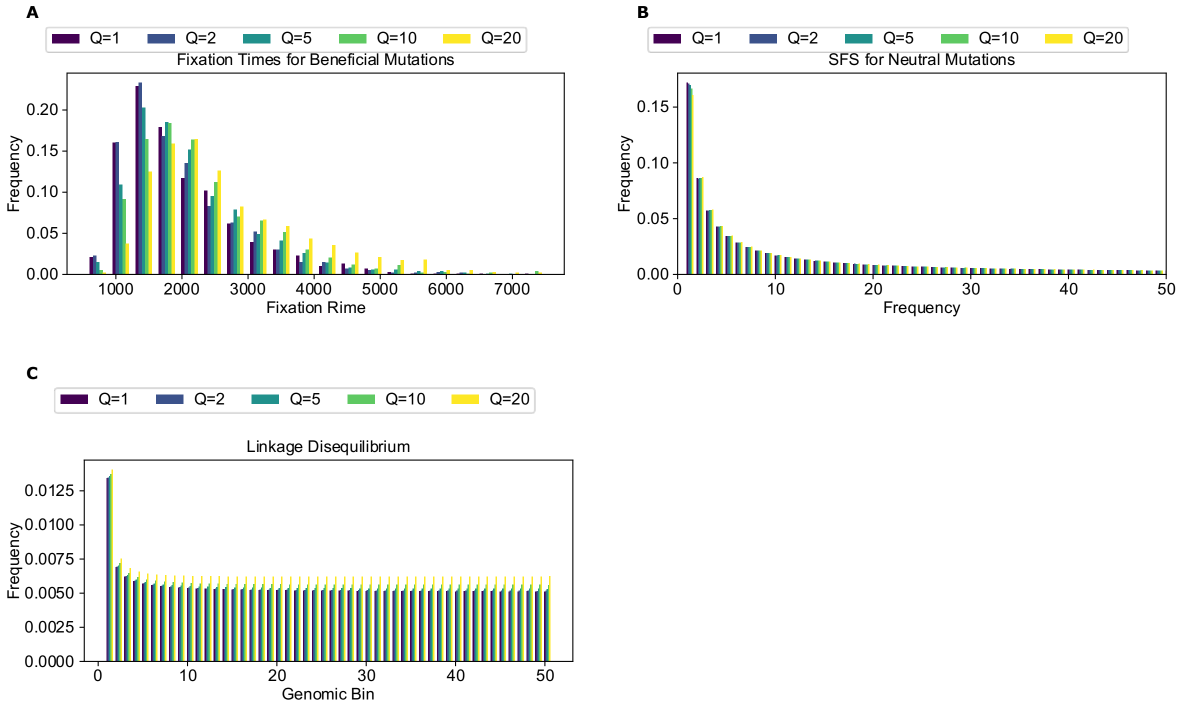

Figure S20. Simulation outcomes for the sweep model.
(A) Fixation time distributions for beneficial mutations. **(B)** Site frequency spectrum for neutral mutations. **(C)** Linkage disequilibrium for neutral mutations across the chromosome for 50 genomic bins, as measured by r^2.

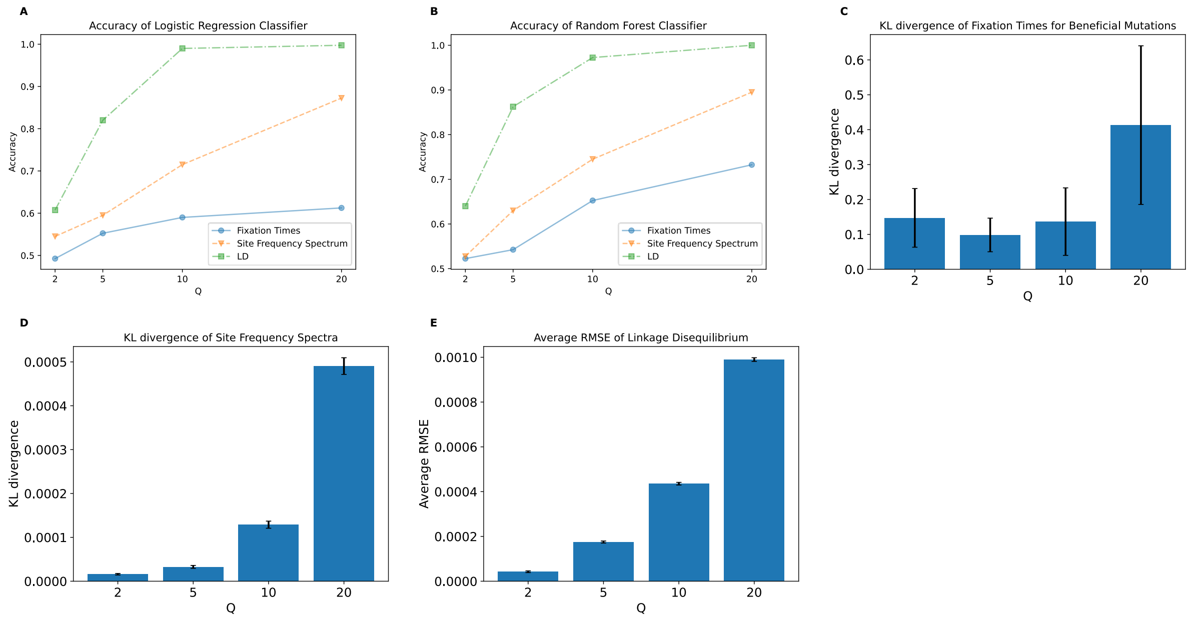

Figure S21. Metrics of the effect of scaling for the sweep model.
(A) Accuracy of the logistic regression classification using features subsets for each scaling factor. **(B)** Accuracy of the random forest classification using feature subsets for each scaling factor. **(C)** KL divergence of fixation time distributions for beneficial mutations. **(D)** KL divergence for the SFS for neutral mutations. **(E)** Average RMSE of LD for neutral mutations. Values and errors bars for panels **(C)**, **(D)**, and **(E)**, represent mean and standard deviation of 1000 bootstrapped samples.

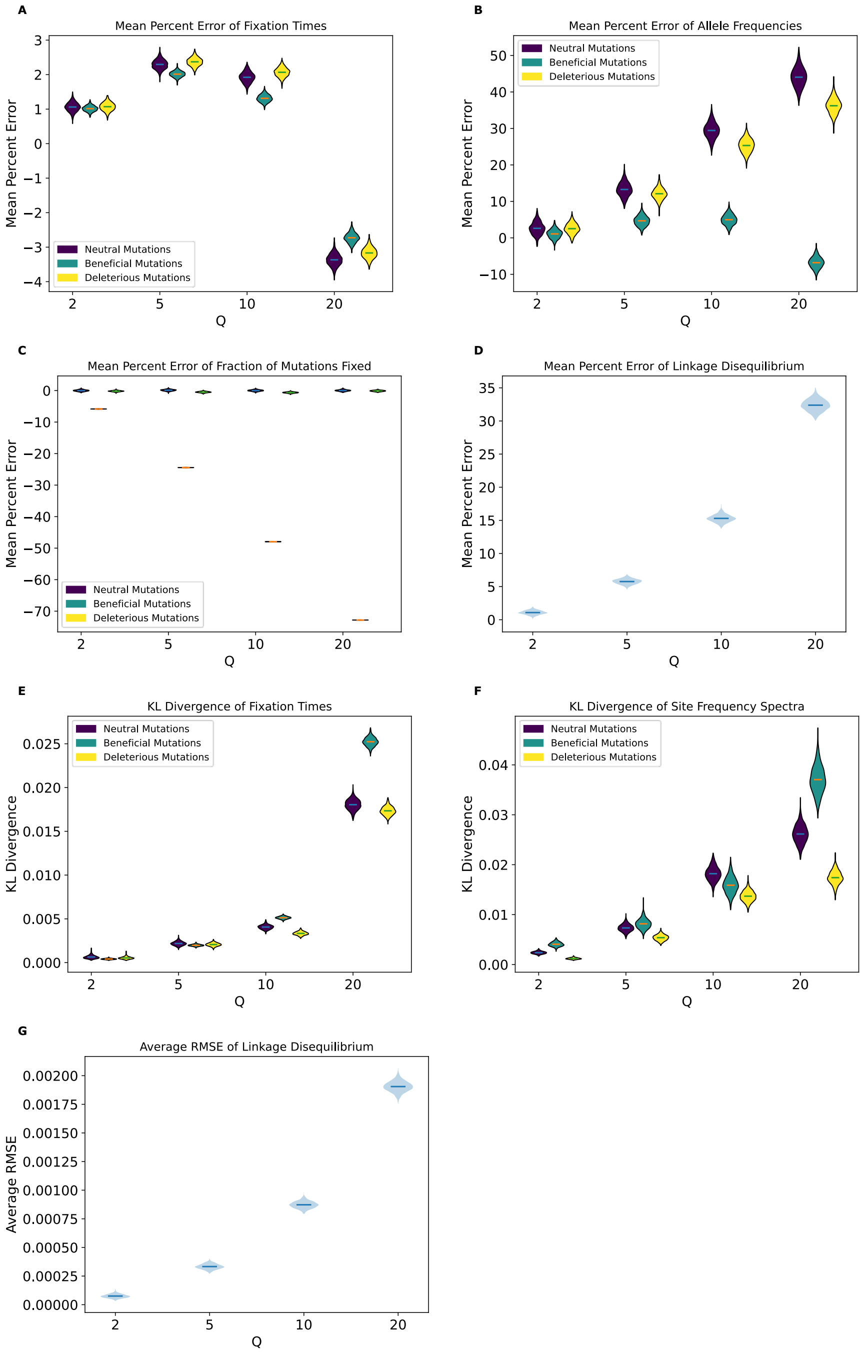

Figure S22. Measures of deviation for the full model using only 100 randomly selected replicates. **(A)** Mean percent error of average fixation times for all mutation types. **(B)** Mean percent error of average allele frequencies for all mutation types. **(C)** Mean percent error of average fractions of fixed mutations for all mutation types. **(D)** Mean percent error of average LD as measured by r^2. **(E)** KL Divergence of fixation times for all mutation types. **(F)** KL divergence of the SFS for all mutation types. **(G)** Average RMSE of LD as measured by r^2 across 50 genomic bins. Distributions are the result of repeating the sampling process 1000 times, with horizontal lines indicating the means of these distributions.

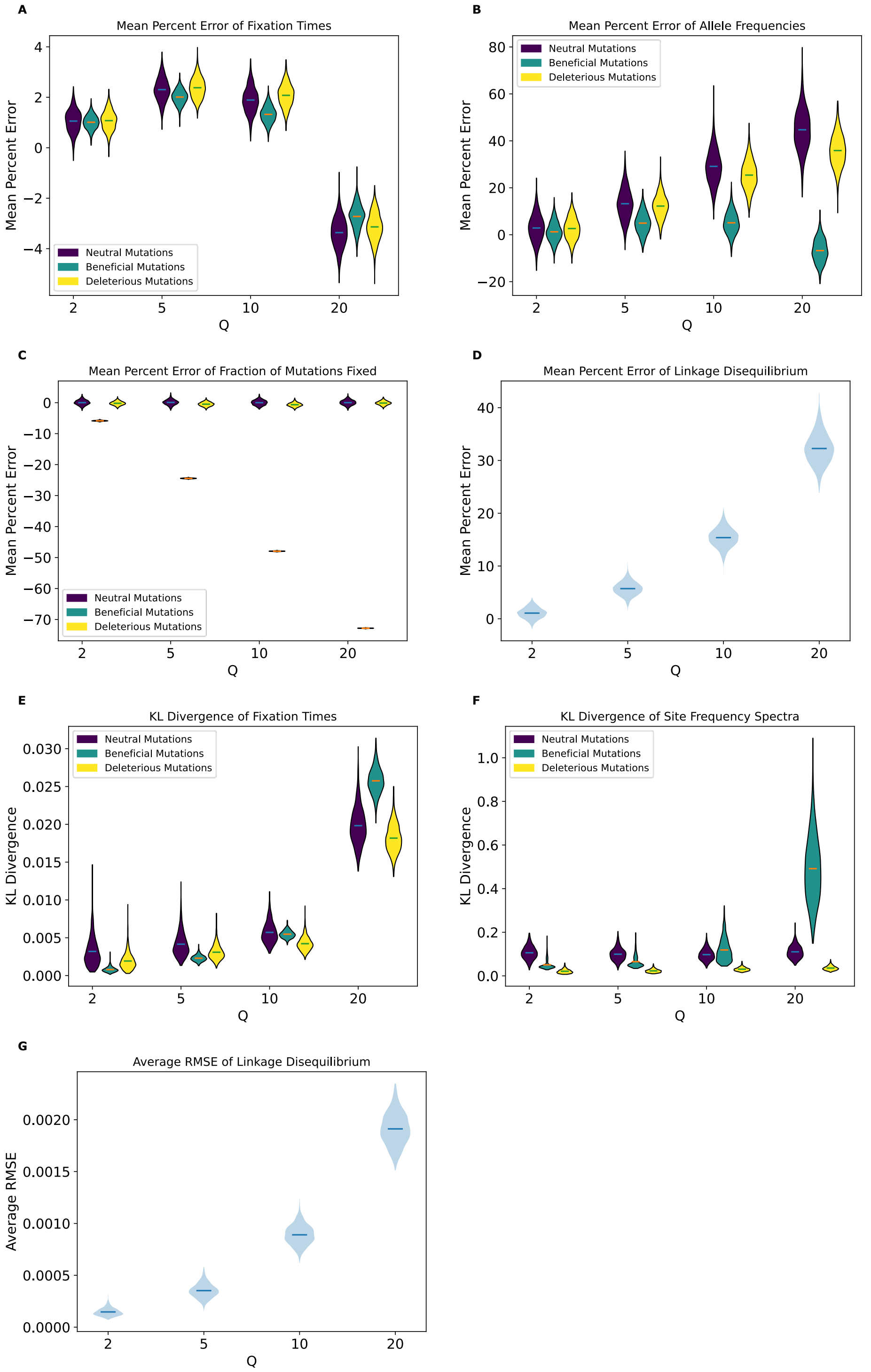

Figure S23. Measures of deviation for the full model using only 10 randomly selected replicates. **(A)** Mean percent error of average fixation times for all mutation types. **(B)** Mean percent error of average allele frequencies for all mutation types. **(C)** Mean percent error of average fractions of fixed mutations for all mutation types. **(D)** Mean percent error of average LD as measured by r^2. **(E)** KL Divergence of fixation times for all mutation types. **(F)** KL divergence of the SFS for all mutation types. **(G)** Average RMSE of LD as measured by r^2 across 50 genomic bins. Distributions are the result of repeating the sampling process 1000 times, with horizontal lines indicating the means of these distributions.

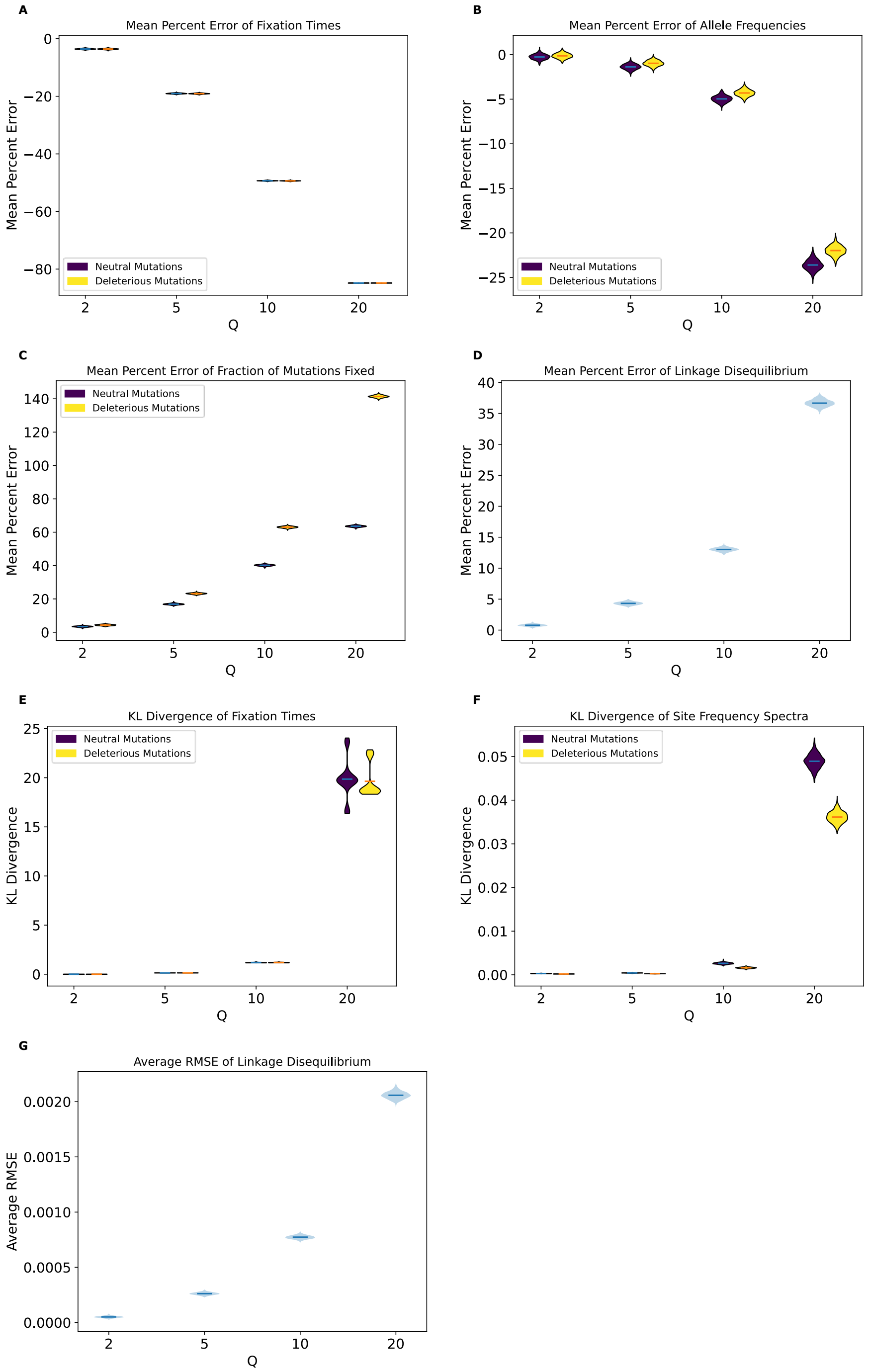

Figure S24. Measures of deviation for the no-beneficials model using only 100 randomly selected replicates. **(A)** Mean percent error of average fixation times for all mutation types. **(B)** Mean percent error of average allele frequencies for all mutation types. **(C)** Mean percent error of average fractions of fixed mutations for all mutation types. **(D)** Mean percent error of average LD as measured by r^2. **(E)** KL Divergence of fixation times for all mutation types. **(F)** KL divergence of the SFS for all mutation types. **(G)** Average RMSE of LD as measured by r^2 across 50 genomic bins. Distributions are the result of repeating the sampling process 1000 times, with horizontal lines indicating the means of these distributions.

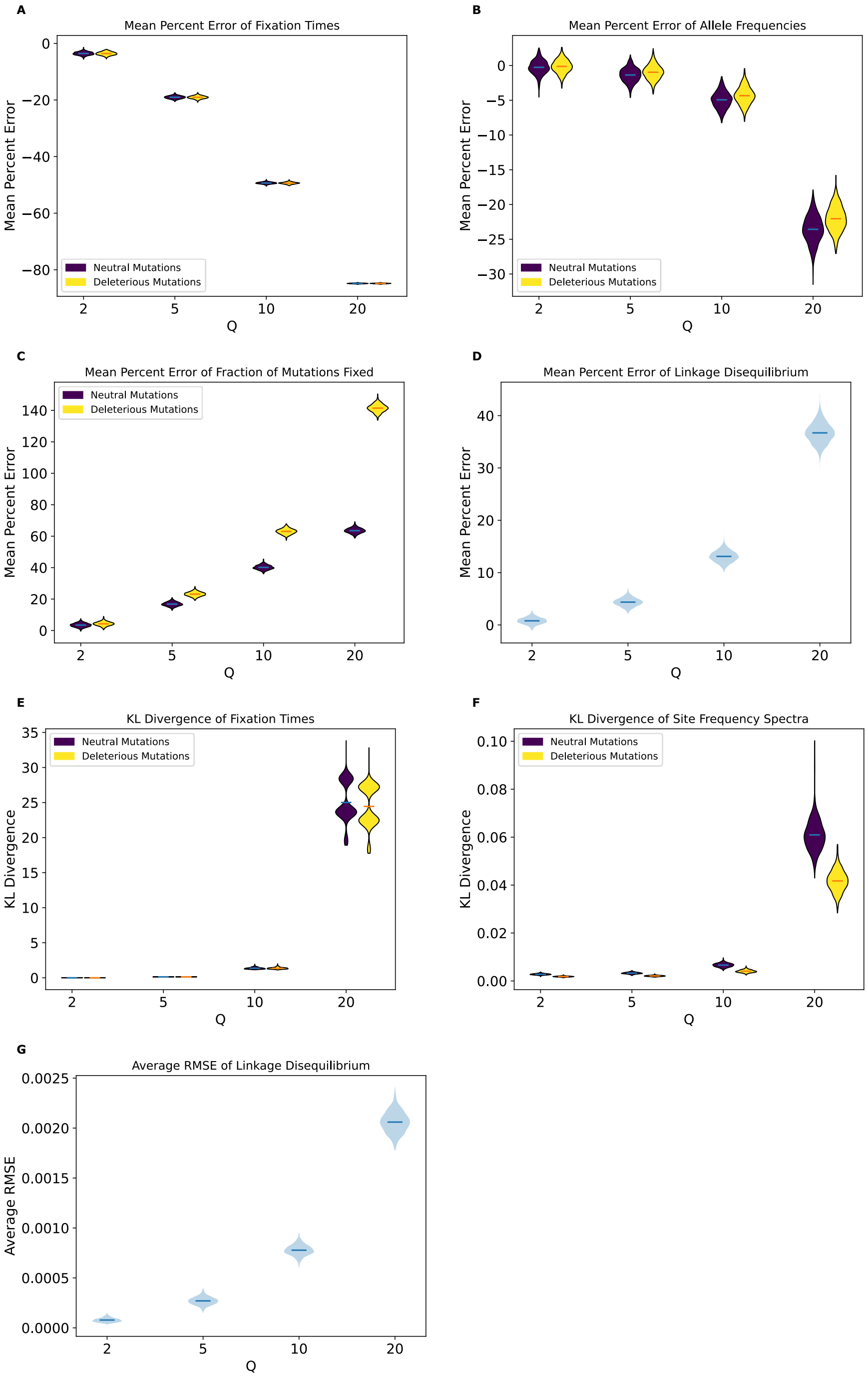

Figure S25. Measures of deviation for the no-beneficials model using only 10 randomly selected replicates. **(A)** Mean percent error of average fixation times for all mutation types. **(B)** Mean percent error of average allele frequencies for all mutation types. **(C)** Mean percent error of average fractions of fixed mutations for all mutation types. **(D)** Mean percent error of average LD as measured by r^2. **(E)** KL Divergence of fixation times for all mutation types. **(F)** KL divergence of the SFS for all mutation types. **(G)** Average RMSE of LD as measured by r^2 across 50 genomic bins. Distributions are the result of repeating the sampling process 1000 times, with horizontal lines indicating the means of these distributions.

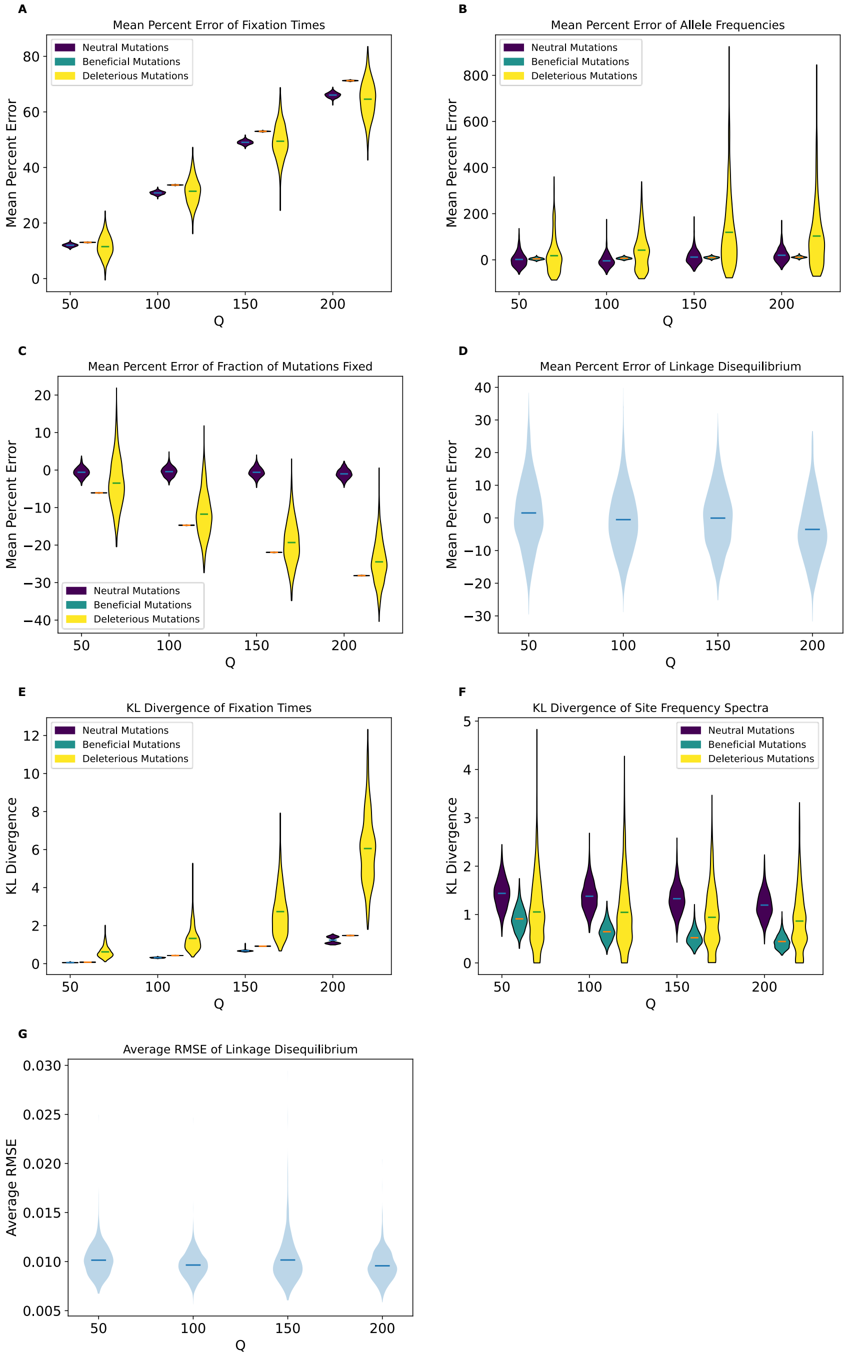

Figure S26. Measures of deviation for the *Drosophila* stronger selection model using only 100 randomly selected replicates.

(A) Mean percent error of average fixation times for all mutation types. **(B)** Mean percent error of average allele frequencies for all mutation types. **(C)** Mean percent error of average fractions of fixed mutations for all mutation types. **(D)** Mean percent error of average LD as measured by r^2. **(E)** KL Divergence of fixation times for all mutation types. **(F)** KL divergence of the SFS for all mutation types. **(G)** Average RMSE of LD as measured by r^2 across 50 genomic bins. Distributions are the result of repeating the sampling process 1000 times, with horizontal lines indicating the means of these distributions.

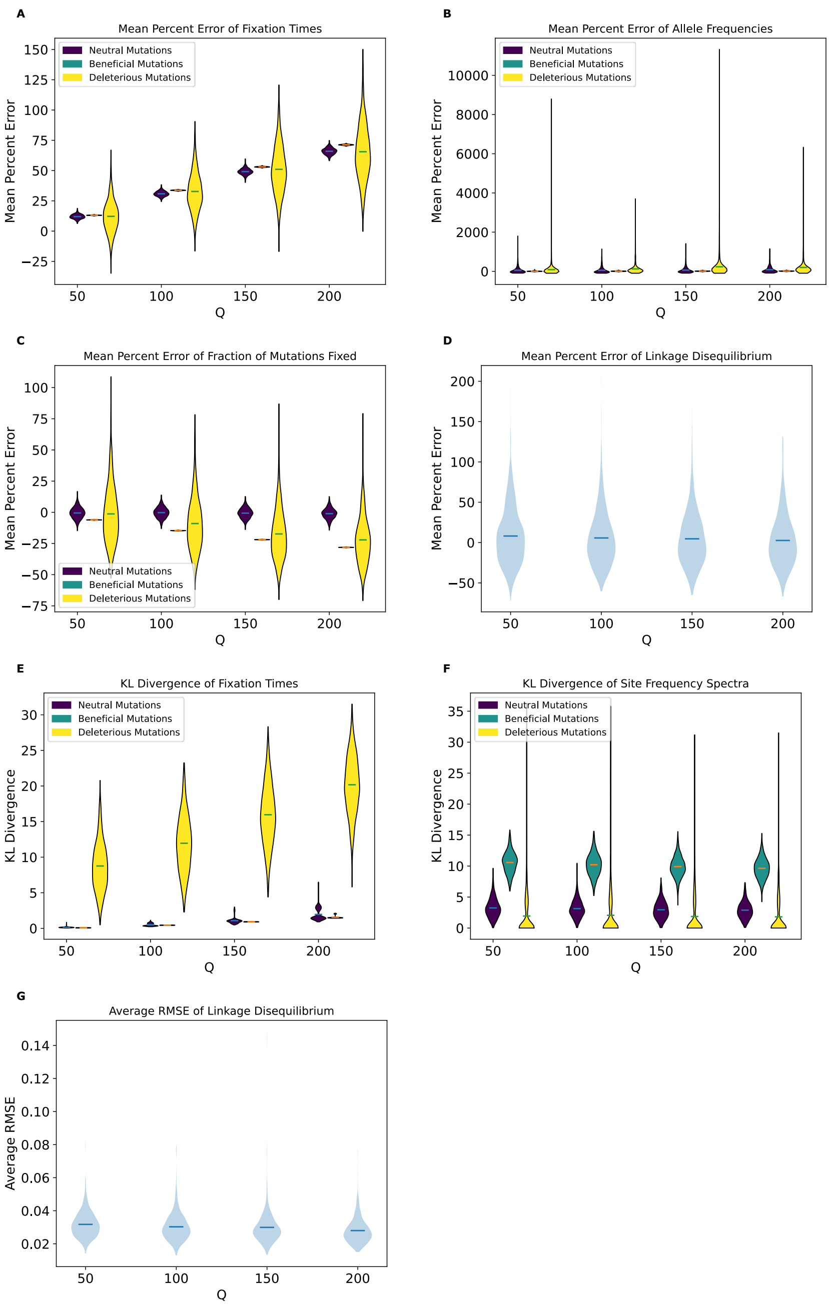

Figure S27. Measures of deviation for the *Drosophila* stronger selection model using only 10 randomly selected replicates.

(A) Mean percent error of average fixation times for all mutation types. **(B)** Mean percent error of average allele frequencies for all mutation types. **(C)** Mean percent error of average fractions of fixed mutations for all mutation types. **(D)** Mean percent error of average LD as measured by r^2. **(E)** KL Divergence of fixation times for all mutation types. **(F)** KL divergence of the SFS for all mutation types. **(G)** Average RMSE of LD as measured by r^2 across 50 genomic bins. Distributions are the result of repeating the sampling process 1000 times, with horizontal lines indicating the means of these distributions.

Table S1. Values of KL Divergence (for Fixation Times and SFS) and Average RMSE (for LD) for All Simulation Models.

Model	Mutation Type	Outcome	Q=2	Q=5	Q=10	Q=20
**Full Model**	Neutral	Fixation Times	0.00041	0.002	0.0039	0.018
		SFS	0.00074	0.0057	0.017	0.025
	Beneficial	Fixation Times	0.00037	0.0019	0.0051	0.025
		SFS	0.00099	0.0048	0.012	0.031
	Deleterious	Fixation Times	0.00039	0.002	0.0032	0.017
		SFS	0.00044	0.0047	0.013	0.017
	All	Linkage Disequilibrium	0.0000663	0.00033	0.00087	0.0019
**No-Beneficials**	Neutral	Fixation Times	0.0045	0.13	1.17	17.2
		SFS	0.0000636	0.00017	0.0023	0.048
	Deleterious	Fixation Times	0.0044	0.13	1.18	18.7
		SFS	0.0000388	0.000089	0.0014	0.036
	All	Linkage Disequilibrium	0.0000478	0.00026	0.00077	0.0021
**No-Beneficials Sparse**	Neutral	Fixation Times	0.0000418	0.00066	0.0054	0.04
		SFS	0.0000141	3.14E-05	8.45E-05	0.00028
	Deleterious	Fixation Times	0.0013	0.0021	0.008	0.04
		SFS	0.00028	0.00029	0.0004	0.00068
	All	Linkage Disequilibrium	0.0000465	0.00018	0.00045	0.0011
**No-Beneficials Larger Population**	Neutral	Fixation Times	0.0045	0.14	1.22	25.3
		SFS	0.00033	0.00037	0.0017	0.04
	Deleterious	Fixation Times	0.0045	0.14	1.23	22.5
		SFS	0.0002	0.00023	0.0012	0.03
	All	Linkage Disequilibrium	0.0000521	0.00019	0.00053	0.0015
**Strictly Neutral**	Neutral	Fixation Times	0.000015	1.49E-05	2.23E-05	2.77E-05
		SFS	0.000012	2.86E-05	9.62E-05	0.00047
	Neutral	Linkage Disequilibrium	0.0000314	0.00017	0.00043	0.00099
**Population Expansion**	Neutral	Fixation Times	0.00043	0.003	0.0056	0.0081
		SFS	0.0007	0.0051	0.015	0.023
	Beneficial	Fixation Times	0.0005	0.0027	0.0048	0.0066
		SFS	0.00092	0.0047	0.012	0.026
	Deleterious	Fixation Times	0.00052	0.0028	0.0059	0.012
		SFS	0.00051	0.0042	0.011	0.016
	All	Linkage Disequilibrium	0.0000364	0.00022	0.00061	0.0014
**Population Contraction**	Neutral	Fixation Times	0.00098	0.0017	0.0051	0.058
		SFS	0.00085	0.006	0.018	0.026
	Beneficial	Fixation Times	0.00029	0.0012	0.0062	0.051
		SFS	0.0013	0.005	0.011	0.034
	Deleterious	Fixation Times	0.00056	0.0016	0.0058	0.065
		SFS	0.00054	0.0047	0.013	0.018
	All	Linkage Disequilibrium	0.00011	0.0005	0.0013	0.0027
**Sweep**	Neutral	SFS	1.60E-05	3.26E-05	1.29E-04	4.92E-04

		Linkage Disequilibrium	4.23E-05	1.75E-04	4.36E-04	9.89E-04
	Beneficial	Fixation Times	0.14	0.096	0.14	0.41
			**Q=50**	**Q=100**	**Q=150**	**Q=200**
Drosophila	Neutral	Fixation Times	0.0025	0.0026	0.0044	0.0045
		SFS	0.00077	0.00081	0.00074	0.00076
	Beneficial	Fixation Times	0.0054	0.0052	0.0075	0.0036
		SFS	0.021	0.021	0.021	0.021
	Deleterious	Fixation Times	2.66	2.68	18.8	2.69
		SFS	0.24	0.33	0.3	0.32
	All	Linkage Disequilibrium	0.0000605	7.77E-05	7.49E-05	9.47E-05
***Drosophila* Stronger Selection**	Neutral	Fixation Times	0.051	0.29	0.65	1.07
		SFS	0.5	0.54	0.39	0.31
	Beneficial	Fixation Times	0.077	0.43	0.92	1.48
		SFS	0.034	0.045	0.058	0.076
	Deleterious	Fixation Times	0.14	0.57	0.92	3.02
		SFS	0.53	0.57	0.43	0.51
	All	Linkage Disequilibrium	0.0043	0.0042	0.0046	0.0049

Table S2. Classifier Accuracy for All Simulation Models.

Model	Outcome	Classification Method	Q=2	Q=5	Q=10	Q=20
**Full Model**	Fixation Times	Logistic Regression	100%	100%	100%	100%
		Random Forest	100%	100%	100%	100%
	SFS	Logistic Regression	72%	100%	100%	100%
		Random Forest	73%	97%	100%	100%
	Fraction of Mutations Fixed	Logistic Regression	100%	100%	100%	100%
		Random Forest	100%	100%	100%	100%
	Linkage Disequilibrium	Logistic Regression	60%	90%	100%	100%
		Random Forest	60%	91%	100%	100%
**No-beneficials**	Fixation Times	Logistic Regression	96%	100%	100%	100%
		Random Forest	97%	100%	100%	100%
	SFS	Logistic Regression	91%	100%	100%	100%
		Random Forest	91%	100%	100%	100%
	Fraction of Mutations Fixed	Logistic Regression	79%	100%	100%	100%
		Random Forest	81%	100%	100%	100%
	Linkage Disequilibrium	Logistic Regression	66%	95%	100%	100%
		Random Forest	67%	96%	100%	100%
**No-beneficials Larger Population**	Fixation Times	Logistic Regression	100%	100%	100%	100%
		Random Forest	100%	100%	100%	100%
	SFS	Logistic Regression	100%	100%	100%	100%
		Random Forest	100%	100%	100%	100%
	Fraction of Mutations Fixed	Logistic Regression	88%	100%	100%	100%
		Random Forest	85%	100%	100%	100%
	Linkage Disequilibrium	Logistic Regression	63%	98%	100%	100%
		Random Forest	58%	100%	100%	100%
**No-beneficials Sparse**	Fixation Times	Logistic Regression	54%	76%	98%	100%
		Random Forest	56%	74%	99%	100%
	SFS	Logistic Regression	54%	79%	100%	100%
		Random Forest	55%	88%	100%	100%
	Fraction of Mutations Fixed	Logistic Regression	61%	72%	95%	100%
		Random Forest	55%	72%	97%	100%
	Linkage Disequilibrium	Logistic Regression	57%	83%	99%	100%
		Random Forest	59%	85%	99%	100%
**Strictly Neutral**	Fixation Times	Logistic Regression	47%	49%	48%	52%
		Random Forest	49%	50%	53%	52%
	SFS	Logistic Regression	52%	55%	72%	89%
		Random Forest	48%	60%	73%	87%
	Fraction of Mutations Fixed	Logistic Regression	43%	47%	47%	53%
		Random Forest	52%	54%	56%	55%
	Linkage Disequilibrium	Logistic Regression	55%	84%	99%	100%
		Random Forest	59%	83%	97%	100%
**Population Expansion**	Fixation Times	Logistic Regression	100%	100%	100%	100%
		Random Forest	100%	100%	100%	100%
	SFS	Logistic Regression	72%	100%	100%	100%
		Random Forest	66%	98%	100%	100%
	Fraction of Mutations Fixed	Logistic Regression	100%	100%	100%	100%

		Random Forest	100%	100%	100%	100%
	Linkage Disequilibrium	Logistic Regression	56%	85%	100%	100%
		Random Forest	58%	82%	99%	100%
**Population Contraction**	Fixation Times	Logistic Regression	99%	100%	100%	100%
		Random Forest	98%	100%	100%	100%
	SFS	Logistic Regression	63%	100%	100%	100%
		Random Forest	69%	99%	100%	100%
	Fraction of Mutations Fixed	Logistic Regression	100%	100%	100%	100%
		Random Forest	100%	100%	100%	100%
	Linkage Disequilibrium	Logistic Regression	64%	92%	100%	100%
		Random Forest	64%	93%	100%	100%
**Sweep**	Fixation Times	Logistic Regression	49%	55%	59%	61%
		Random Forest	52%	54%	65%	73%
	SFS	Logistic Regression	55%	60%	72%	87%
		Random Forest	53%	63%	75%	90%
	Linkage Disequilibrium	Logistic Regression	61%	82%	99%	100%
		Random Forest	64%	86%	97%	100%
			**Q=50**	**Q=100**	**Q=150**	**Q=200**
Drosophila	Fixation Times	Logistic Regression	51%	55%	55%	53%
		Random Forest	55%	54%	51%	53%
	SFS	Logistic Regression	49%	56%	60%	60%
		Random Forest	51%	54%	61%	66%
	Fraction of Mutations Fixed	Logistic Regression	53%	55%	53%	55%
		Random Forest	56%	64%	68%	72%
	Linkage Disequilibrium	Logistic Regression	56%	53%	49%	50%
		Random Forest	50%	50%	50%	55%
***Drosophila* Stronger Selection**	Fixation Times	Logistic Regression	100%	100%	100%	100%
		Random Forest	100%	100%	100%	100%
	SFS	Logistic Regression	56%	66%	75%	77%
		Random Forest	53%	64%	71%	78%
	Fraction of Mutations Fixed	Logistic Regression	100%	100%	100%	100%
		Random Forest	100%	100%	100%	100%
	Linkage Disequilibrium	Logistic Regression	50%	49%	53%	50%
		Random Forest	55%	62%	67%	76%

Table S3. Simulation Runtimes (in Minutes) for Select Simulation Models.

Model	Q=1	Q=2	Q=5	Q=10	Q=20
**Full Model**	308	100	22	6	1.4
**No-beneficials Larger Population**	5645	1929	433	109	20.5
**Strictly Neutral**	493	173	48	18	7.7
**Population Expansion**	408	122	24	6	1.6
**Population Contraction**	208	57	12	3	0.7
	**Q=20**	**Q=50**	**Q=100**	**Q=150**	**Q=200**
Drosophila	1458	311	78	32	23
***Drosophila* Stronger Selection**	1383	201	43	26	12
