# Supplementary material for: Population size rescaling significantly biases outcomes of forward-in-time population genetic simulations
Source: bioRxiv. 2024 Apr 18:2024.04.07.588318. Preprint. [Version 2] doi: 10.1101/2024.04.07.588318 (PMC11030438; doi:10.1101/2024.04.07.588318)
Supplement: Supplementary file 1 [file media-1.pdf]

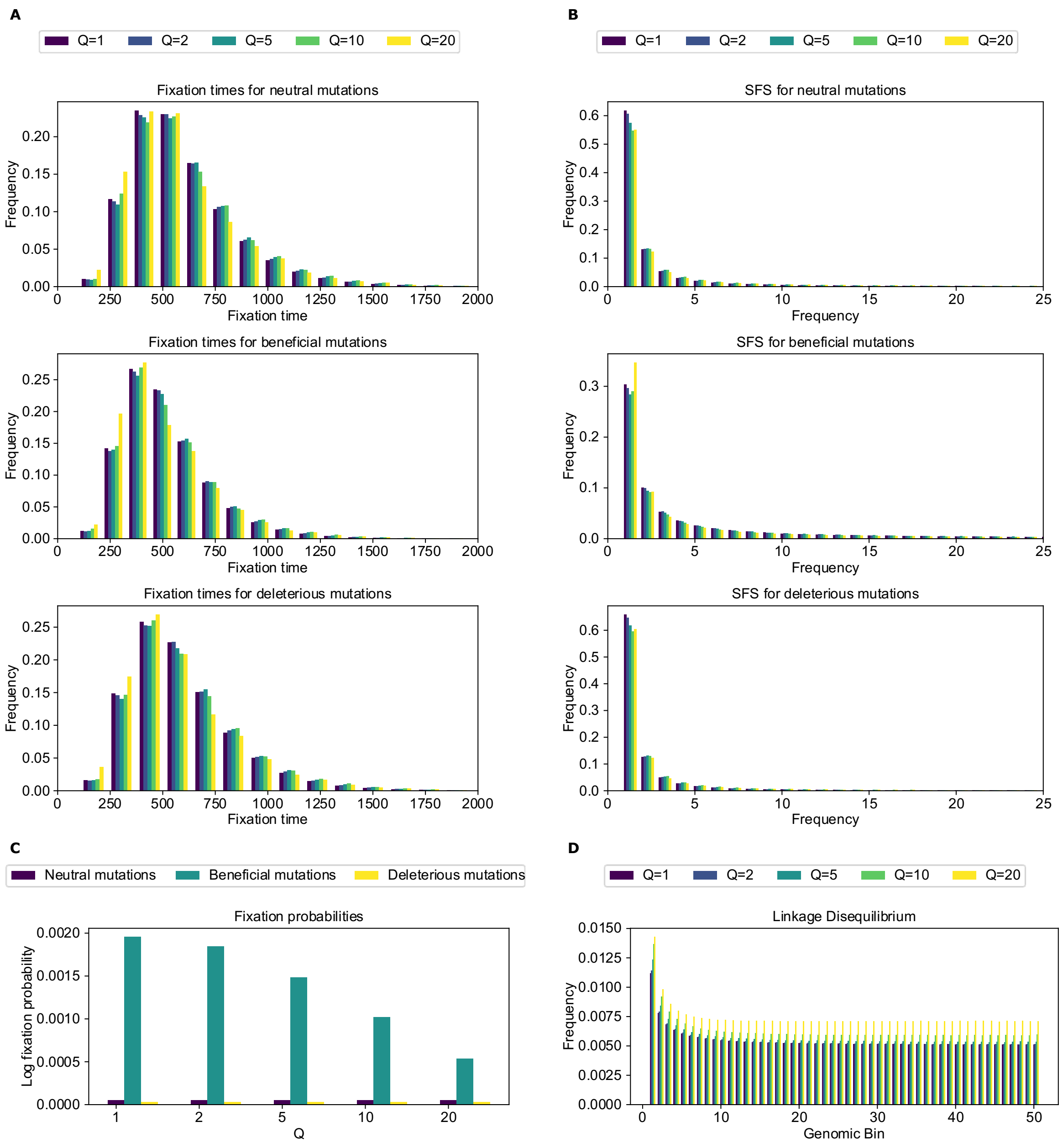

Figure S1. Simulation outcomes for the base model.
(A) Fixation time distributions for all mutation types. **(B)** Site frequency spectra for all mutation types. **(C)** Fixation probabilities for all mutation types. **(D)** Linkage disequilibrium across the chromosome for 50 genomic bins, as measured by r^2. Each outcome was averaged across all replicates. For fixation times, SFS, and LD, this was achieved by averaging each distribution bin across the replicates.

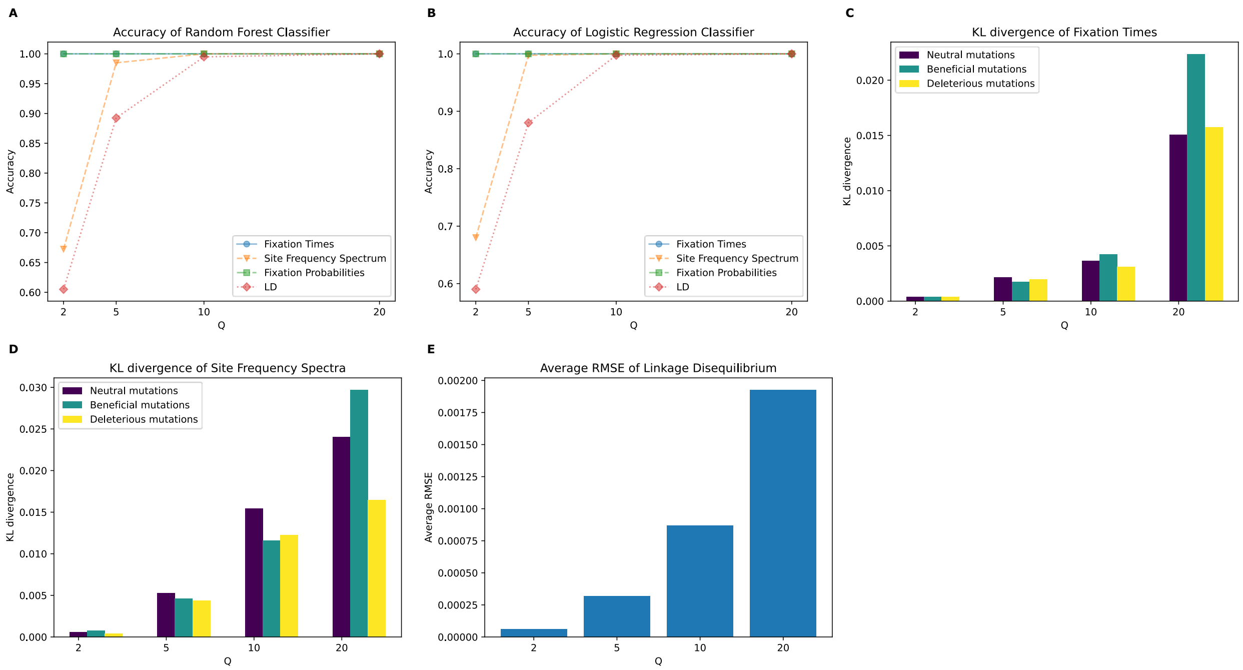

Figure S2. Metrics of the effect of scaling for the full model.
(A) Accuracy of the random forest classification using features subsets for each scaling factor. **(B)** Accuracy of the logistic regression classification using feature subsets for each scaling factor. **(C)** KL divergence of fixation time distributions for all mutation types. **(D)** KL divergence for the SFS for all mutation types. (**E)** Average RMSE of LD. Classifier accuracy represents the proportion of replicates correctly identified by the model as scaled and unscaled using the feature subsets for each Q.

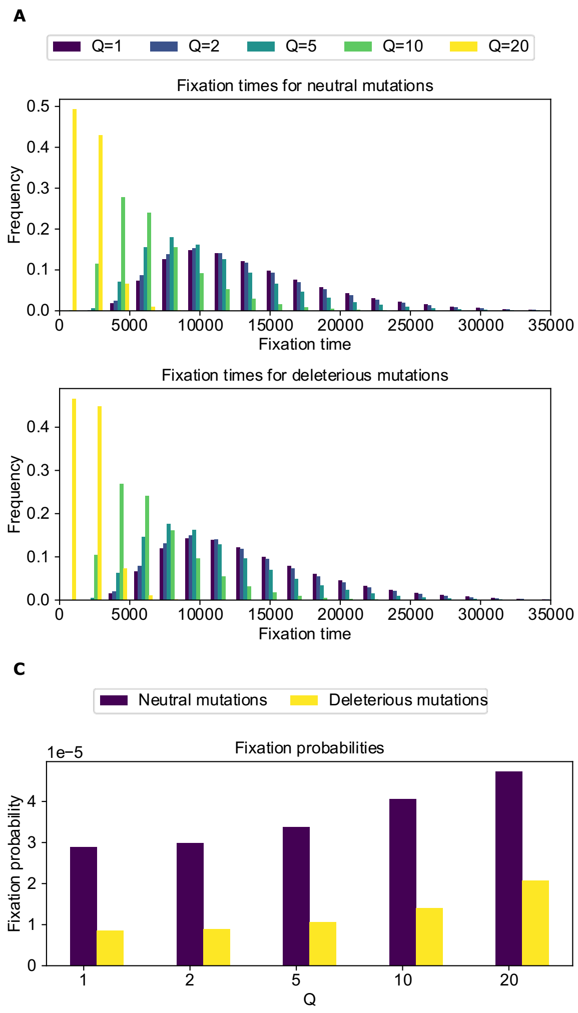

Figure S3. **Simulation outcomes for the no-beneficials model.**
(A) Fixation time distributions for all mutation types. **(B)** Site frequency spectra for all mutation types. **(C)** Fixation probabilities for all mutation types. **(D)** Linkage disequilibrium across the chromosome for 50 genomic bins, as measured by r^2.

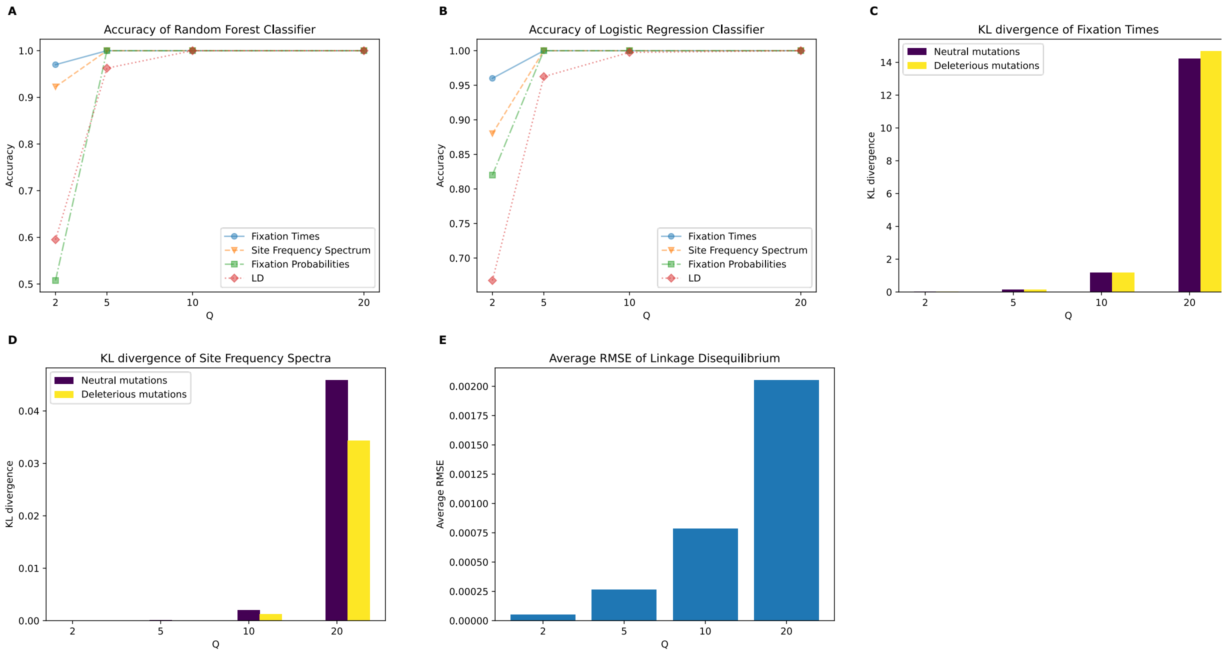

Figure S4. Metrics of the effect of scaling for the no-beneficials model.
(A) Accuracy of the random forest classification using features subsets for each scaling factor. **(B)** Accuracy of the logistic regression classification using feature subsets for each scaling factor. **(C)** KL divergence of fixation time distributions for all mutation types. **(D)** KL divergence for the SFS for all mutation types. **(E)** Average RMSE of LD.

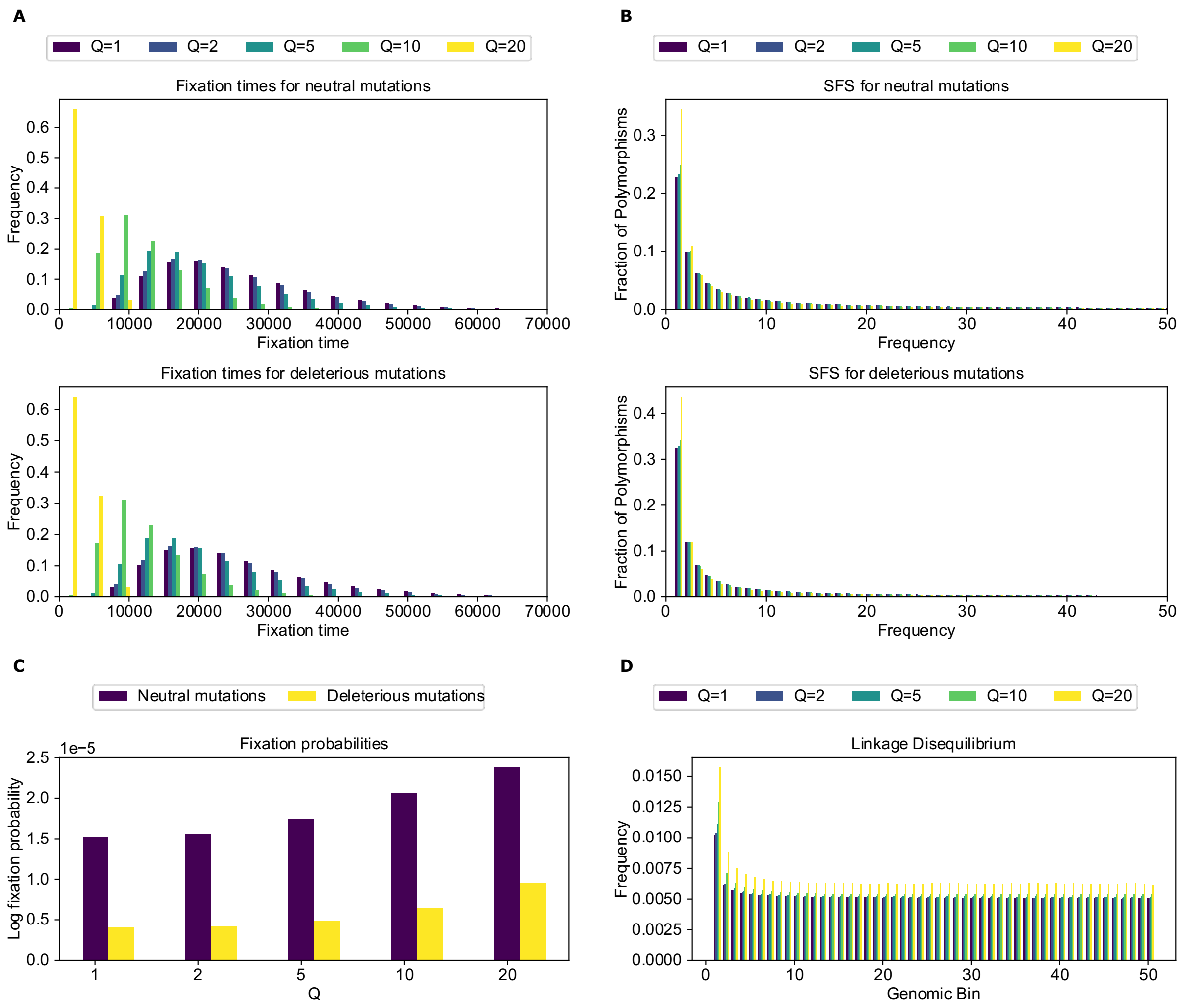

Figure S5. Simulation outcomes for the no-beneficials model with a larger initial population size. **(A)** Fixation time distributions for all mutation types. **(B)** Site frequency spectra for all mutation types. **(C)** Fixation probabilities for all mutation types. **(D)** Linkage disequilibrium across the chromosome for 50 genomic bins, as measured by r^2.

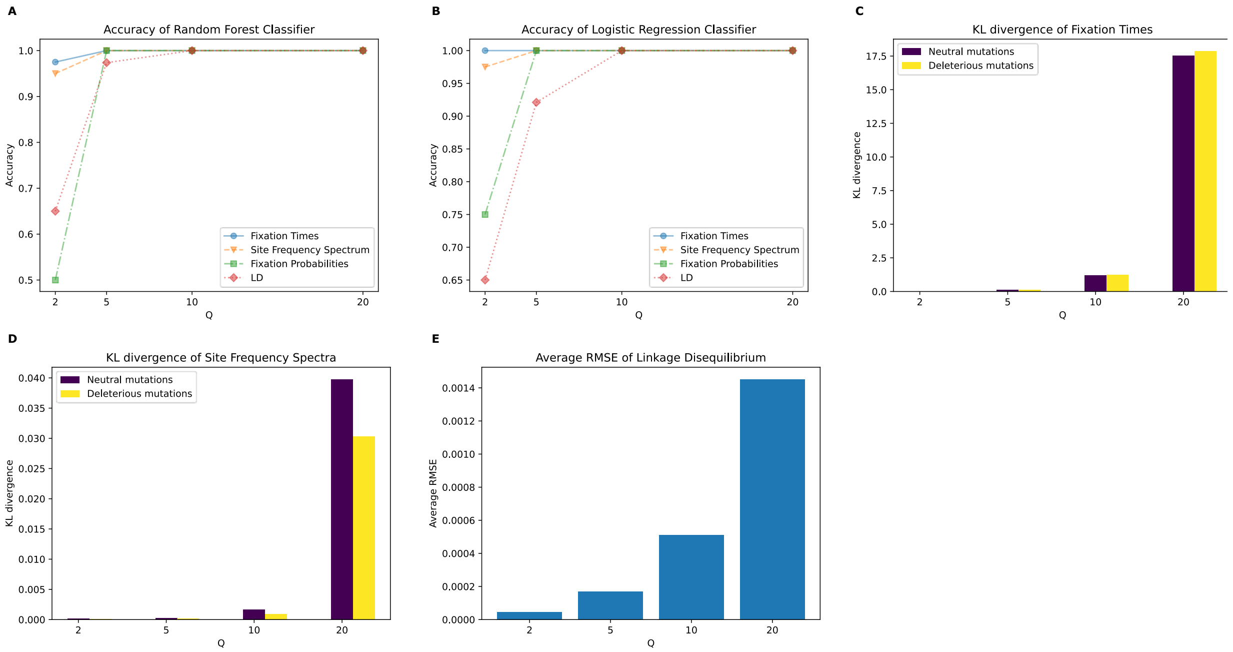

Figure S6. Metrics of the effect of scaling for the no-beneficials model with a larger initial population size. (A) Accuracy of the random forest classification using features subsets for each scaling factor. **(B)** Accuracy of the logistic regression classification using feature subsets for each scaling factor. **(C)** KL divergence of fixation time distributions for all mutation types. **(D)** KL divergence for the SFS for all mutation types. **(E)** Average RMSE of LD.

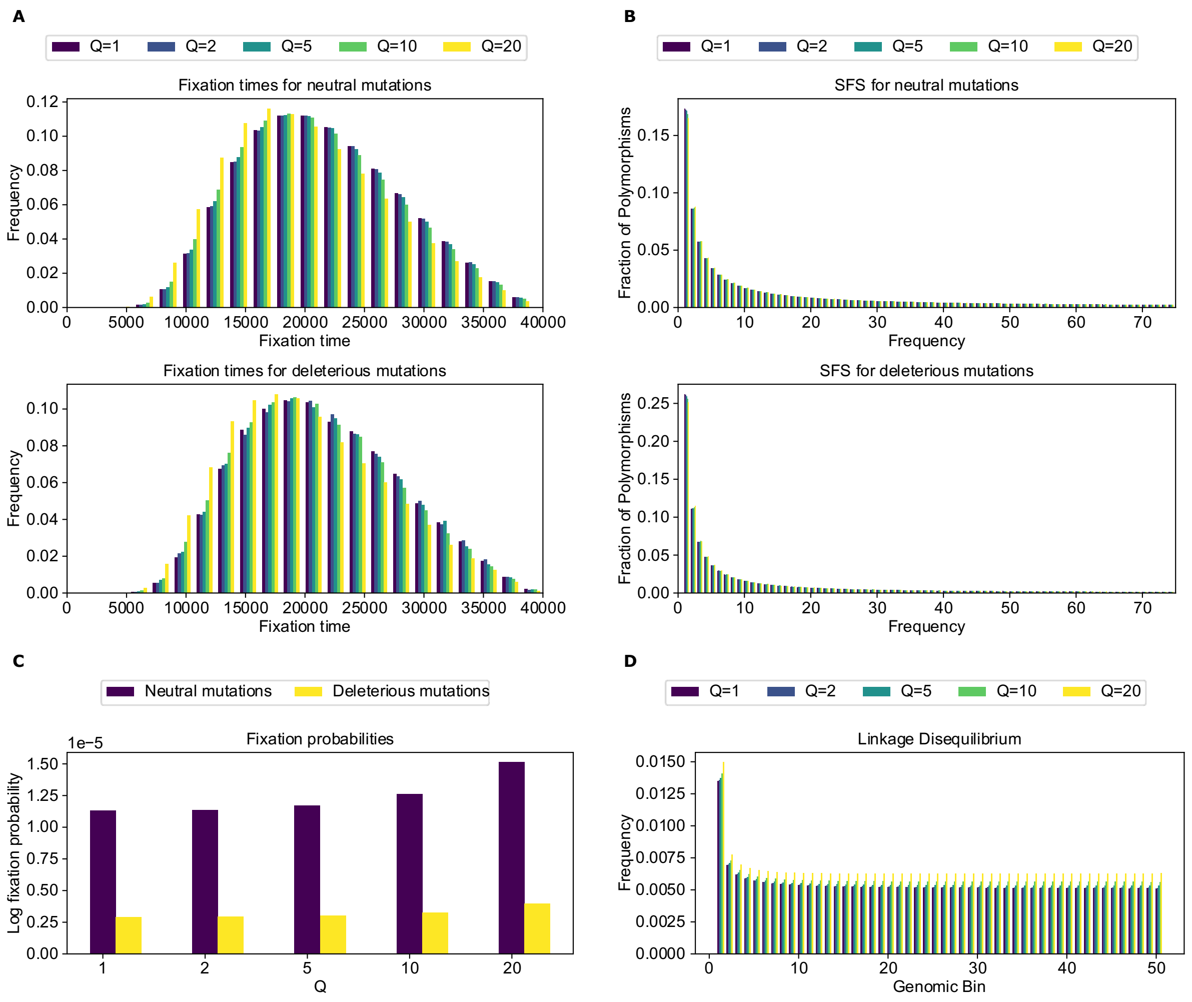

Figure S7. Simulation outcomes for the sparse genome deleterious-only model.
(A) Fixation time distributions for all mutation types. **(B)** Site frequency spectra for all mutation types. **(C)** Fixation probabilities for all mutation types. **(D)** Linkage disequilibrium across the chromosome for 50 genomic bins, as measured by r^2.

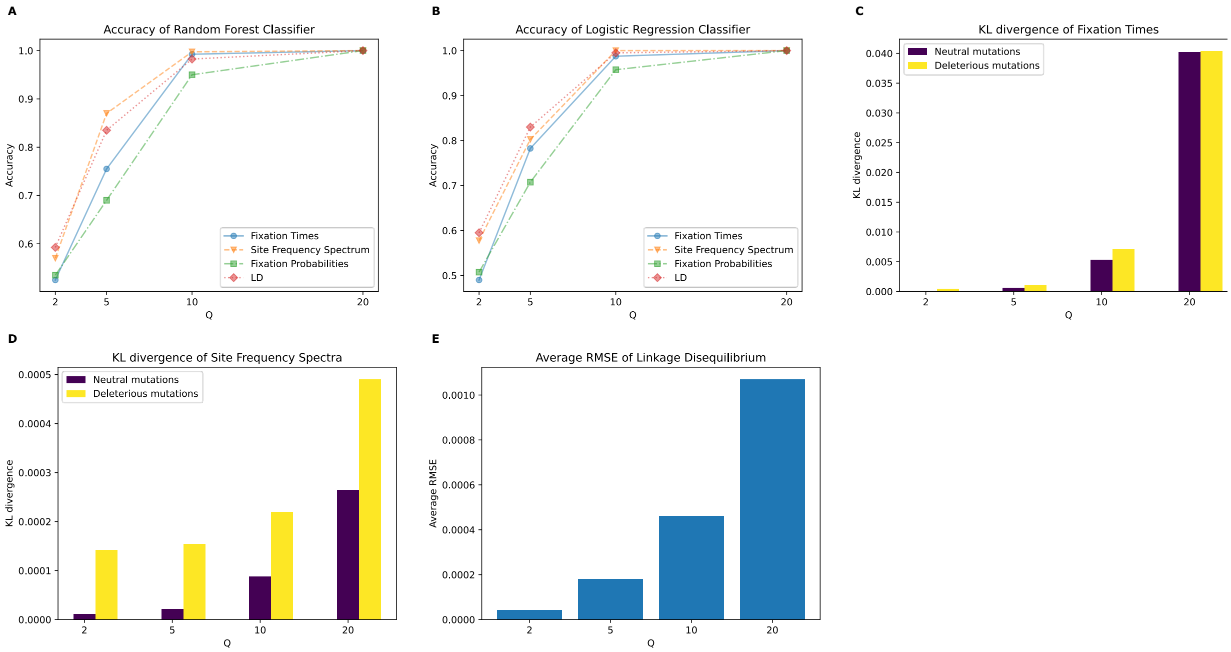

Figure S8. Metrics of the effect of scaling for the sparse genome deleterious-only model.
(A) Accuracy of the random forest classification using features subsets for each scaling factor. **(B)** Accuracy of the logistic regression classification using feature subsets for each scaling factor. **(C)** KL divergence of fixation time distributions for all mutation types. **(D)** KL divergence for the SFS for all mutation types. **(E)** Average RMSE of LD.

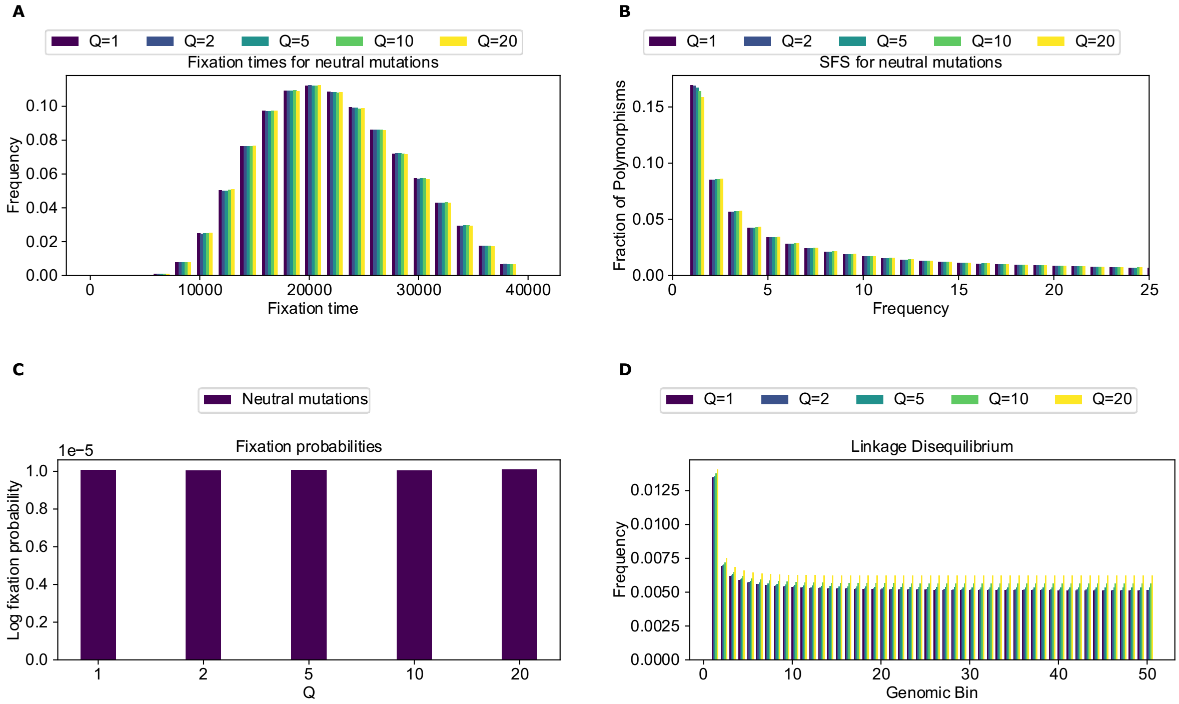

Figure S9. Simulation outcomes for the neutral-only model.
(A) Fixation time distributions for neutral mutations. **(B)** Site frequency spectra for neutral mutations. **(C)** Fixation probabilities for neutral mutations. **(D)** Linkage disequilibrium across the chromosome for 50 genomic bins, as measured by r^2.

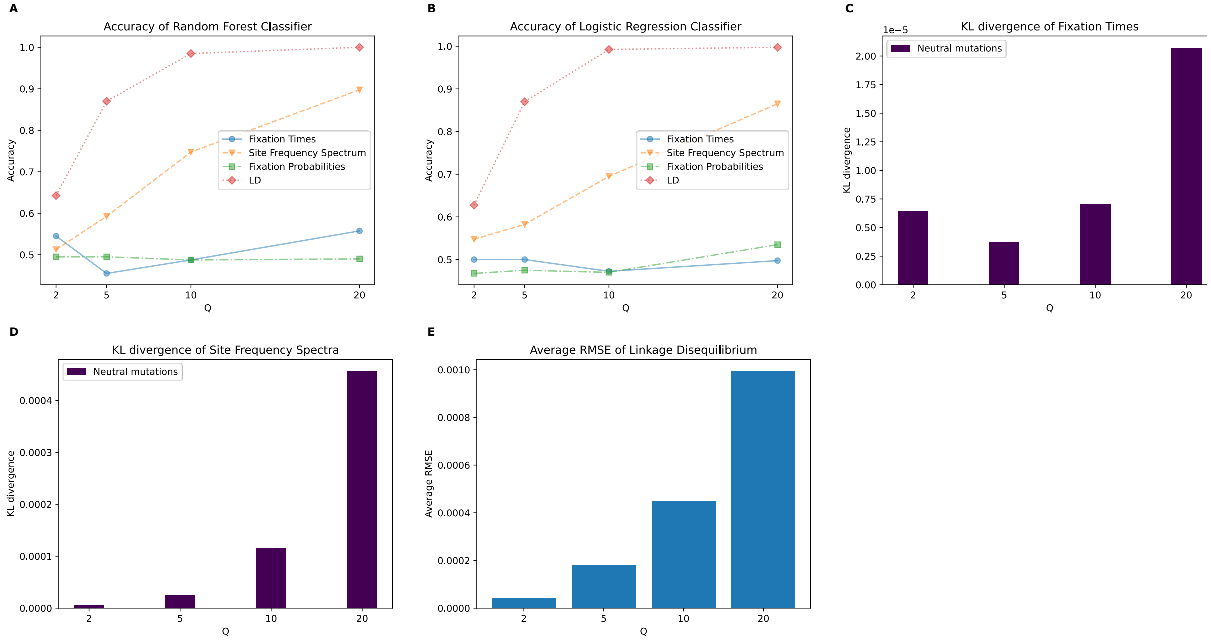

Figure S10. Metrics of the effect of scaling for the neutral-only model.
(A) Accuracy of the random forest classification using features subsets for each scaling factor. **(B)** Accuracy of the logistic regression classification using feature subsets for each scaling factor. **(C)** KL divergence of fixation time distributions for all mutation types. **(D)** KL divergence for the SFS for all mutation types. **(E)** Average RMSE of LD.

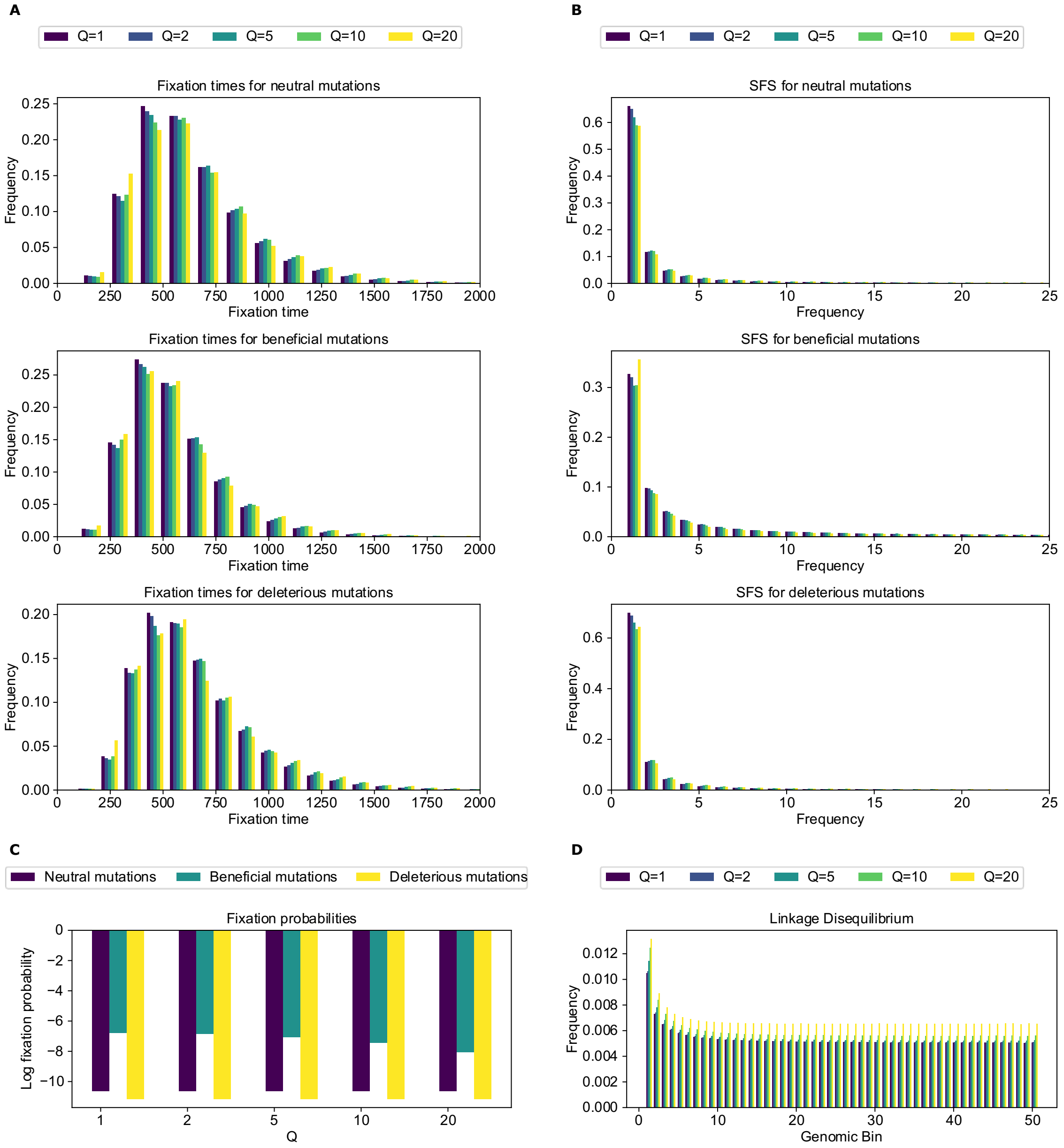

Figure S11. Simulation outcomes for the population expansion model.
(A) Fixation time distributions for all mutation types. **(B)** Site frequency spectra for all mutation types. **(C)** Fixation probabilities for all mutation types. **(D)** Linkage disequilibrium across the chromosome for 50 genomic bins, as measured by r^2.

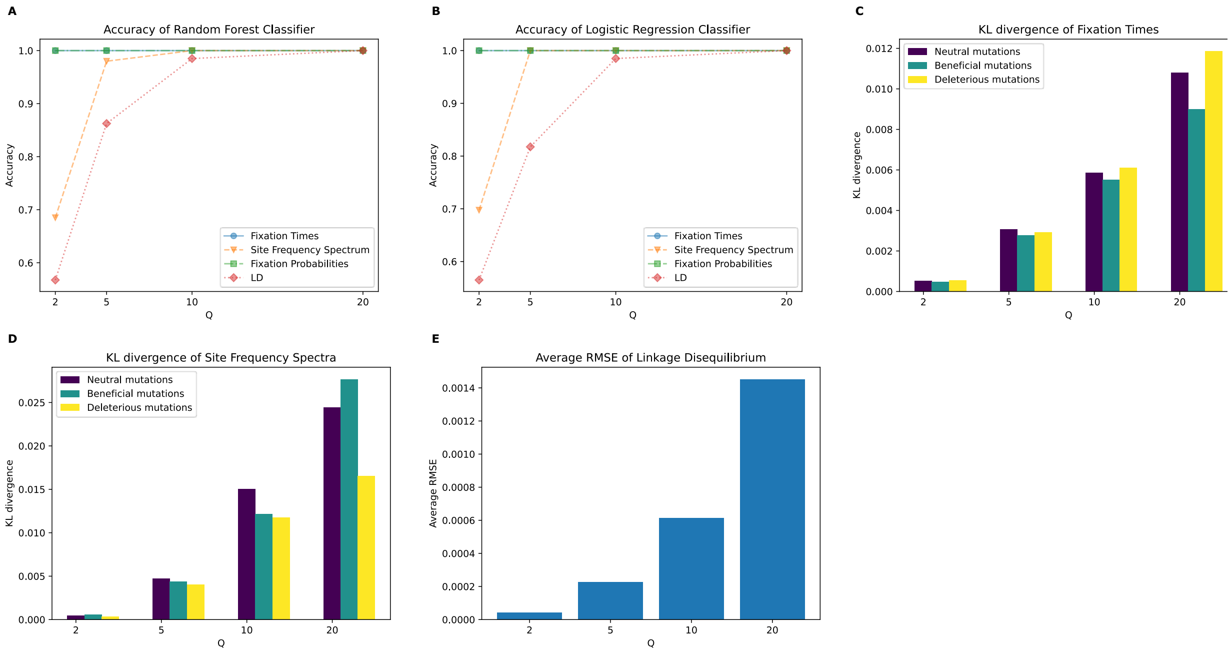

Figure S12. Metrics of the effect of scaling for the population expansion model.
(A) Accuracy of the random forest classification using features subsets for each scaling factor. **(B)** Accuracy of the logistic regression classification using feature subsets for each scaling factor. **(C)** KL divergence of fixation time distributions for all mutation types. **(D)** KL divergence for the SFS for all mutation types. **(E)** Average RMSE of LD.

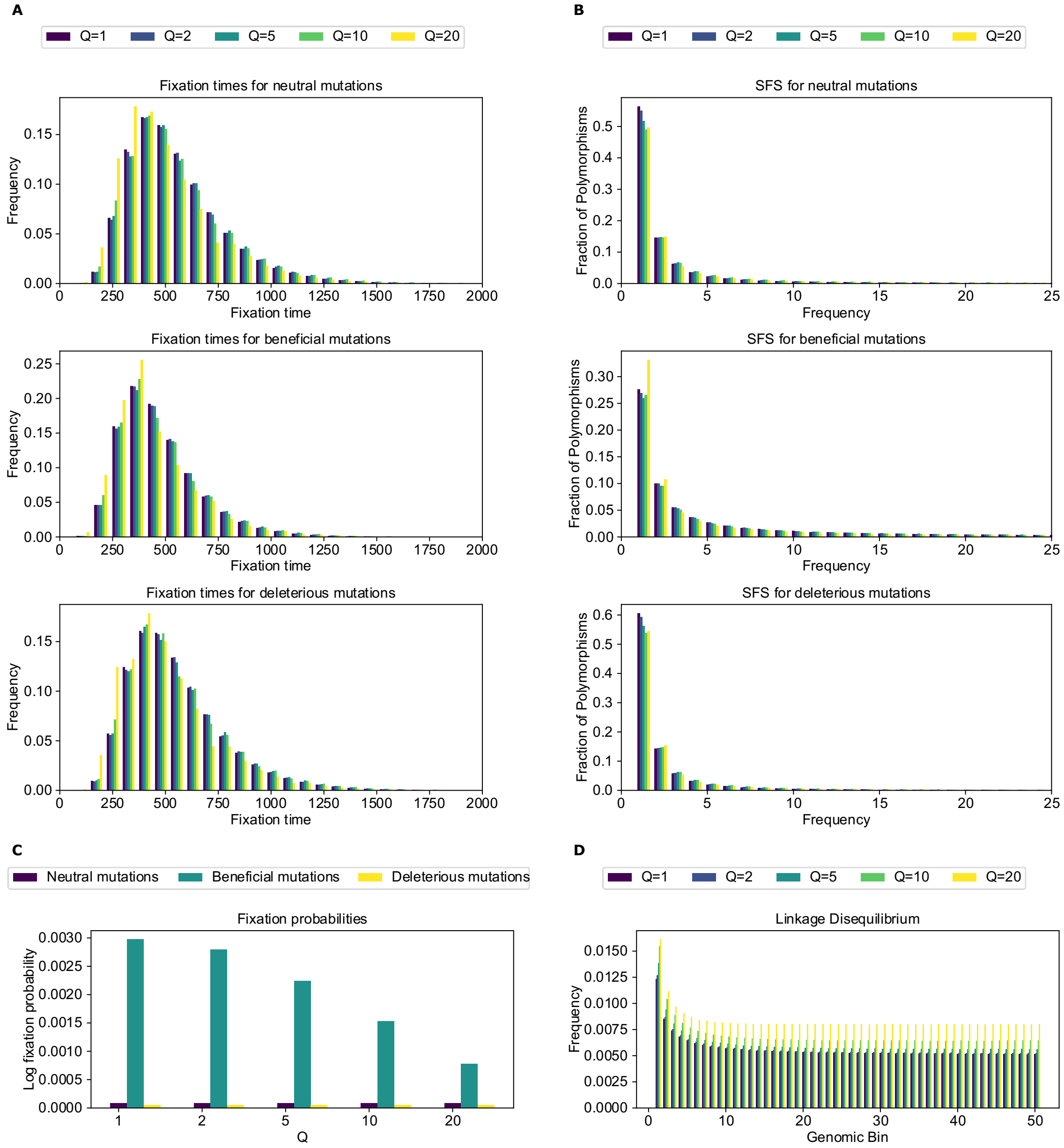

Figure S13. Simulation outcomes for the population contraction model.
(A) Fixation time distributions for all mutation types. **(B)** Site frequency spectra for all mutation types. **(C)** Fixation probabilities for all mutation types. **(D)** Linkage disequilibrium across the chromosome for 50 genomic bins, as measured by r^2.

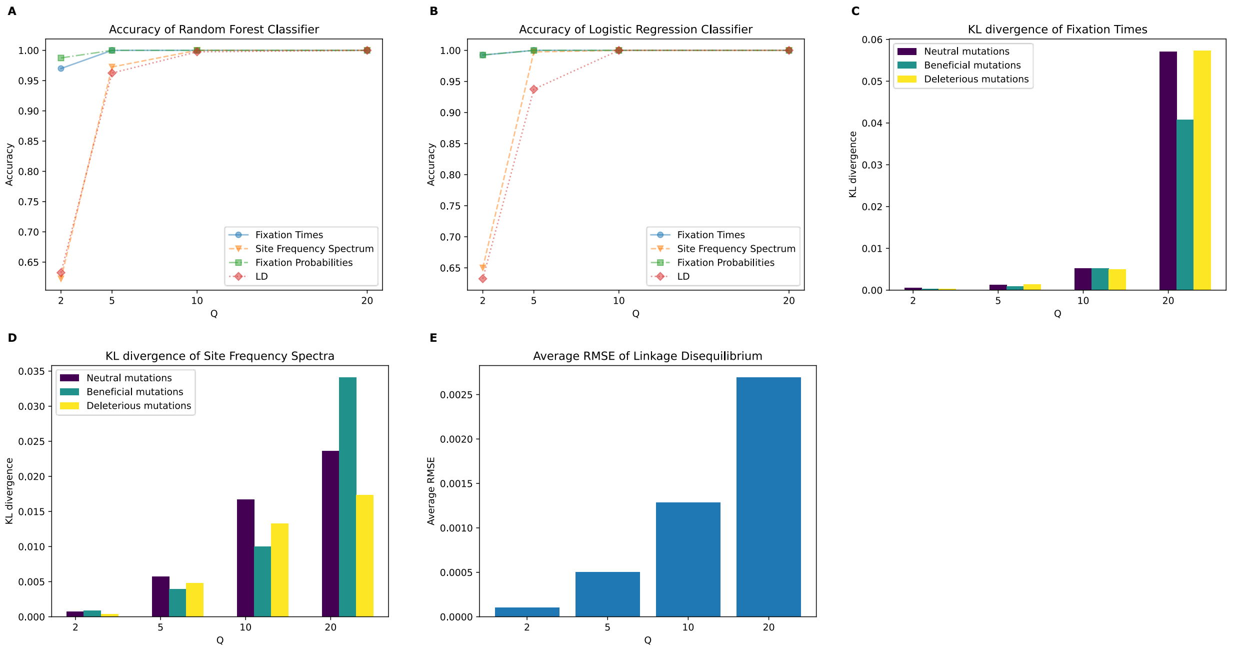

Figure S14. Metrics of the effect of scaling for the population contraction model.
(A) Accuracy of the random forest classification using features subsets for each scaling factor. **(B)** Accuracy of the logistic regression classification using feature subsets for each scaling factor. **(C)** KL divergence of fixation time distributions for all mutation types. **(D)** KL divergence for the SFS for all mutation types. **(E)** Average RMSE of LD.

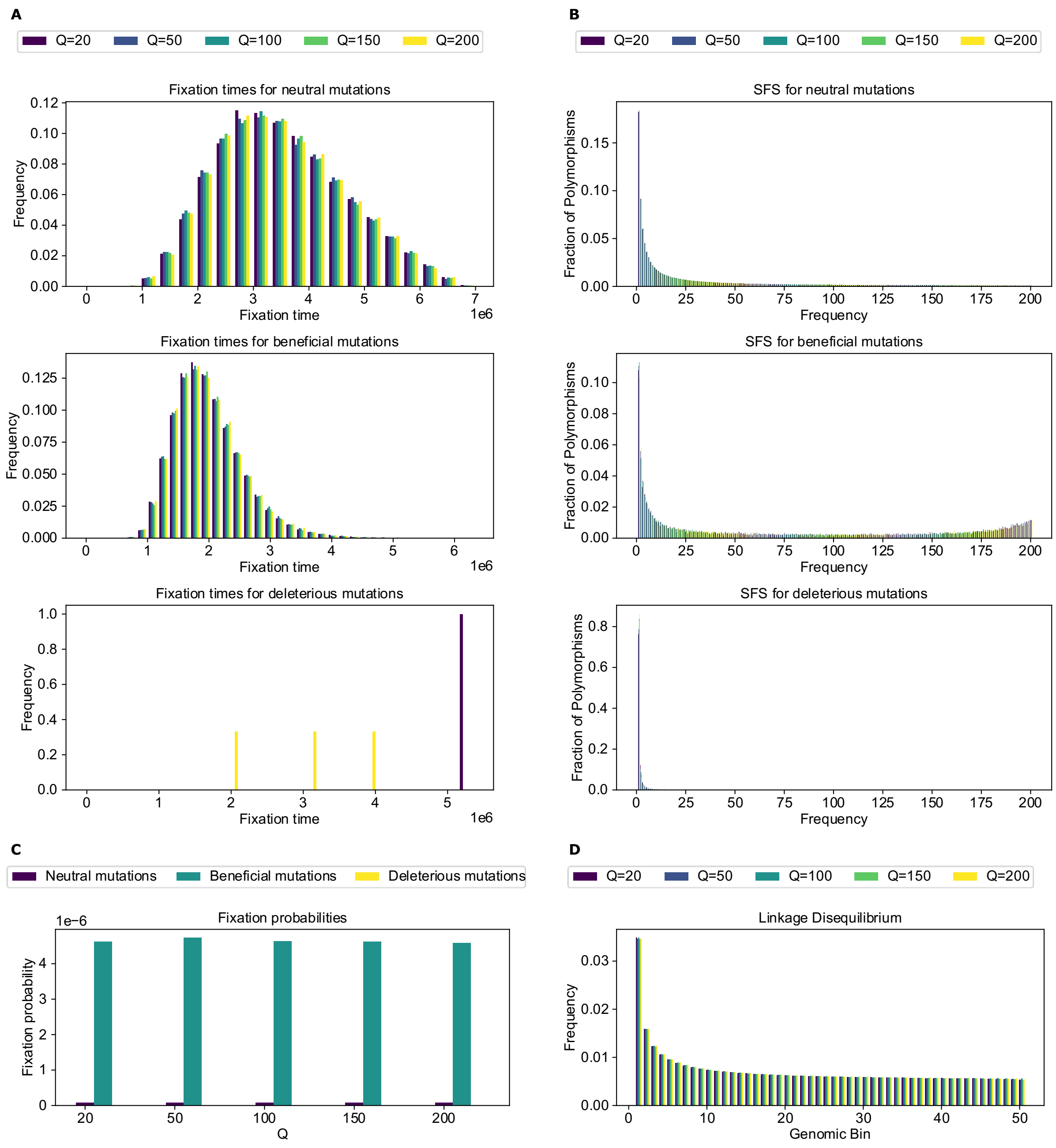

Figure S15. Simulation outcomes for the *Drosophila* model.
(A) Fixation time distributions for all mutation types. **(B)** Site frequency spectra for all mutation types. **(C)** Fixation probabilities for all mutation types. **(D)** Linkage disequilibrium across the chromosome for 50 genomic bins, as measured by r^2.

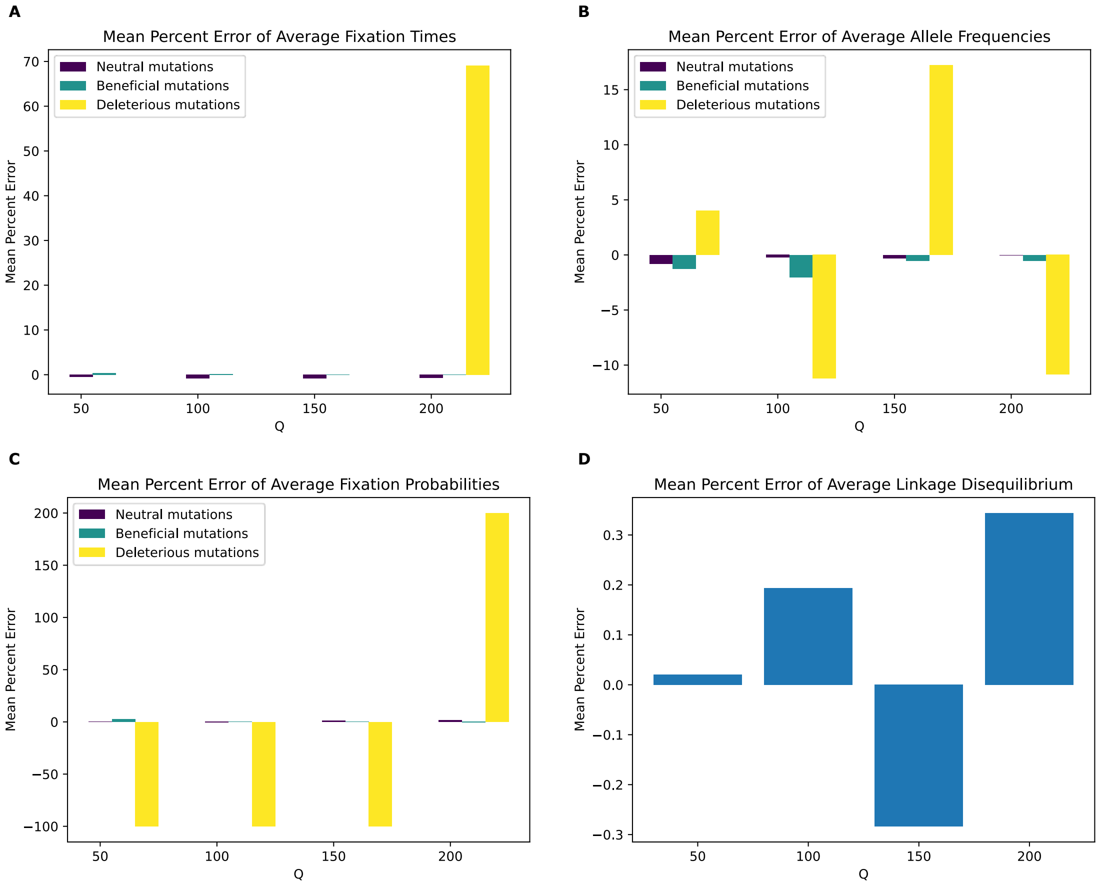

Figure S16. Mean percentage error of various statistics for *Drosophila* model by values of *Q*.
(A) Mean percentage error for average mutation fixation times. **(B)** Mean percentage error for average allele frequencies. **(C)** Mean percentage error for average fixation probabilities. **(D)** Mean percentage error for average linkage disequilibrium as measured by the values of r^2.

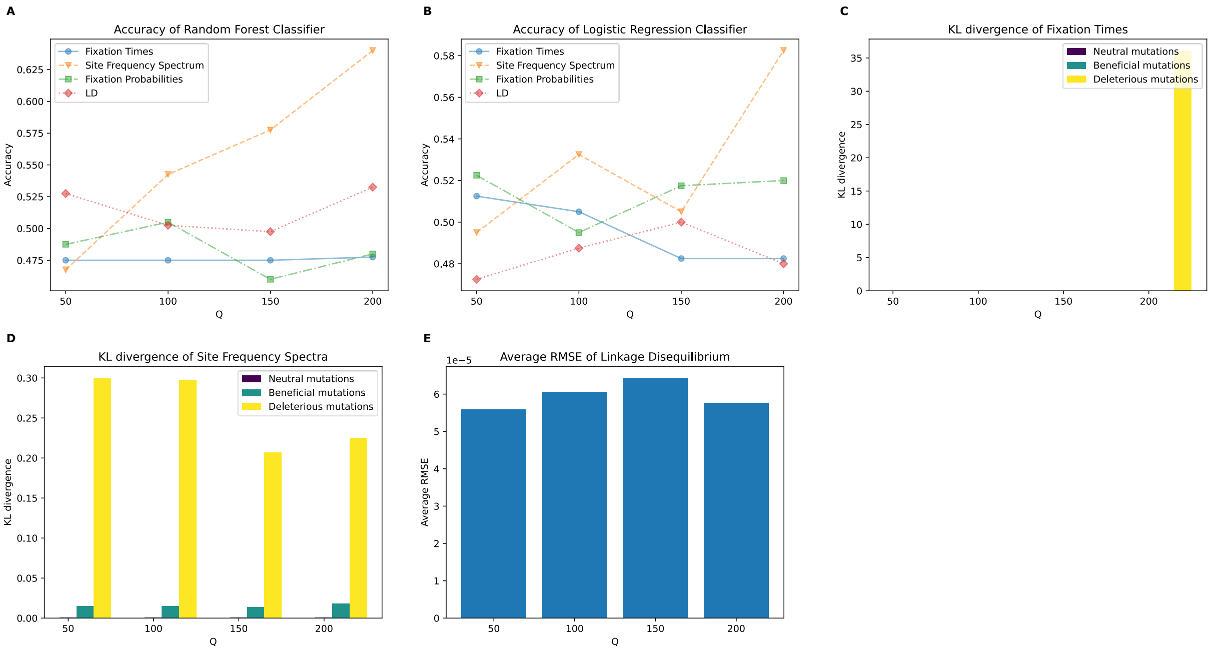

Figure S17. Metrics of the effect of scaling for the *Drosophila* model.
(A) Accuracy of the random forest classification using features subsets for each scaling factor. **(B)** Accuracy of the logistic regression classification using feature subsets for each scaling factor. **(C)** KL divergence of fixation time distributions for all mutation types. **(D)** KL divergence for the SFS for all mutation types. **(E)** Average RMSE of LD.

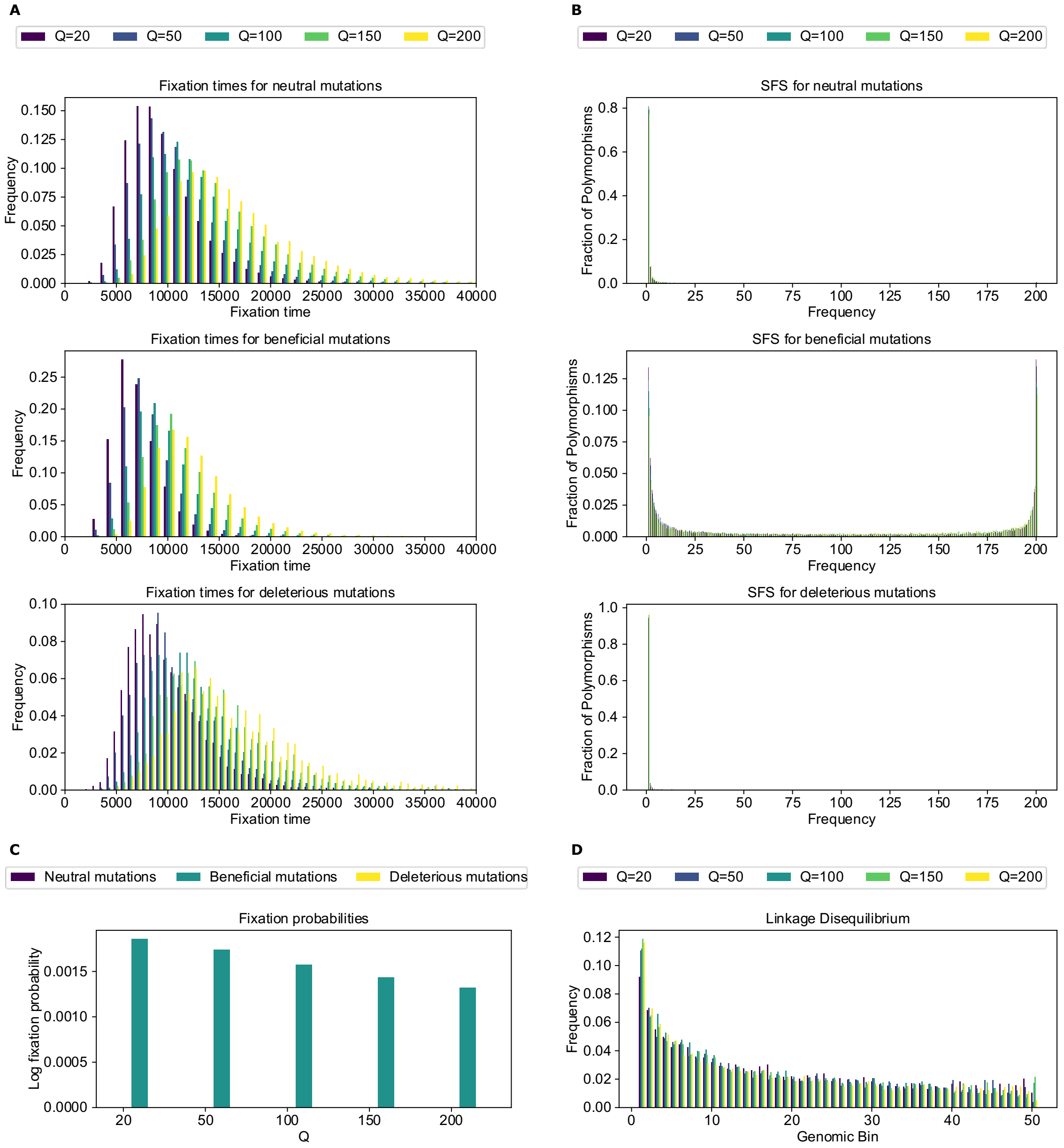

Figure S18. Simulation outcomes for the *Drosophila* higher selection model.
(A) Fixation time distributions for all mutation types. **(B)** Site frequency spectra for all mutation types. **(C)** Fixation probabilities for all mutation types. **(D)** Linkage disequilibrium across the chromosome for 50 genomic bins, as measured by r^2.

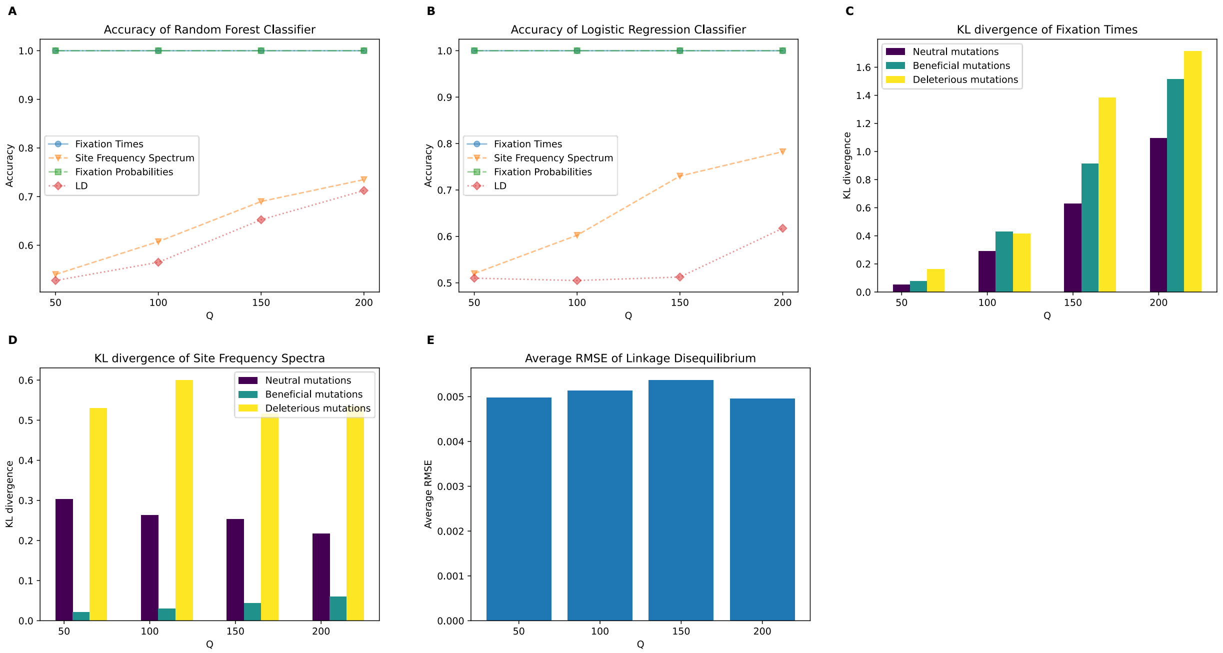

Figure S19. Metrics of the effect of scaling for the *Drosophila* higher selection model.
(A) Accuracy of the random forest classification using features subsets for each scaling factor. **(B)** Accuracy of the logistic regression classification using feature subsets for each scaling factor. **(C)** KL divergence of fixation time distributions for all mutation types. **(D)** KL divergence for the SFS for all mutation types. **(E)** Average RMSE of LD.

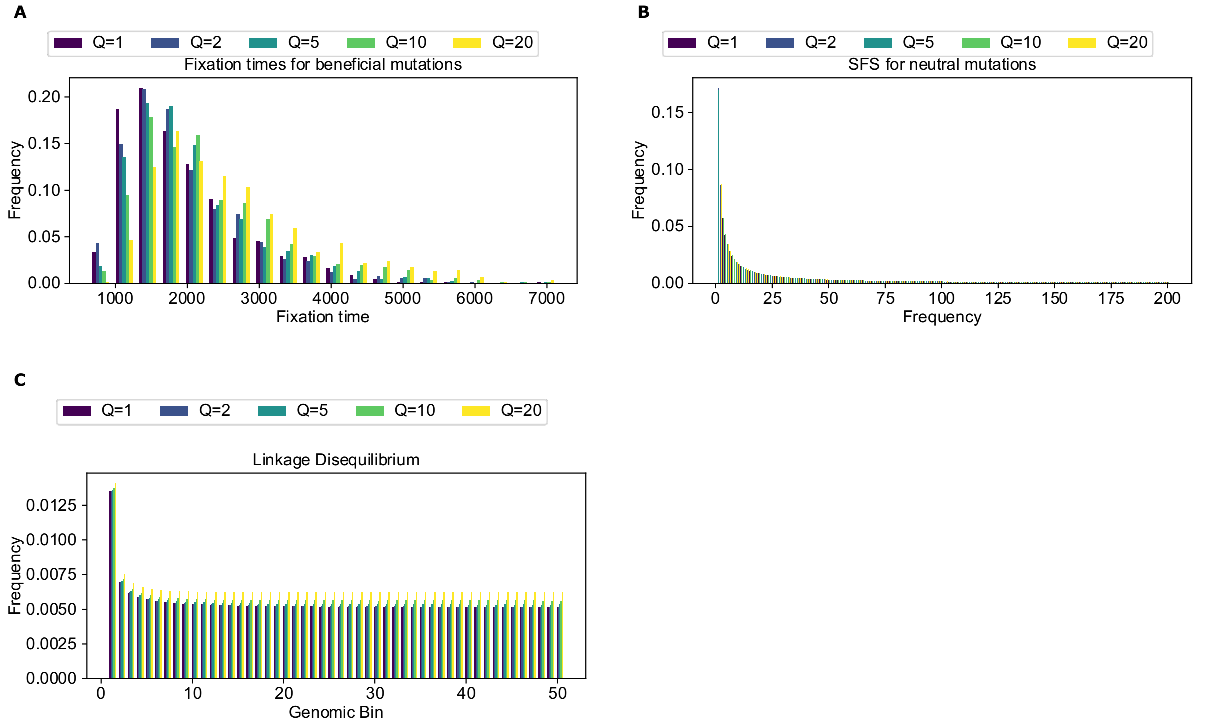

Figure S20. Simulation outcomes for the sweep model.
(A) Fixation time distributions for beneficial mutations. **(B)** Site frequency spectrum neutral mutations. **(C)** Linkage disequilibrium across the chromosome for 50 genomic bins, as measured by r^2.

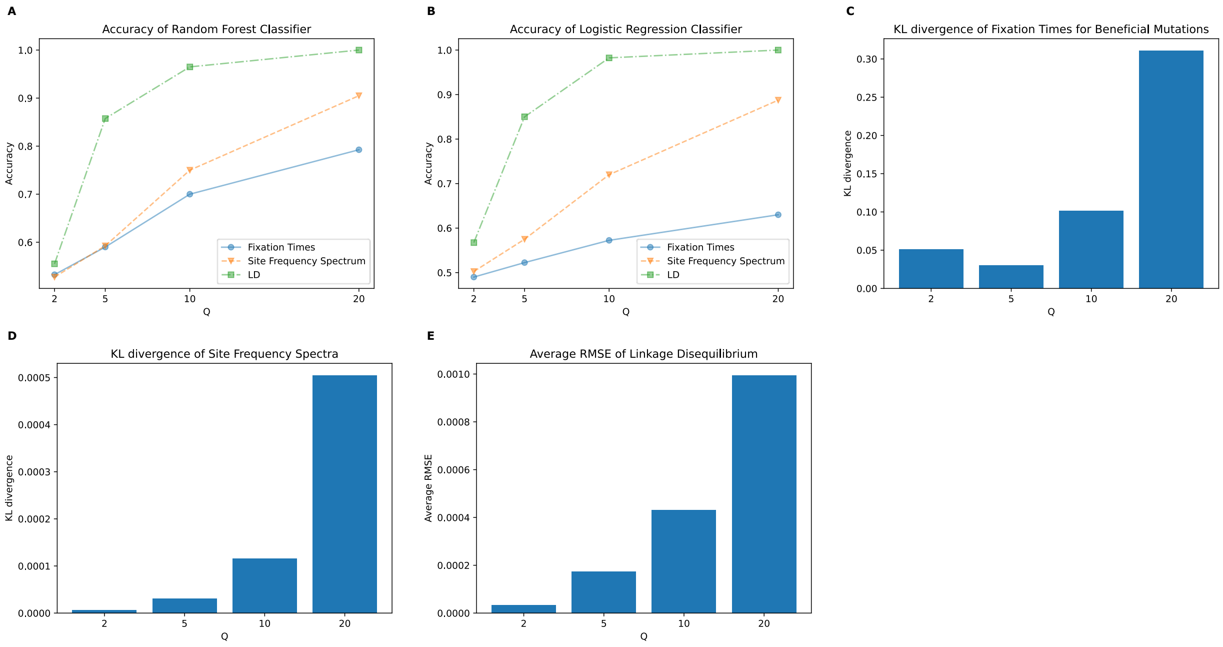

Figure S21. Metrics of the effect of scaling for the sweep model.
(A) Accuracy of the random forest classification using features subsets for each scaling factor. **(B)** Accuracy of the logistic regression classification using feature subsets for each scaling factor. **(C)** KL divergence of fixation time distributions for beneficial mutations. **(D)** KL divergence for the SFS for neutral. **(E)** Average RMSE of LD.

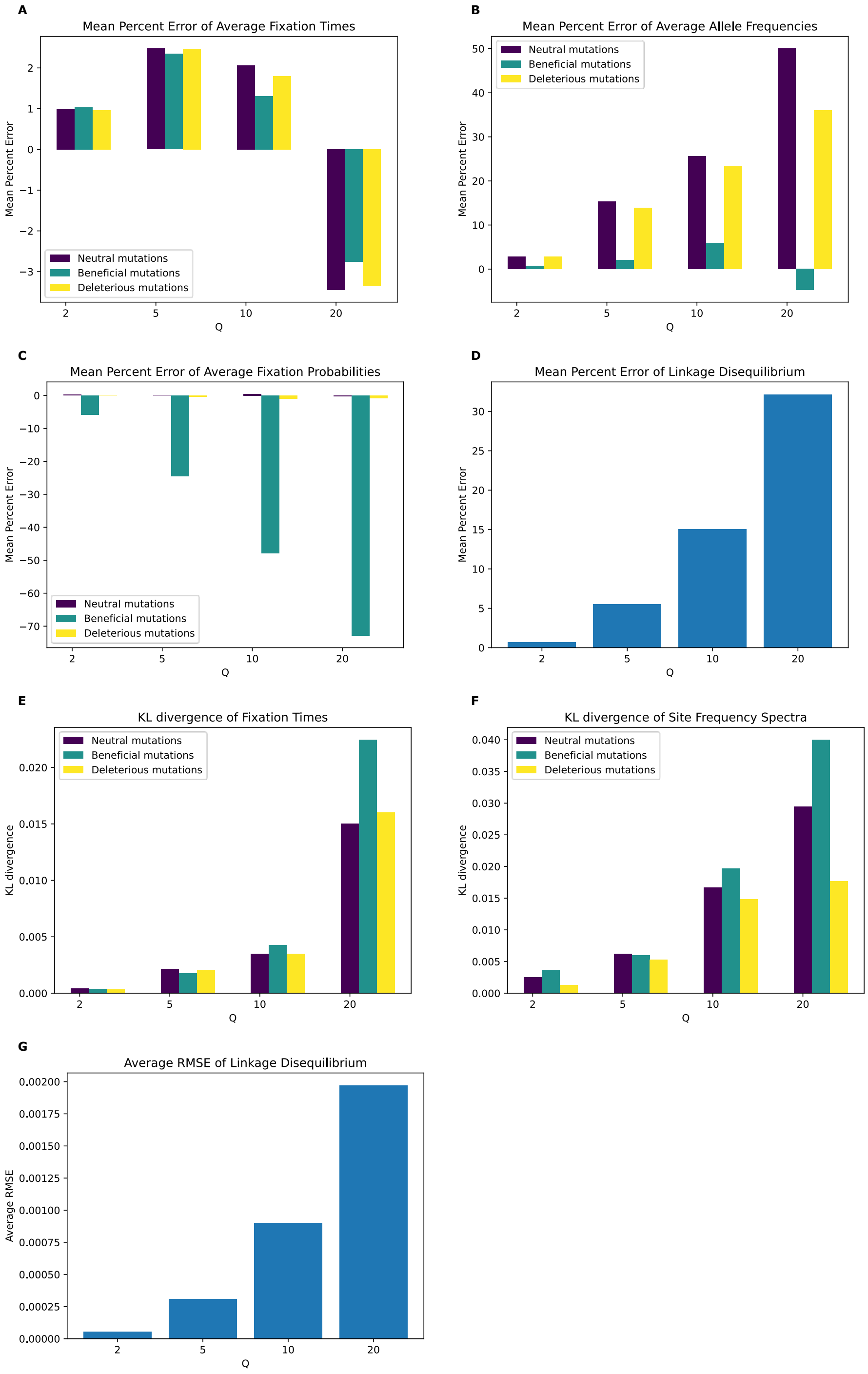

Figure S22. Measures of deviation for the full model using only 100 randomly-selected replicates of the full model.

(A) Mean percentage error of average fixation times for all mutation types. **(B)** Mean percentage error of average allele frequencies for all mutation types. **(C)** Mean percentage error of average fixation probabilities for all mutation types. **(D)** Mean percentage error of average LD as measured by r^2. **(E)** KL Divergence of fixation times for all mutation types. **(F)** KL divergence of the SFS for all mutation types. **(G)** Average RMSE of LD as measured by r^2 across 50 genomic bins.

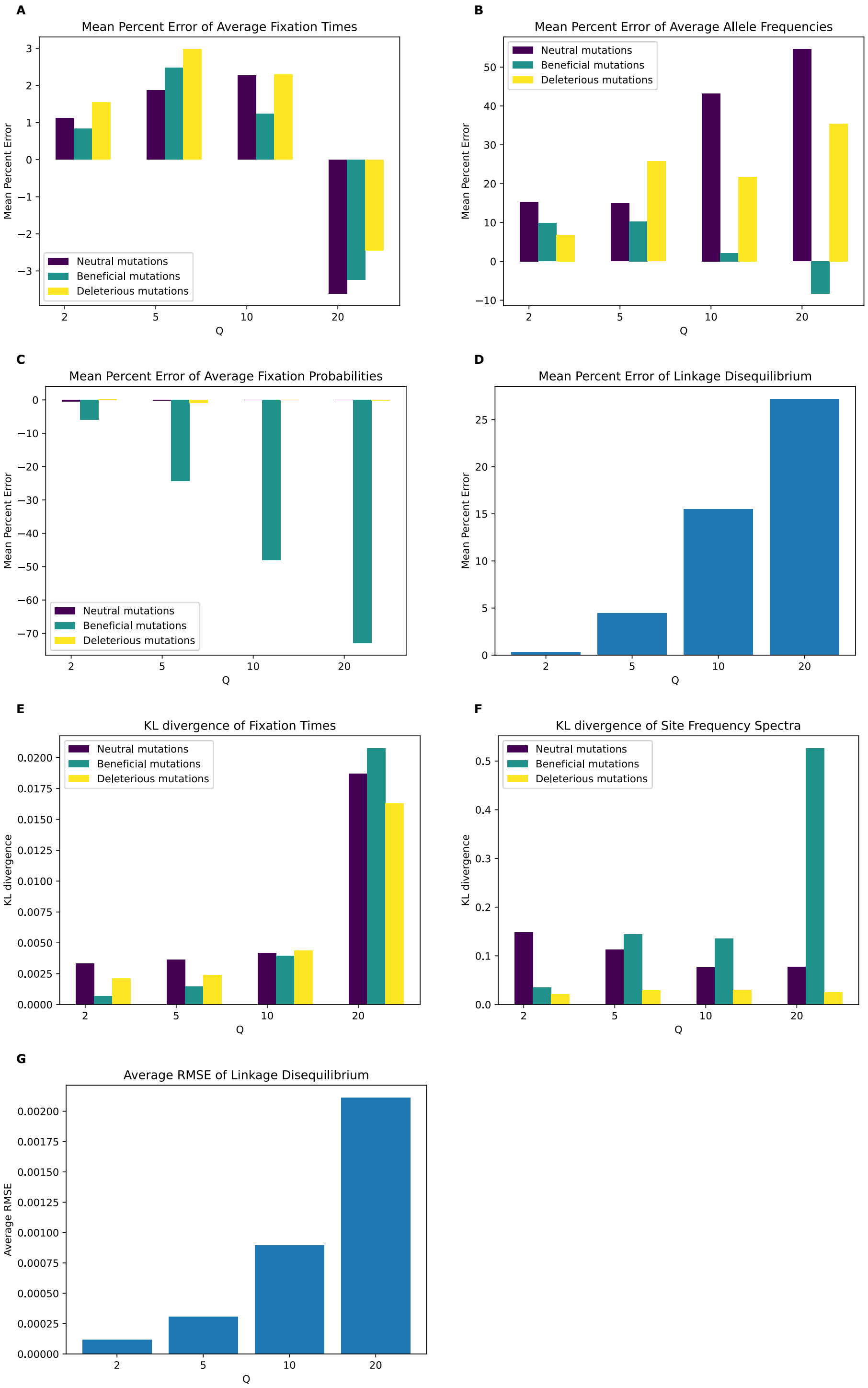

Figure S23. Measures of deviation for the full model using only 10 randomly-selected replicates of the full model.

(A) Mean percentage error of average fixation times for all mutation types. **(B)** Mean percentage error of average allele frequencies for all mutation types. **(C)** Mean percentage error of average fixation probabilities for all mutation types. **(D)** Mean percentage error of average LD as measured by r^2. **(E)** KL Divergence of fixation times for all mutation types. **(F)** KL divergence of the SFS for all mutation types. **(G)** Average RMSE of LD as measured by r^2 across 50 genomic bins.

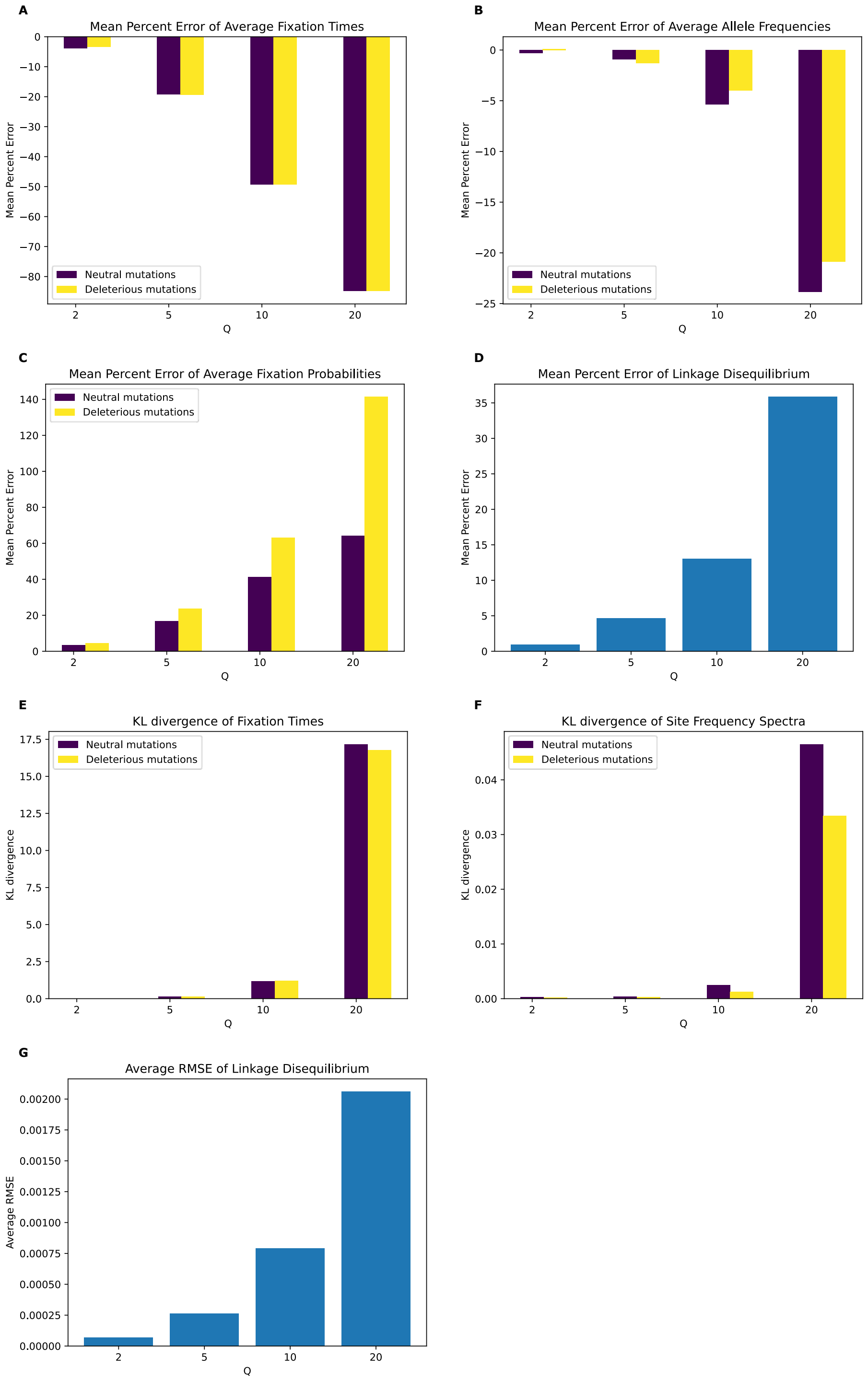

Figure S24. Measures of deviation for the no-beneficials model using only 100 randomly-selected replicates of the full model.
(A) Mean percentage error of average fixation times for all mutation types. **(B)** Mean percentage error of average allele frequencies for all mutation types. **(C)** Mean percentage error of average fixation probabilities for all mutation types. **(D)** Mean percentage error of average LD as measured by r^2. **(E)** KL Divergence of fixation times for all mutation types. **(F)** KL divergence of the SFS for all mutation types. **(G)** Average RMSE of LD as measured by r^2 across 50 genomic bins.

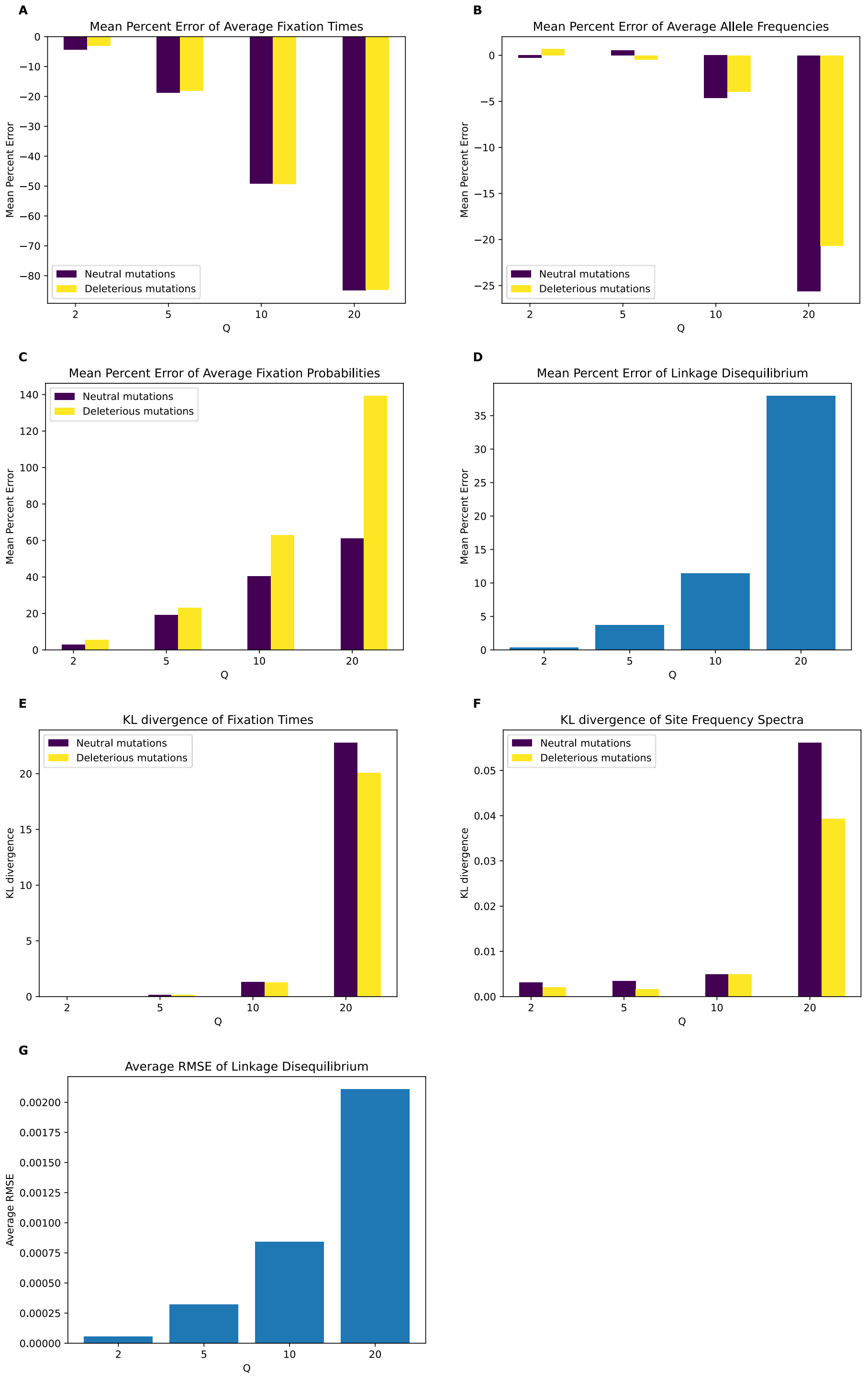

Figure S25. Measures of deviation for the no-beneficials model using only 10 randomly-selected replicates of the full model.

(A) Mean percentage error of average fixation times for all mutation types. **(B)** Mean percentage error of average allele frequencies for all mutation types. **(C)** Mean percentage error of average fixation probabilities for all mutation types. **(D)** Mean percentage error of average LD as measured by r^2. **(E)** KL Divergence of fixation times for all mutation types. **(F)** KL divergence of the SFS for all mutation types. **(G)** Average RMSE of LD as measured by r^2 across 50 genomic bins.

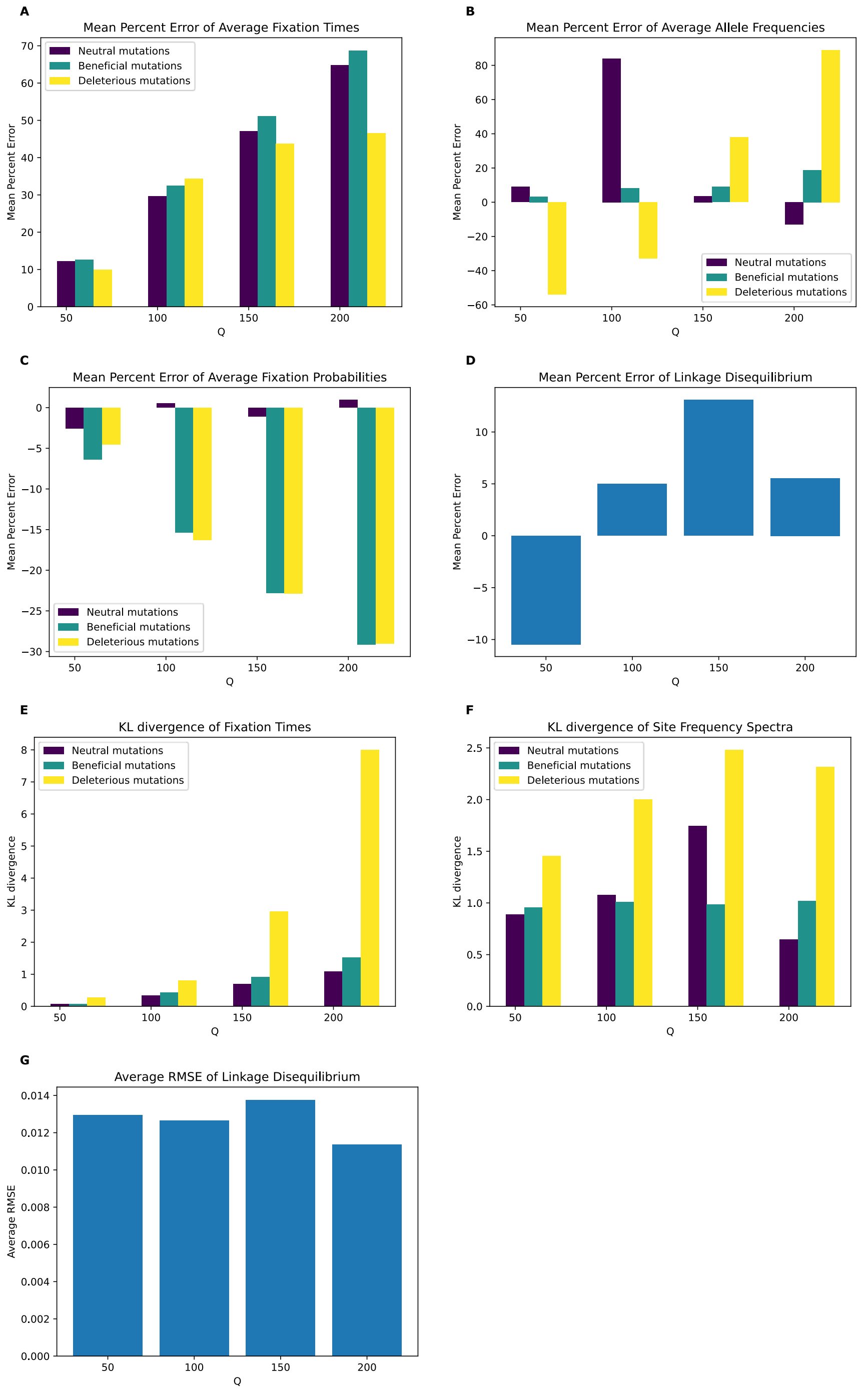

Figure S26. Measures of deviation for the *Drosophila* model using only 100 randomly-selected replicates of the full model.

(A) Mean percentage error of average fixation times for all mutation types. **(B)** Mean percentage error of average allele frequencies for all mutation types. **(C)** Mean percentage error of average fixation probabilities for all mutation types. **(D)** Mean percentage error of average LD as measured by r^2. **(E)** KL Divergence of fixation times for all mutation types. **(F)** KL divergence of the SFS for all mutation types. **(G)** Average RMSE of LD as measured by r^2 across 50 genomic bins.

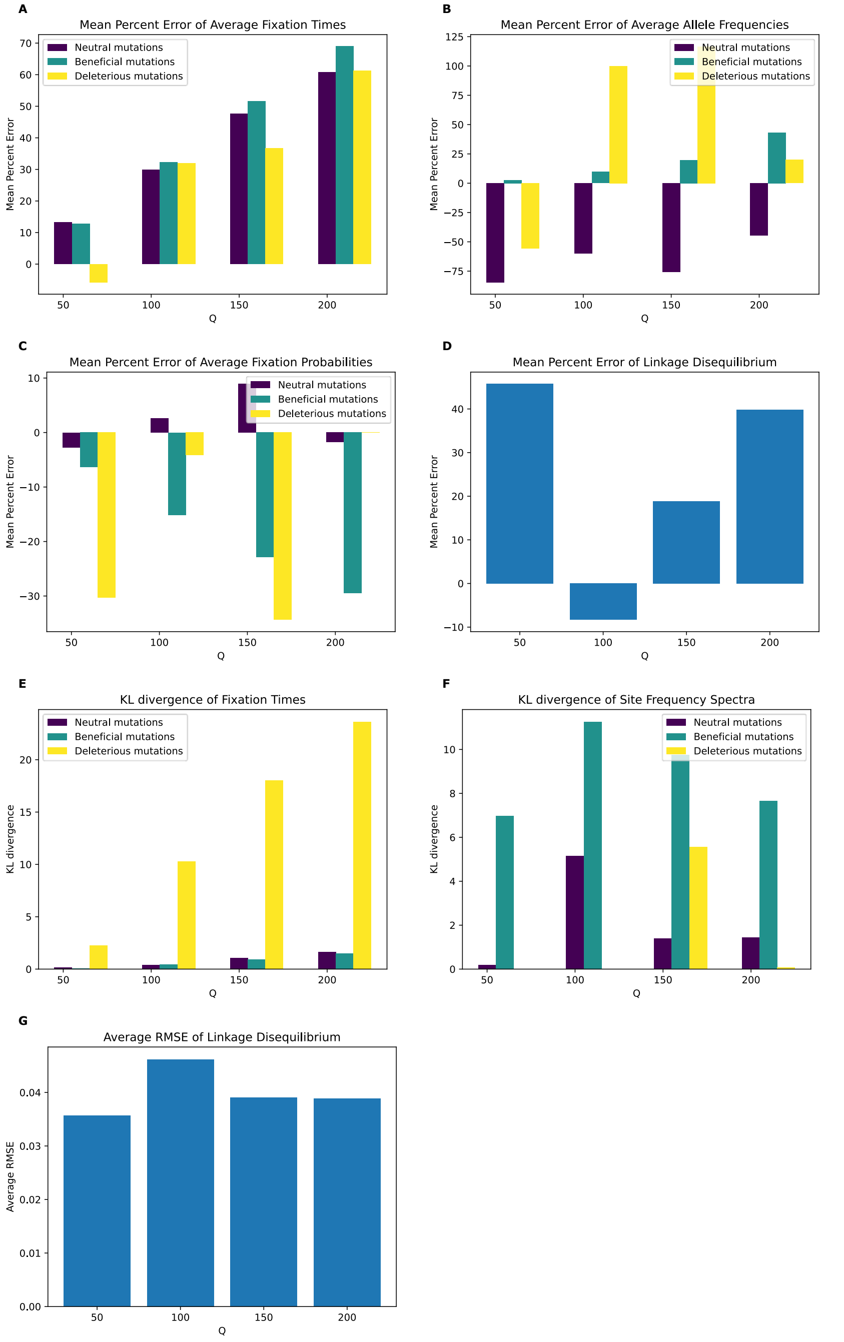

Figure S27. Measures of deviation for the *Drosophila* model using only 10 randomly-selected replicates of the full model.

(A) Mean percentage error of average fixation times for all mutation types. **(B)** Mean percentage error of average allele frequencies for all mutation types. **(C)** Mean percentage error of average fixation probabilities for all mutation types. **(D)** Mean percentage error of average LD as measured by r^2. **(E)** KL Divergence of fixation times for all mutation types. **(F)** KL divergence of the SFS for all mutation types. **(G)** Average RMSE of LD as measured by r^2 across 50 genomic bins.
